# Micro-patterned culture of iPSC-derived alveolar and airway cells distinguishes SARS-CoV-2 variants

**DOI:** 10.1016/j.stemcr.2024.02.011

**Published:** 2024-03-28

**Authors:** Atsushi Masui, Rina Hashimoto, Yasufumi Matsumura, Takuya Yamamoto, Miki Nagao, Takeshi Noda, Kazuo Takayama, Shimpei Gotoh

**Affiliations:** 1 Center for iPS Cell Research and Application (CiRA), Kyoto University, Kyoto 606-8507, Japan; 2 Department of Drug Discovery for Lung Diseases, Graduate School of Medicine, Kyoto University, Kyoto 606-8501, Japan; 3 Department of Clinical Laboratory Medicine, Graduate School of Medicine, Kyoto University, Kyoto 606-8507, Japan; 4 Medical-risk Avoidance Based on iPS Cells Team, RIKEN Center for Advanced Intelligence Project (AIP), Kyoto 606-8507, Japan; 5 Institute for the Advanced Study of Human Biology (WPI-ASHBi), Kyoto University, Kyoto 606-8501, Japan; 6 Laboratory of Ultrastructural Virology, Institute for Life and Medical Sciences, Kyoto University, Kyoto 606-8507, Japan; 7 Laboratory of Ultrastructural Virology, Graduate School of Biostudies, Kyoto University, Kyoto 606-8507, Japan

**Keywords:** human iPSC, alveolar epithelial cell, airway epithelial cell, micro-patterned, organoid, SARS-CoV-2, variant, image analysis

## Abstract

The emergence of severe acute respiratory syndrome-coronavirus-2 (SARS-CoV-2) variants necessitated a rapid evaluation system for their pathogenesis. Lung epithelial cells are their entry points; however, in addition to their limited source, the culture of human alveolar epithelial cells is especially complicated. Induced pluripotent stem cells (iPSCs) are an alternative source of human primary stem cells. Here, we report a model for distinguishing SARS-CoV-2 variants at high resolution, using separately induced iPSC-derived alveolar and airway cells in micro-patterned culture plates. The position-specific signals induced the apical-out alveolar type 2 and multiciliated airway cells at the periphery and center of the colonies, respectively. The infection studies in each lineage enabled profiling of the pathogenesis of SARS-CoV-2 variants: infection efficiency, tropism to alveolar and airway lineages, and their responses. These results indicate that this culture system is suitable for predicting the pathogenesis of emergent SARS-CoV-2 variants.

## Highlights

• Micro-patterned culture induces iPSC-derived lung organoids on the plate
• Apical-out alveolar and airway organoids are generated in micro-patterned culture
• Position-specific signals govern lineage differentiation within each colony
• Micro-patterned alveolar/airway organoids distinguish SARS-CoV-2 variants

## Graphical abstract

**Figure:**
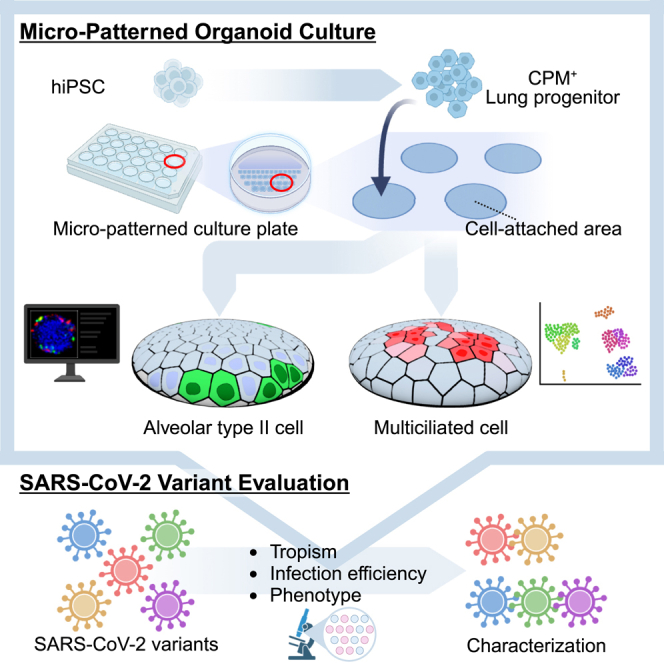

## Teaser

Micro-patterned culture is useful for inducing apical-out alveolar and airway organoids derived from iPSCs. Alveolar type 2 or multiciliated epithelial cells were generated position specifically within the uniformly defined colonies. The combination of alveolar and airway cells on the micro-patterned culture plates enabled us to characterize the SARS-CoV-2 variants quantitatively.

## Introduction

Alveolar type 2 epithelial cells (AT2s) are essential in tissue repair during lung injuries, highlighting the need to study AT2s using *in vitro* culture systems. Because *in vitro* culture of primary AT2s is still challenging, induced pluripotent stem cells (iPSCs) are useful sources of AT2s. Human iPSC-derived AT2s (iAT2s) and other pulmonary epithelial cells are stepwise differentiated via NKX2-1^+^ lung progenitor cells (LPCs) and require three-dimensional (3D) culture methods with extracellular matrix (ECM) components such as Matrigel for stable iAT2 induction and expansion (Gotoh et al., 2014; Hawkins et al., 2017; Jacob et al., 2017; Yamamoto et al., 2017). However, Matrigel-based 3D culture complicates organoid size control and direct image analysis.

The severe acute respiratory syndrome-coronavirus-2 (SARS-CoV-2) pandemic prompted the urgent establishment of *in vitro* culture systems of pulmonary epithelial cells, including AT2s, for infection modeling and pathophysiological studies. Because SARS-CoV-2 is a respiratory virus that rapidly mutates and transforms its properties, *in vivo*-like cell culture systems should aid in evaluating concerning variants. However, Matrigel-embedded alveolar and airway organoids were inconvenient for modeling SARS-CoV-2 infection because their apical epithelial surface, hosting the major SARS-CoV-2 receptor (angiotensin-converting enzyme 2 [ACE2]), faces inward (Mulay et al., 2021; Tamai et al., 2022). In early studies, air-liquid interface (ALI) culture was suitable for exposing the apical surface of iAT2 to SARS-CoV-2 infection (Huang et al., 2020). However, it was difficult to clearly distinguish SARS-CoV-2 variants.

Micro-patterned culture technology enables manufacturing cells or organoids in defined sizes and shapes using the culture substrate or cell adhesion area. Recently, dome-like lung bud organoids, comprising various pulmonary cells derived from human embryonic stem cells (ESCs), were created using this method and subjected to SARS-CoV-2 infection (Rosado-Olivieri et al., 2023). However, identifying airway and alveolar cells within the 3D structure posed a challenge. We then separated the infection models of airway and alveolar epithelial cells into micro-patterned culture plates, thereby enabling a highly differentiated state of multiciliated airway cells and AT2s and quantitively characterizing their infection by SARS-CoV-2 variants.

## Results

### Micro-patterned culture induces apical-out AT2s derived from iPSCs

**Figure 1:**
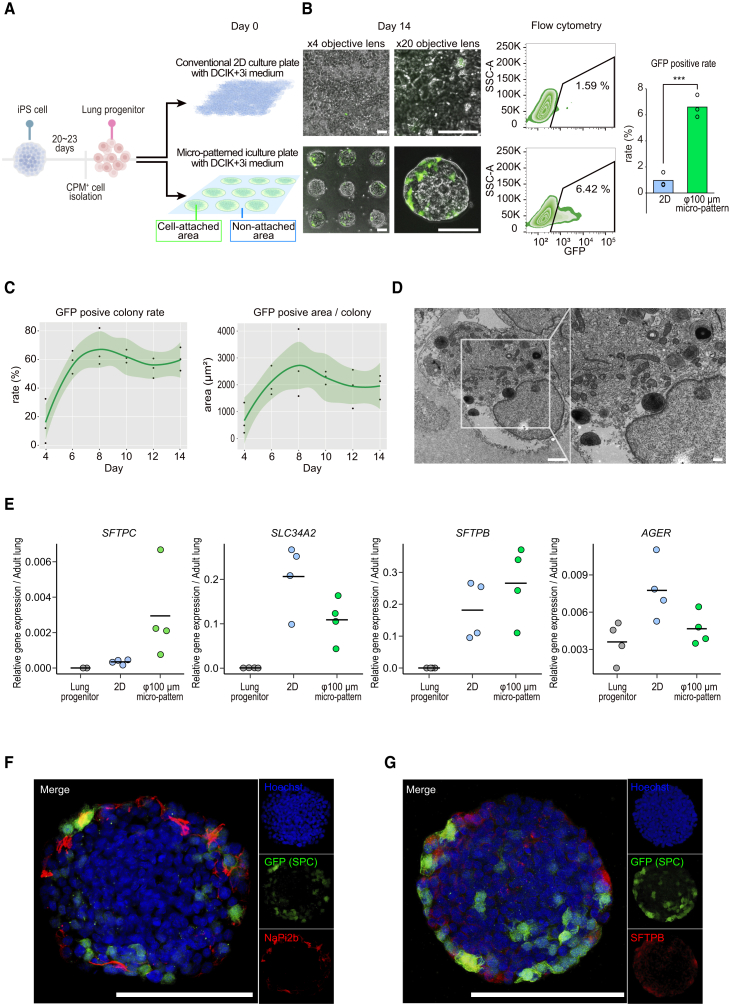
Micro-patterned culture of human iPSC-derived alveolar epithelial cells (A) Schematic diagram of human iPSC-derived alveolar epithelial cell development in a micro-patterned culture plate. The *SFTPC*^*GFP*^ reporter iPSC-derived LPCs sorted using anti-CPM IgG and magnetic beads were seeded onto the micro-patterned or conventional 2D culture plates (day 0) and cultured for 14 days. (B) Live-cell imaging of micro-patterned or conventional 2D culture at day 14 and quantification of the GFP^+^ cell rate. Data are means (n = 3 independent experiments). Unpaired 2-tailed Student’s t test; ^∗∗∗^p < 0.001. Scale bar: 100 μm. (C) Local regression plots of the rate of GFP^+^ colonies and GFP^+^ area within colonies (n = 3 independent experiments). (D) Transmission electron microscopy imaging of the alveolar epithelial cells in micro-patterned culture on day 14. Scale bars: 2 μm (left) and 500 nm (right). (E) Representative gene expression of human iPSC-derived alveolar epithelial cells in micro-patterned or conventional 2D culture analyzed using qRT-PCR (n = 4 independent experiments). (F and G) Immunofluorescent images of human iPSC-derived alveolar epithelial cells cultured on micro-patterned plates for 14 days. Double immunostaining of GFP with NaPi2b (F) and SFTPB (G) are shown. Scale bar: 100 μm.

Micro-patterned culture plates consist of nonadhesive areas coated with a nonadhesive polymer and circular cell-adhesion areas of regularly aligned 100- or 200-μm diameter. We investigated whether human iPSC-AT2s could be induced in these plates. Carboxypeptidase M (CPM)^+^ LPCs were induced stepwise from human *SFTPC*^*GFP*^ reporter iPSCs (B2-3) in which *SFTPC* is an AT2 marker (Gotoh et al., 2014), seeded onto these plates (2.5-dimensional culture plate, Tosoh, Japan), and maintained in the DCIK+3i medium, which we reported as fibroblast-free alveolarization medium (Yamamoto et al., 2017), resulting in the emergence of GFP^+^ cells in the micro-patterned culture plates. Furthermore, we compared the number of GFP^+^ cells in the micro-patterned culture plate with the conventional two-dimensional (2D) culture plate as a control after 14 days of culture (Figure 1A). Flow cytometry showed 6.6% ± 0.85% GFP^+^ cells on the micro-patterned plate, compared to 0.97% ± 0.54% in the submerged condition on the conventional 2D culture plates (Figure 1B). We monitored the number of GFP^+^ colonies and their area within colonies over time in micro-patterned cultures using image analysis (Figure 1C). GFP^+^ cells emerged 6 days after seeding and were maintained for 2 weeks. Transmission electron microscopy showed lamellar bodies—restoring the pulmonary surfactant—(Figure 1D). These results suggest that AT2s can be induced from human iPSCs on a micro-patterned culture plate.

In quantitative reverse transcriptase-PCR (qRT-PCR), the *SFTPC* of the cells cultured in micro-patterned plates, unlike that in 2D cultures, increased compared with that of LPCs, consistent with *SFTPC*^*GFP*^ reporter expression (Figure 1E). *SLC34A2* and *SFTPB—*also AT2 marker genes—were upregulated in the 2D and micro-patterned cultures. The *AGER* expression—an AT1 marker gene—in cells in micro-patterned plates did not change compared with that in LPCs. We found no upregulation of any other pulmonary epithelial cell marker gene in the micro-patterned cells compared with LPCs (Figure S1A). These results suggest that LPCs cultured with DCIK+3i medium in micro-patterned plates differentiated into AT2 cells, not specifically into other pulmonary epithelial cells. In immunofluorescence analysis, GFP was localized to the periphery of each colony, consistent with the expression of NaPi2b, a phosphate transporter expressed on the apical surface of AT2s, and SFTPB (Figures 1F and 1G). Confocal microscopy revealed that NaPi2b was expressed toward the outside of colonies (Figure S1B). These results indicate that the iPSC-AT2s induced in the micro-patterned plates were localized to the periphery of each colony, where their apical surface expressing NaPi2b faced outward.

### Region-specific program promotes the induction of iPSC-AT2s within the peripheral region of micro-patterned LPC colonies

**Figure 2:**
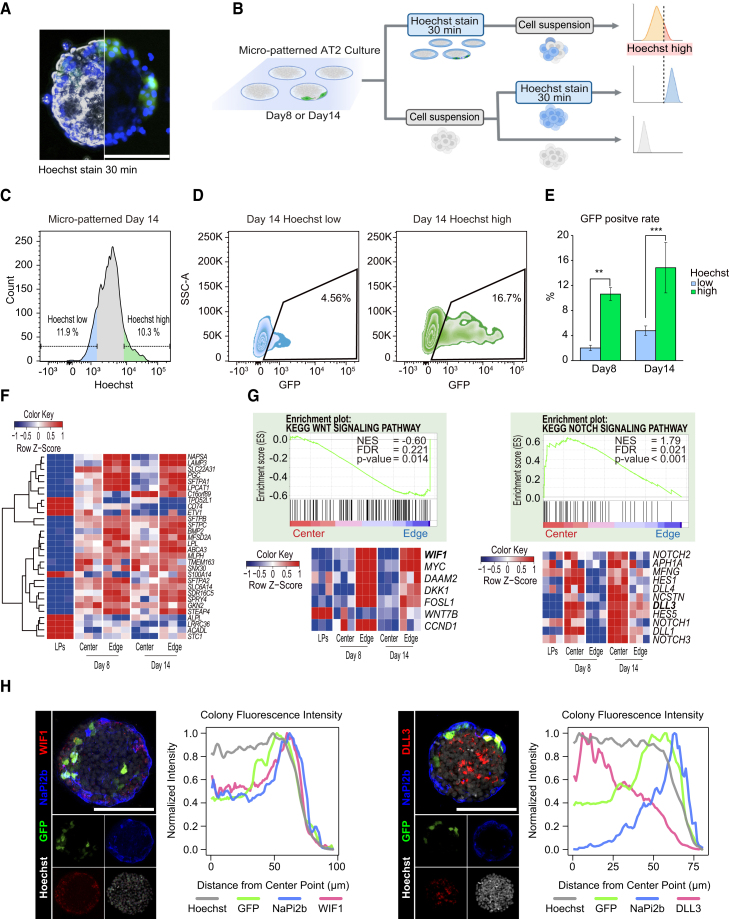
Alveolar epithelial cells are preferentially induced at the edge of the colonies in micro-patterned culture (A) Live-cell imaging of the Hoechst33342-stained colonies in the micro-patterned culture. Scale bar: 100 μm. (B) Experimental strategy for Hoechst-based separation of human iPSC-derived alveolar epithelial cells. Micro-patterned colonies were stained with Hoechst for 30 min and dissociated. Hoechst high and Hoechst low cells were isolated using FACS (top). For Hoechst high gating, the micro-patterned cells were dissociated into single cells, stained with Hoechst for 30 min, and analyzed using flow cytometry (center). Unstained cells were used as a negative control (bottom). (C) Histogram of fluorescence intensity of Hoeschst-stained micro-patterned culture cells analyzed using FACS. The rate of Hoechst low cells was defined as the percentage equivalent to that of Hoechst high cells on the opposite side of the histogram. (D and E) Rates of GFP^+^ cells in Hoechst low or Hoechst high cells (D). Data are mean ± SEM (n = 3 independent experiments) (E). Two-way ANOVA multiple comparisons: ^∗∗^p < 0.01; ^∗∗∗^p < 0.001. (F) Comparison of DEGs between the edge and center cell populations in the micro-patterned cultures. The transcriptomes of the edge and center cells in the micro-patterned culture were compared with those of LPCs, and DEGs were defined as genes satisfying the following criteria: padj <0.01, |log2 fold change| > 1. The heatmap presents the *Z* scores for AT2-related genes, calculated from log (transcripts per kilobase million [TPM] value) (n = 3 independent experiments). (G) GSEA analyses of the KEGG (Kyoto Encyclopedia of Genes and Genomes) WNT signaling pathway and the KEGG NOTCH signaling pathway, with each heatmap of the leading genes in the Edge cell population. Data from day 14 micro-patterned samples were ranked using p values comparing the Center and Edge cell populations using DESeq2. (H) Immunofluorescence image analyses showing position-specific signals. Line graphs illustrate the relationship between the distance from the colony center and the fluorescence intensity, representing average values from 5 colonies in 1 representative experiment. Scale bar: 100 μm.

We found that Hoechst33342 quickly stained live cells at the periphery of the colony. We isolated the periphery and central cell fractions from micro-patterned culture plates on days 8 or 14 using this phenomenon and counted the rate of GFP^+^ cells. The peripheral cells exhibited intense Hoechst33342 staining for up to 45 min (Figures 2A and S2A), in contrast to the central region’s lower intensity. The staining differences disappeared for 24 h of exposure to Hoechst33342 or staining after fixation with 4% paraformaldehyde (Figure S2A). After confirming the live-cell Hoechst staining, the cells were fixed and nuclei were stained with SYTO61. The DNA quantity determined based on the fluorescence intensity of SYTO61 did not correlate with Hoechst staining (Figure S2B). Subsequently, we collected the peripheral and central cells of the colonies via fluorescence-activated cell sorting (FACS) based on differences in Hoechst intensity (Figure 2B). The ratios of Hoechst high- and low-stained cells were 15.53% ± 1.23% and 16.80% ± 1.16% of the total cells on day 8, and 11.6% ± 0.96% and 11.9% ± 1.47% of the total cells on day 14, respectively (Figures 2C and S2B). On days 8 and 14, the percentage of GFP^+^ cells was significantly higher in the Hoechst high-stained cells from the colony periphery than in low-stained cells from the center (Figures 2D and 2E). This indicated that iPSC-AT2s in the micro-patterned plates were localized to the periphery of the colonies.

To verify the high Hoechst staining intensity of iPSC-AT2s in the peripheral cells of the colonies not derived from the *SFTPC*^*GFP*^ reporter cell line, we performed the same experiment using ChiPSC18, derived from another healthy donor (Figures S2C and S2D). After 2 weeks in micro-patterned culture, the periphery of the ChiPSC18 cell colony exhibited high Hoechst staining, like that of *SFTPC*^*GFP*^ reporter cells (Figure S2D). In addition, following recovery of the peripheral and central cells, significant enrichment of the AT2 marker gene was observed in Hoechst high cells (Figure S2E). Therefore, the periphery of the colonies in the micro-patterned culture was suitable for generating iPSC-AT2s from their LPCs.

We hypothesized that position-specific signals in each colony caused LPCs to differentiate into AT2s in the micro-patterned culture. To test this hypothesis, we performed RNA sequencing (RNA-seq) on day 0 LPCs and Hoechst low (Center) and Hoechst high (Edge) cells in the micro-patterned colonies on days 8 and 14 (Figure 2F). The differentially expressed genes (DEGs) between LPCs and day 14 Edge cells were compared, and 1,997 DEGs were identified (Figure S2F). AT2 marker genes (*SFTPC*, *SFTPB*, *ABCA3*, *SLC34A2*, and *NAPSA*) were significantly upregulated (Figure 2F). Although AT2 signatures were enriched in the 667 DEGs across all time points and cell positions (Figures S2G and S2H), they were markedly higher in the peripheral cells of the colonies in the micro-patterned culture. Furthermore, gene set enrichment analysis (GSEA) on day 14 revealed WNT signal enrichment in Edge cells (nominal p = 0.014, false discovery rate [FDR] q = 0.221) (Figure 2G). More specifically, *WIF1* and *DKK1*, the canonical WNT repressors, were upregulated in Edge cells. This suggests that canonical WNT signaling is suppressed in the peripheral regions of the colonies. In contrast, Notch signaling was activated in Center cells (nominal p < 0.001, FDR q = 0.021) (Figure 2G). Next, we performed fluorescent immunostaining to visualize the positional signals identified via RNA-seq. Image analysis revealed that WIF1 was localized to the periphery of the colonies. In contrast, DLL3, a NOTCH ligand, was localized in the center of the colonies (Figure 2H). Although Notch signaling is reportedly essential for airway and alveolar epithelial cell differentiation (Rock et al., 2011), GSEA detected enrichment of the early airway progenitor cell signature in Center cells (Figure S2H). Accordingly, we hypothesized that the central region of the colonies is suitable for airway epithelial cell differentiation, considering the position-specific preference for alveolar epithelial cell differentiation.

### Airway epithelial cells are differentiated in the central region of the micro-patterned colonies

**Figure 3:**
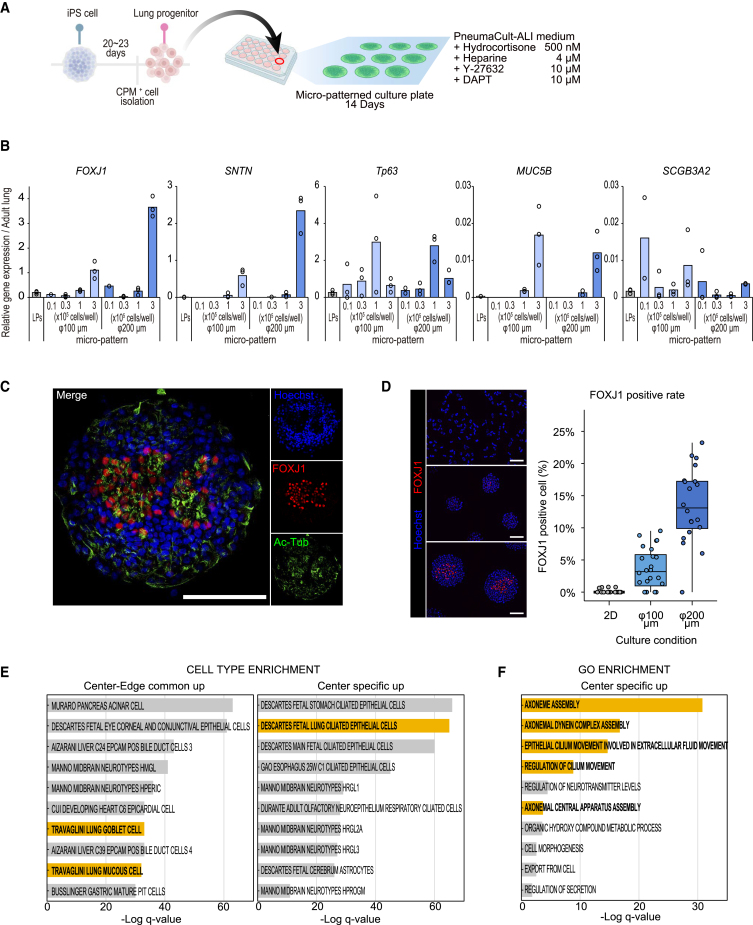
Airway epithelial cells are induced at the center of the colonies in micro-patterned culture (A) Schematic diagram of generating human iPSC-derived airway epithelial cells in a micro-patterned culture plate. LPCs were differentiated into airway epithelial cells on a micro-patterned plate for 14 days. (B) Representative gene expression of human iPSC-derived airway epithelial cells in the micro-patterned culture analyzed using qRT-PCR (n = 3 independent experiments). Data are presented as mean values. (C) Immunofluorescence imaging of the airway epithelial cells in the micro-patterned culture for 14 days. Scale bar: 100 μm. (D) Immunofluorescence imaging for quantifying the percentage of FOXJ1^+^ multiciliated cells (top: 2D culture, center: patterned culture φ100 μm, bottom: patterned culture φ200 μm). The boxplot shows the percentage of FOXJ1^+^ cells. Each dot represents the percentage of FOXJ1^+^ cells per field of view (2D) or per colony (micro-patterned culture) from 1 representative experiment. n = 20–24. Scale bar: 100 μm. (E) Cell-type enrichment analysis of the commonly upregulated genes in Center and Edge cells (1,003 genes) and the Center-specific upregulated DEGs (368 genes). (F) GO enrichment analysis of the Center-specific upregulated DEGs (368 genes).

We investigated whether airway epithelial cells differentiated in the micro-patterned culture (Figure 3A). In DCIK+3i medium, the transcriptome patterns resembled those of airway epithelial progenitor cells; however, multiciliated airway epithelial cells were not detected. Therefore, to promote their differentiation toward mature airway epithelial cells, LPCs seeded on the micro-patterned plates were cultured in PAL medium, which we previously reported useful for inducing multiciliated epithelial cells (Konishi et al., 2016). We observed moving cilia in the center of the colonies at approximately day 10 (Video S1). qRT-PCR revealed upregulations of *FOXJ1* and *SNTN*, multiciliated cell markers, on day 14. Their expression was higher in the 3 × 10^5^ cells/well condition with a spot diameter of φ200 μm than in the other conditions (Figure 3B). This could be because the colony center with ciliated epithelium was larger in the spot with φ200 μm than φ100 μm. We performed the subsequent experiments using a plate with φ100 μm spots to match the alveolar epithelial cell cultures. Other pulmonary epithelial cell marker genes showed unstable induction or low expression (Figure 3B). FOXJ1^+^ and acetylated tubulin^+^ multiciliated cells were identified in the colony center using immunofluorescence staining (Figures 3C and S3A). Acetylated tubulin localized to the cell apices in the colony center (Figure S3A), indicating outward-facing airway epithelial cells. Furthermore, we quantified the percentage of FOXJ1^+^ cells using image analysis, revealing a higher percentage in micro-patterned cultures compared to the conventional 2D culture, where the percentage of FOXJ1-positive cells was markedly lower in the submerged condition (Figure 3D).

Video S1. Human iPSC-derived LPCs cultured in the micro-patterned plate for 14 days to achieve airway differentiation involving multiple cilia observed at the center of the colony, related to Figure 3

Subsequently, we analyzed isolated Center and Edge cells via RNA-seq, as described for alveolar epithelial cells. Considering that multiciliated cells in the center of the colonies on day 14 exhibited high Hoechst intensity (Figure S3B), separating the Center and Edge cells using Hoechst intensity was challenging at this time point. Therefore, we isolated the Center and Edge cells on day 7, before multiciliated cells emerged, and compared the transcriptomes of LPCs on day 0 with those of Center and Edge cells in the micro-patterned colonies. Several marker genes for multiciliated and neuroendocrine cells were upregulated within the colony center on day 7 compared with the LPCs (Figure S3C). We identified 669 genes as Center-specific DEGs, 1,818 as Edge-specific DEGs, and 1,680 as common to Center and Edge cells (Figure S3D). The upregulated genes common to the Center and Edge cells enriched the goblet and mucous cell lineages. The Gene Ontology (GO) term for lung-ciliated epithelial cells was enriched in the upregulated center-specific DEGs (Figures 3E and S3E). Furthermore, GO enrichment analysis of Center-specific DEGs revealed multiple enriched terms associated with cilia (Figure 3F). Next, we compared the transcriptomes of Center cells cultured in the DCIK+3i and PAL media. A total of 1,378 DEGs were identified. Center cells in PAL medium showed significantly upregulated multiciliated cell-related genes and downregulation of AT2-related genes compared to DCIK+3i medium (Figure S3F). GSEA also identified “fetal ciliated epithelial cells” marker genes enrichment (nominal p < 0.001, FDR q < 0.001) (Figure S3G). These results suggest that multiciliated airway epithelial cells appear in the colony center, providing a suitable microenvironment for airway epithelial cell differentiation.

### Single-cell RNA-seq (scRNA-seq) analysis of the micro-patterned colonies of alveolar and airway epithelial cells

**Figure 4:**
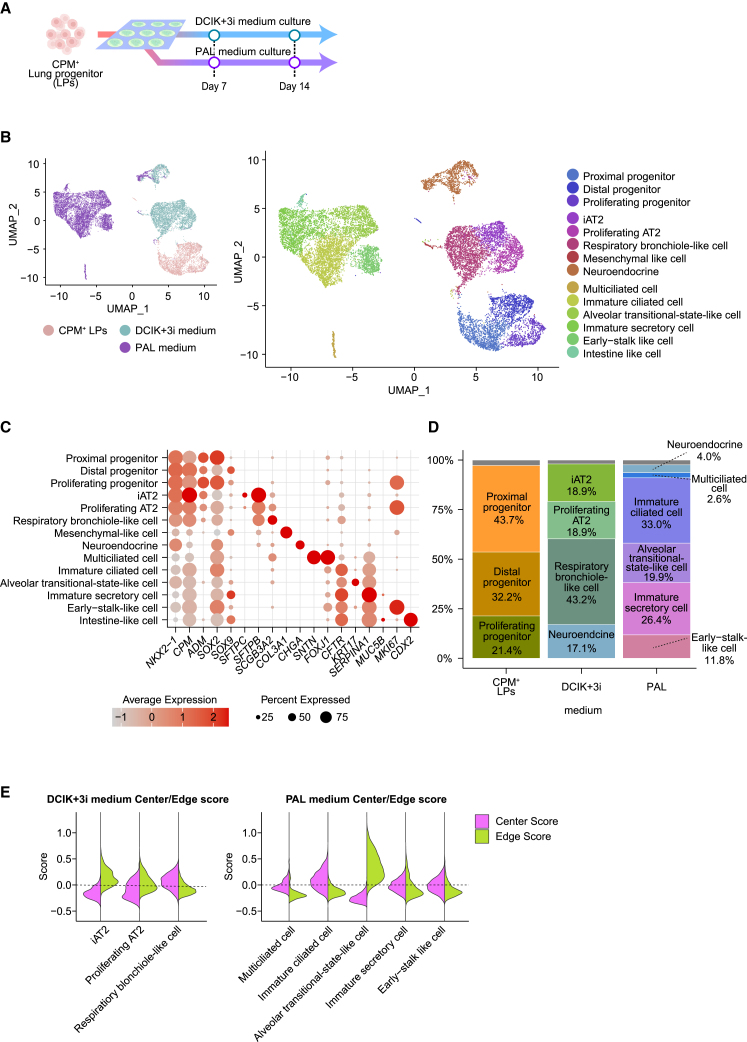
Cell-type scRNA-seq analysis of lung epithelial cells in the micro-patterned culture (A) Schematic diagram of the samples used for analysis. *SFTPC*^*GFP*^ knockin reporter iPSC-derived CPM^+^ LPCs were seeded onto micro-patterned culture plates and induced to differentiate into alveolar or airway epithelial cells. The LPCs and differentiated cells at each time point were dissociated for scRNA-seq. (B) UMAP projections of the consolidated data from all of the samples. Left: color-coded by culture conditions; right: color-coded by cell-type annotations after clustering. (C) Dot plot showing gene expressions in different cell types. The x axis lists selected genes, and the y axis categorizes cell types. Dot size indicates the percentage of cells expressing each gene, and color shade represents the magnitude of the average expression level of each gene. (D) Stacked bar chart showing the distribution of each cell type for LPCs and cultured samples. The first bar represents the LPCs labeled as CPM, and the subsequent bars represent samples cultured in DCiK+3i and PAL media, respectively. The color-coded segments and corresponding labels indicate the percentage of each cell type in the total population within each condition. (E) Violin plots comparing gene expression scores in the cell populations cultured in DCIK+3i medium (left) and PAL medium (right). Each plot represents the distribution of Center (purple) and Edge (green) scores for specific cell types, indicating the variability within the colony regions.

To characterize the specific cell types that make up the micro-patterned colonies, scRNA-seq was performed at each stage of CPM^+^ progenitor cell differentiation in DCIK+3i and PAL media on days 7 and 14 (Figure 4A). The analysis revealed distinct transcriptomes among the progenitor and micro-patterned cells in each medium (Figure 4B). Within the CPM^+^ progenitor cells, three subpopulations were identified, each predominantly expressing *SOX2*, *SOX9*, or *MKI67* (Figures 4C and 4D). These findings were consistent with the potential of CPM^+^ progenitor cells to differentiate into alveolar and airway epithelial lineages. The cell population cultured in DCIK+3i medium mainly comprised three clusters: (1) iAT2 cluster characterized by high expression of AT2 marker genes (*SFTPC*, *SLC34A2*, *SFTPA1*, and *SFTPB*); (2) proliferating AT2 cluster with low AT2 marker expression and positive for MKI67; and (3) respiratory bronchiole-like cell expressing *SFTPB* and *SCGB3A2* (Figure S4A) (Basil et al., 2022; Kadur Lakshminarasimha Murthy et al., 2022). The third cluster is suggested to represent cells located in the central region of the colonies by the high expression of genes characteristic of central cells (Table S1), as observed in the bulk RNA-seq results (Figure 4E). The cells cultured in PAL medium contained a multiciliated cluster expressing markers, such as *SNTN* and *FOXJ1* (Figure 4C). The remaining clusters, still immature, expressed genes associated with ciliated epithelial cells (*FOXJ1*, *DRC1*, and *CCNO*) or immature secretory cells (*SERPINA1* and *MUC5B*) (Figure S4B). In addition, stalk cell-like clusters, similar to those described by He et al. (2022), were observed, indicating the presence of cells in an earlier developmental stage compared to the previously described clusters (Figure S4C). Cells in the peripheral region formed an independent cluster that interestingly resembled the transcriptome of the intermediate cells differentiated from AT2s to AT1s (Figure S4D). This cluster was annotated as alveolar transitional state-like cell, previously reported in the literature (Kobayashi et al., 2020). Neuroendocrine cells were detected in DCIK+3i and PAL cultures. Mesenchymal-like and intestinal epithelial-like cells were rare in these cultures (Figures 4D and S4E). We assessed the similarity of scRNA-seq transcriptomes data of iAT2 and multiciliated cells induced in micro-patterned culture to snRNA-seq transcriptomes data of primary pulmonary epithelial cells at the fetal, juvenile, and adult stages (Wang et al., 2020) using uniform manifold approximation and projection (UMAP) (Figure S5). Our findings showed that iAT2 cells aligned more closely with fetal or juvenile AT2 cells (Figures S5A and S5B), but not adult cells (Figure S5C), indicating substantial resemblance to early-stage AT2 cells. Despite the limitations posed by integrating the different analytical methods, our findings showed that iAT2 cells aligned more closely with fetal or juvenile AT2 cells (Figures S5A and S5B), not adults (Figure S5C), indicating substantial resemblance to early-stage AT2 cells. In contrast, multiciliated cells overlapped with those from all stages, suggesting a high similarity to adult ones.

On day 7 of the DCIK+3i culture, 57.0% of the cells were iAT2 or proliferating AT2, and 31.7% were *SFTPB*^+^*SCGB3A2*^+^ cells, mainly AT2-like and respiratory bronchiolar cells (Figure S4E). By day 14, the proportion of iAT2 and proliferating AT2 decreased to 16.0%, whereas *SFTPC* was upregulated, indicating a more refined AT2 cell population (Figure S4F). On day 7 in the PAL culture, 49.7% of the population comprised multiciliated or immature ciliated cells, whereas the rest were more immature airway epithelial cells. By day 14, the proportion of ciliated cell clusters decreased to 13.5%, whereas that of immature secretory cells increased to 58.9% (Figure S4E).

### Micro-patterned culture is suitable for modeling the SARS-CoV-2 infection model

**Figure 5:**
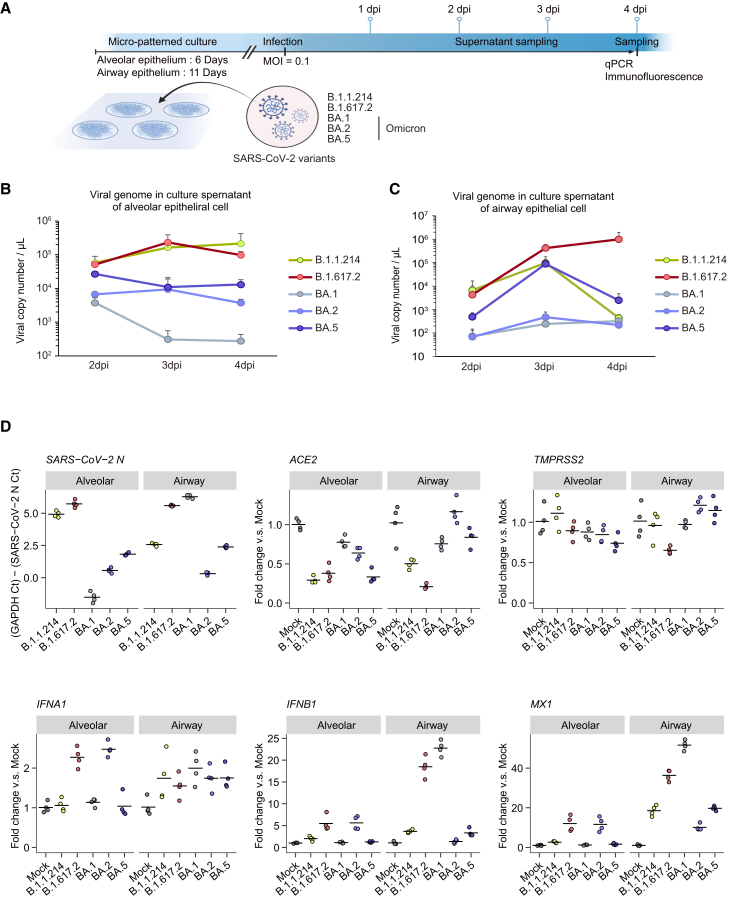
Tropism of SARS-CoV-2 variants in iPSC-derived alveolar and airway epithelial cells in the micro-patterned culture (A) Schematic diagram of SARS-CoV-2 infection experiments. The iPSC-derived alveolar and airway epithelial cells in the micro-patterned culture plate were infected with each SARS-CoV-2 variant. (B and C) The viral RNA copy number in the cell culture supernatant of iPSC-derived alveolar (B) and airway (C) epithelial cells at 2, 3, and 4 dpi were measured using qRT-PCR. (D)Representative gene expression levels of iPSC-derived alveolar and airway cells at 4 dpi measured using qRT-PCR.

We conducted infection experiments with five SARS-CoV-2 variants (MOI 0.1) in micro-patterned cultures: B.1.1.214, B.1.617.2 (delta), BA.1, BA.2, and BA.5. The alveolar epithelial cells were infected on day 6, once GFP^+^ cells appeared, and airway epithelial cells were infected on day 11, after multiciliated cell detection (Figure 5A). Viral amplification in alveolar and airway epithelial cells was assessed based on detectable viral genomes in the supernatant (Figures 5B and 5C). In alveolar epithelial cells, the B.1.1.214- and B.1.617.2-infected groups released many viruses continuously (maximum: 2.14 × 10^5^ and 2.34 × 10^5^ viral copy number/μL, respectively). In contrast, the Omicron variants (BA.1, BA.2, and BA.5) released fewer viruses (maximum: 3.74 × 10^3^, 9.38 × 10^3^, and 2.68 × 10^4^ viral copy number/μL, respectively). In airway epithelial cells, B.1.617.2 continuously released the most virus (maximum: 9.85 × 10^5^ viral copy number/μL); however, that released by B.1.1.214 and BA.5 was similar 3 days postinfection (dpi) (9.74 × 10^5^ and 8.80 × 10^5^ viral copy number/μL, respectively). Meanwhile, viral genome levels decreased at 4 dpi (0.437 × 10^3^ and 2.64 × 10^3^ viral copy number/μL, respectively). In addition, viral release into the supernatant from BA.1- and BA.2-infected airway cells was negligible (maximum: 321 and 219 viral copy number/μL, respectively) (Figure 5C).

Next, we collected intracellular RNA from alveolar and airway epithelial cells at 4 dpi and assessed viral gene expression via qRT-PCR (Figure 5D). Alveolar cells infected with B.1.1.214 and B.1.617.2 showed increased *SARS-CoV-2 N*, whereas in airway cells, only B.1.617.2 infected cells led to a persistent increase, indicating high tropism of this variant for both cells. *SARS-CoV-2 N* levels in Omicron variant-infected alveolar epithelial cells were lower, consistent with the virus release results. Meanwhile, BA.1, which rarely infects alveolar epithelial cells, showed *SARS-CoV-2 N* levels comparable to those of B.1.617.2 in airway epithelial cells. The discrepancy between the low virus release and high *SARS-CoV-2 N* levels in BA.1-infected airway cells may indicate differences in the extracellular virus release mechanisms of BA.1. *ACE2*—gene encoding a SARS-CoV-2 receptor—was downregulated in alveolar and airway epithelial cells, mirroring *SARS-CoV-2 N* levels. The levels of interferon response-related genes (*IFNA1*, *IFNB1*, and *MX1*) were elevated in cells infected by the B.1.617.2 and BA.2 variants. These results, not closely linked to *SARS-CoV-2 N* levels, may reflect differences in the immune response intensity of each variant. Therefore, our model could distinguish between SARS-CoV-2 variants based on unique infection efficiency and the initiation of innate immune responses in alveolar and airway systems.

### Quantification of SARS-CoV-2 variant tropism using micro-patterned alveolar and airway cells

**Figure 6:**
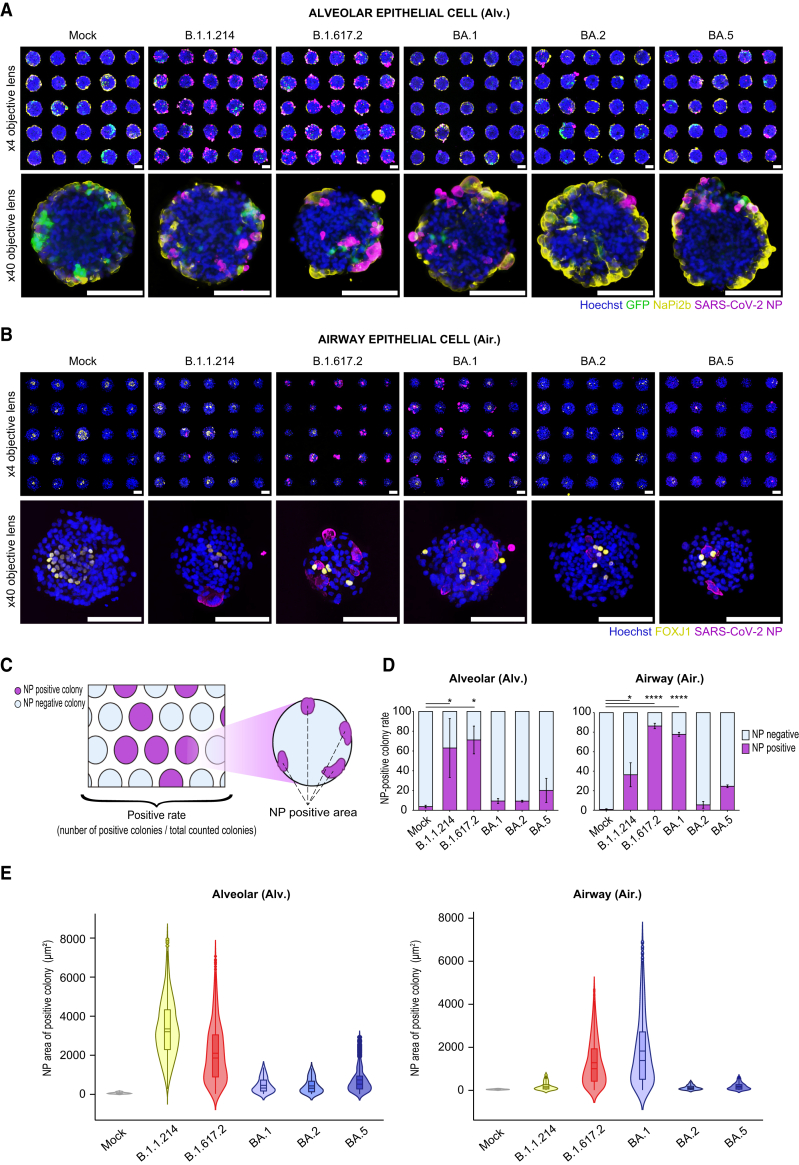
Image analyses of iPSC-derived alveolar and airway epithelial cells to characterize SARS-CoV-2 variants (A and B) Immunofluorescent images of SARS-CoV-2-infected alveolar (A) and airway (B) epithelial cells in a micro-patterned culture plate at 4 dpi. Scale bar: 100 μm. Top: low-magnification images; bottom: high-magnification images. (C) Schematic of a SARS-CoV-2 NP^+^ colony. The number of SARS-CoV-2 NP^+^ colonies was defined as the percentage of colonies positive for SARS-CoV-2 NP. The SARS-CoV-2 NP^+^ area was defined as the area of SARS-CoV-2 NP signals in the N signal^+^ colonies. (D) Rate of SARS-CoV-2 NP^+^ colonies in alveolar and airway epithelial cells infected with each SARS-CoV-2 variant. Data are mean ± SEM (n = 3 independent experiments). Two-way ANOVA with Dunnett’s multiple comparison tests; ^∗^p < 0.05, ∗∗∗∗p < 0.0001. (E) Violin plots of SARS-CoV-2 NP^+^ areas in alveolar and airway epithelial cells infected with each SARS-CoV-2 variant.

SARS-CoV-2 infection was quantified at the protein level using immunofluorescence analysis. Lineage markers and SARS-CoV-2 nucleocapsid protein (NP) were analyzed in micro-patterned alveolar and airway epithelial cell cultures. SARS-CoV-2 NP was detected in the alveolar and airway epithelial cells of the SARS-CoV-2-infected group (Figures 6A and 6B). Subsequently, we segmented alveolar epithelial cell colonies into center and periphery and calculated NP^+^ area percentages (Figure S6A). NP^+^ cells were preferentially localized at the periphery of the colonies, where AT2s were detected (Figure S6B). Furthermore, we developed an infection-efficiency index based on quantitative image analysis. We considered the viral infection of numerous colonies stochastic and calculated the percentage of NP^+^ colonies as an indicator of the tropism of each variant (Figure 6C). Approximately 1,000 colonies were detected in each well of the 24-well micro-patterned plate. A high percentage of SARS-CoV-2 NP^+^ colonies was calculated in the B.1.1.214- and B.1.617.2-infected groups (63.0% ± 29.7% and 71.2% ± 14.1%, respectively). In contrast, the Omicron variants showed lower percentages: 9.4% ± 2.4% (BA.1), 9.3% ± 1.0% (BA.2), and 20.1% ± 12.2% (BA.5) (Figure 6D). These results correlated with the amount of virus released into the culture supernatant (Figure 5B) and intracellular *SARS-CoV-2 N* levels (Figure 5C). Hence, the percentage of SARS-CoV-2 NP^+^ colonies effectively reflected the stochastic SARS-CoV-2 infection rate in target cells. Next, we quantified their NP^+^ areas of NP^+^ colonies (Figure 6C) as an index of intracellular viral proliferation and local propagation. The mean NP^+^ areas were of the 3,350 μm^2^ (B.1.1.214) and 2,102 μm^2^ (B.1.617.2), larger than the Omicron variants 461.5 μm^2^ (BA.1), 429.7 μm^2^ (BA.2), and 739.4 μm^2^ (BA.5) (Figure 6E). These results, correlating with intracellular *SARS-CoV-2 N* levels, indicate that both the percentage of NP^+^ colonies and NP^+^ areas are reliable indicators of variant tropism. The percentage of SARS-CoV-2 NP^+^ colonies in micro-patterned airway epithelial cells was high following B.1.617.2 and BA.1 infection (86.3% ± 2.8% and 77.8% ± 2.0%, respectively) and low after B.1.1.214 (36.4% ± 12.3%), BA.2 (5.6% ± 3.5%) and BA.5 (24.6% ± 1.4%) infection (Figure 6D). Mean NP^+^ areas for the B.1.617.2- and BA.1-infected groups were 1,341 and 1,558 μm^2^, respectively, whereas those for B.1.1.214-, BA.2-, and BA.5-infected groups were 399.7, 106.8, and 226.2 μm^2^, respectively (Figure 6E). These results correlated with intracellular *SARS-CoV-2 N* levels (Figure 6D). Conclusively, the high-tropism variants differed between alveolar and airway epithelial cells, indicating that SARS-CoV-2 tropism depends on the target cell lineages and variants.

### Micro-patterned culture system distinguishes the pathogenesis of each SARS-CoV-2 variant

**Figure 7:**
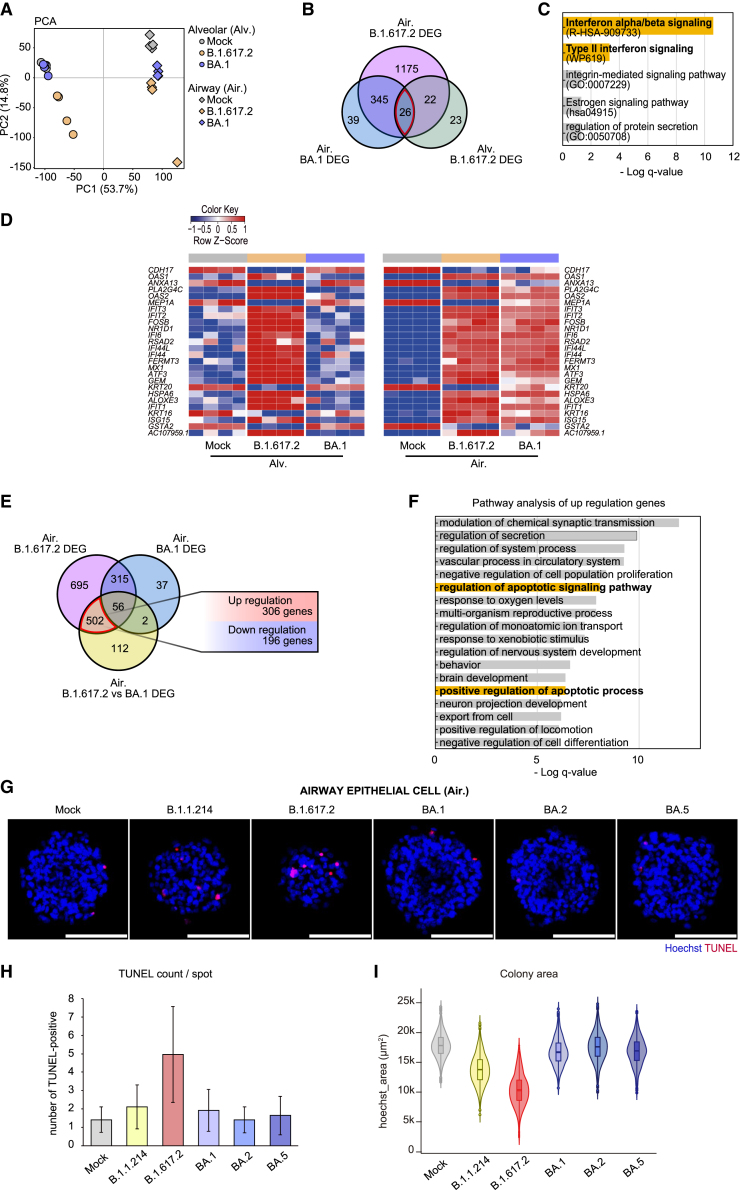
Comparing SARS-CoV-2 variants elucidates viral pathogenicity (A) PCA of the transcriptomes of the alveolar and airway epithelial cells infected with or without SARS-CoV-2: B.1.617.2 or BA.1. log (TPM value) was used for the PCA. (B) A total of 26 genes were extracted as commonly altered in DEGs between mock- and B.1.617.2-infected alveolar epithelial cells and those among mock-, B.1.617.2-, and BA.1-infected airway epithelial cells. (C) GO analysis of the 26 extracted genes. (D) Heatmaps presented with *Z* scores of the 26 extracted genes from the alveolar (left) and airway (right) epithelial cells infected with or without SARS-CoV-2: B.1.617.2 or BA.1. *Z* scores were calculated from log (TPM value) (n = 4 biological replicates). (E) The 502 genes were extracted from DEGs between mock vs. B.1.617.2 infection in the airway epithelial cells, excluding DEGs between mock vs. BA.1. The 509 genes comprised 306 upregulated and 196 downregulated genes. (F) GO analysis of the 306 upregulated genes. (G) Representative TUNEL staining images of the SARS-CoV-2-infected airway epithelial cells in the micro-patterned culture plate at 4 dpi. Scale bar: 100 μm. (H) The number of TUNEL^+^ cells within the SARS-CoV-2-infected airway epithelial cell colonies in the micro-patterned culture plate at 4 dpi. Data are mean ± SD. The number of colonies ranged from 498 to 1,110 from 1 representative experiment. (I) Violin plot of the Hoechst area of airway epithelial cells infected with each variant. All of the colonies were quantified. The number of colonies ranged from 1,133 to 1,169 from 1 representative experiment.

We next compared the transcriptomes of B.1.617.2- and BA.1-infected cells using principal-component analysis (PCA), revealing that SARS-CoV-2 infection shifted the plots parallel to the PC2 axis (Figure 7A). The BA.1-exposed micro-patterned alveolar cell transcriptomes overlapped with the mock control, corresponding to the low tropism of BA.1 for alveolar epithelial cells. These results suggest that the transcriptomic changes after SARS-CoV-2 infection were similar in micro-patterned alveolar and airway epithelial cells. We performed pathway enrichment analysis on the 26 DEGs common to the three infection groups: B.1.617.2 in micro-patterned alveolar and airway epithelial cells and BA.1 in micro-patterned airway epithelial cells (Figure 7B). In the common DEGs, the gene set related to IFN reactions was enriched (Figures 7C and 7D). These results suggested that IFN responses were induced in the alveolar and airway epithelial cells after SARS-CoV-2 infection. Subsequently, we compared the transcriptomes of the airway epithelial cells infected with B.1.617.2 or BA.1, which exhibited comparable tropism, although virus release was relatively limited in the BA.1 infection culture (Figure 7E). We investigated 502 DEGs specific to the B.1.617.2-infected airway epithelial cells. The regulation of apoptotic signaling pathway and the positive regulation of apoptotic process were enriched in the 306 upregulation genes (Figure 7F). We then performed TUNEL staining for the cells at 4 dpi in the SARS-CoV-2 mutant infection experiment (Figures 7G and S7A). More TUNEL^+^ cells were observed in the B.1.617.2-infected group (4.96 ± 2.61 count/colony) than in the other variant-infected groups (Figures 7H and S7B). In addition, the colony size of the B.1.617.2-infected group (10.4 ± 2.64 × 10^3^ μm^2^) was smaller than that of other variant-infected groups and served as a surrogate indicator of apoptosis (Figure 7I). These results suggest that the B.1.617.2 variant induces apoptosis in micro-patterned airway epithelial cells and that the micro-patterned culture system can be adopted to detect unique variant phenotypes.

## Discussion

AT2s are notorious for their rapid change when cultured on plastic surfaces (Borok et al., 1998; Foster et al., 2007). Although successfully grown in 3D cultures embedded in ECM, such as Matrigel, their methods and image analyses are still complicated. In addition, AT2s in spheroids within Matrigel orient their apical surfaces toward the lumen, representing a considerable obstacle to modeling SARS-CoV-2 infection (Mulay et al., 2021; Salahudeen et al., 2020; Tamai et al., 2022). Micro-patterned culture is expected to address these issues. In the present study, we demonstrated that the separately cultured iPSC-derived alveolar and airway epithelial cells in micro-patterned plates could distinguish SARS-CoV-2 variants. The cells within the heterogeneous cell colonies on micro-patterned plates seem to be from two to four overlaid layers (Video S2) with high cell density. Importantly, iAT2s in micro-patterned cultures were apical-out, exposing their apical surface to the medium without the additional steps of enzymatic digestion of the Matrigel or culture in suspension. In contrast, highly confluent cells at the center of the colonies were suitable for inducing multiciliated airway cells.

Video S2. 3D reconstructed imaging of the alveolar epithelial cells cultured in the micro-patterned plate, related to Figure S1B

Distinct staining properties of various dyes, including Hoechst, within the center and periphery of micro-patterned iPSC colonies have been reported (Kim et al., 2022). This finding was replicated in our micro-patterned cultures, aiding in separating central and peripheral colony regions for transcriptome analysis and elucidating that the position-specific signals differed between the center and periphery of the colonies. Specifically, the WNT signal was suppressed at the periphery of the colonies, consistent with the previous finding that appropriate WNT inhibition promoted AT2 differentiation (Jacob et al., 2017). Meanwhile, RNA-seq analysis revealed the upregulation of Notch signaling in the central region, indicating its contribution to the differentiation of airway lineages. The enrichments of NOTCH signaling- and airway progenitor cell-related genes in the center of the colony are consistent with the early stages of airway development in mice (Guha et al., 2012; Stupnikov et al., 2019). In addition, the suppression of NOTCH signaling by DAPT (a γ-secretase inhibitor) in the airway epithelial induction medium promoted multiciliated cell differentiation, consistent with a previous report (Rock et al., 2011). These results support the notion that position-specific signals within the colonies of the micro-patterned culture plates can facilitate alveolar and airway cell induction.

The different infection profiles of SARS-CoV-2 variants were reflected in various parameters. For example, quantifying virus release into the culture supernatant recapitulated viral spread in the lung epithelium. Moreover, analysis of intracellular gene expression levels, quantification of the number and size of infected colonies, and assessment of the abundance of viral antigen and other essential markers were performed using the two major lung lineages in a micro-patterned culture. This study proposes an unprecedented parameter, the percentage of SARS-CoV-2 N^+^ colonies, as an index of infection efficiency. This hypothesis is based on the idea that colony viral infection occurs stochastically and that the ratio of infected colonies depends on the viral tropism for cells.

Our results indicated that each variant had different infection efficiencies in the two major lung regions, alveolar and airway lineages, and that the efficient infection of B.1.1.214 and B.1.617.2 in iPSC-derived alveolar epithelial cells was consistent with the clinical phenotypes of the early SARS-CoV-2 variants, which frequently caused virus-induced pneumonia (van Doremalen et al., 2022). In addition, the Delta variant (B.1.617.2) released the largest number of viral genomes and proliferated the most actively among the SARS-CoV-2 variants (B.1.1.214, B.1.617.2, BA.1, BA.2, and BA.5) in the airway and alveolar lineages, and induced apoptosis in airway epithelial cells. These features of B.1.617.2 may be associated with its invasiveness, which caused severe pneumonia at a higher frequency than the other variants (Li et al., 2020). Furthermore, our results show that the Omicron variant (BA.1, BA.2, and BA.5) has distinct features. BA.2 shows higher viral release than BA.1 in alveolar epithelial cells, whereas BA.5 exhibits the strongest viral release among the three variants and relatively high intracellular proliferation capacity in the airway and alveolar lineages. These differences without the immune system may suggest that evading immunity was not the sole reason for the main pandemic variant being replaced by BA.1 to BA.2 and BA.5 (Kimura et al., 2022). Our study differs from a previous report on patterning lung bud cultures derived from human ESCs (Rosado-Olivieri et al., 2023) because our system enables detailed analysis of SARS-CoV-2 variants by separately inducing alveolar and airway epithelial cells with each lineage benchmarked. Hence, our system has the advantage of supporting the evaluation of differences among variants, particularly in terms of their tropism and phenotypic characteristics.

In conclusion, our culture system enables the robust induction of iPSC-derived alveolar and airway cells. The position-specific appearance of AT2s and multiciliated airway cells allows for the characterization of crucial signals required for their induction. Our results indicate that this strategy can be effectively adopted to analyze the pathogenesis of known SARS-CoV-2 variants, but also for characterizing the emerging SARS-CoV-2 variants. In this way, our system can be applied to predict the severity of new variants before they become prevalent.

## Experimental procedures

### Resource availability

#### Lead contact

Further information and requests for resources and reagents should be directed to and will be fulfilled by the corresponding authors. The lead contact is Shimpei Gotoh (gotoh.shimpei.5m@kyoto-u.ac.jp).

#### Materials availability

The materials included in this study are available from the corresponding authors upon reasonable request.

#### Data and code availability

The accession numbers for the RNA-seq and scRNA-seq raw data reported in the present study are GEO: GSE236842 and GSE249762.

### Micro-patterned plate culture

The magnetic-activated cell sorting (MACS) -isolated LPCs were seeded in 24-well micro-patterned culture plates (2.5D culture plates) (Tosoh) with multiple round cell-attachment areas in a 100- or 200-μm diameter precoated with 0.5 μg/cm^2^ of iMatrix-511 silk (TaKaRa Bio, 892021) at cell densities ranging from 0.1 to 3 × 10^5^ cells/well. The LPCs were differentiated into alveolar epithelial cells in DCIK+3i medium: Ham’s F12 (Fujifilm Wako, 087-08335) containing 50 nM dexamethasone (Sigma-Aldrich, D4902), 100 μM 8-Br-cAMP (Biolog Life Science Institute, B007), 100 μM 3-isobutyl-1-methylxanthine (Fujifilm Wako, 099–03411), 10 ng/mL keratinocyte growth factor (KGF), 1% B-27 supplement, 0.25% bovine albumin fraction V (Thermo Fisher Scientific, 15260-037), 15 mM HEPES (Thermo Fisher Scientific, 17557-94), 0.8 mM CaCl_2_ (Fujifilm Wako, 036-19731), 0.1% ITS premix (Corning, 354352), 50 U/mL penicillin/streptomycin, 3 μM CHIR-99021, 10 μM SB431542 (Fujifilm Wako, 198-16543), and 10 μM Y27632 (LC Laboratories, Y-5301). The LPCs were differentiated into airway epithelial cells in the medium as follows: PneumaCult-ALI medium (Veritas, ST-05001) containing 500 nM hydrocortisone (Sigma-Aldrich, H4001), 4 μM heparin (Nacalai Tesque, 17513-54), and 10 μM Y27632 for the initial 2 days and the same medium but supplemented with 10 μM DAPT (“PAL medium”) for the remaining period. The medium was changed every 2 days.

### SARS-CoV-2 variant infection postmicro-patterning culture

Alveolar or airway epithelial cells differentiated in 24-well micro-patterned culture plates were infected with 0.1 MOI SARS-CoV-2 for 2 h. Infected alveolar or airway epithelial cells were cultured in DCIK+3i medium or PneumaCult-ALI medium for 4 days. The culture medium was changed daily after infection.

### Statistical analysis

Data are presented as mean ± SEM. The number of biological replicates and statistical tests is described in each figure legend. All of the statistical tests were performed using Prism7 software (GraphPad). Statistical significance was set at p < 0.05.

## Supplemental information

Document S1. Figures S1–S7, Tables S1–S3, and supplemental experimental procedures

Document S4. Article plus supplemental information
