# Supplementary material for: Micro-patterned culture of iPSC-derived alveolar and airway cells distinguishes SARS-CoV-2 variants
Source: Stem Cell Reports. 2024 Mar 28;19(4):545–61. doi: 10.1016/j.stemcr.2024.02.011 (PMC11096626; doi:10.1016/j.stemcr.2024.02.011)
Supplement: Document S1. Figures S1–S7, Tables S1–S3, and supplemental experimental procedures [file mmc1.pdf]

Stem Cell Reports, Volume *19*

Supplemental Information

Micro-patterned culture of iPSC-derived alveolar and airway cells distinguishes SARS-CoV-2 variants

Atsushi Masui, Rina Hashimoto, Yasufumi Matsumura, Takuya Yamamoto, Miki Nagao, Takeshi Noda, Kazuo Takayama, and Shimpei Gotoh

SUPPLEMENTAL INFORMATION

Supplemental Figures

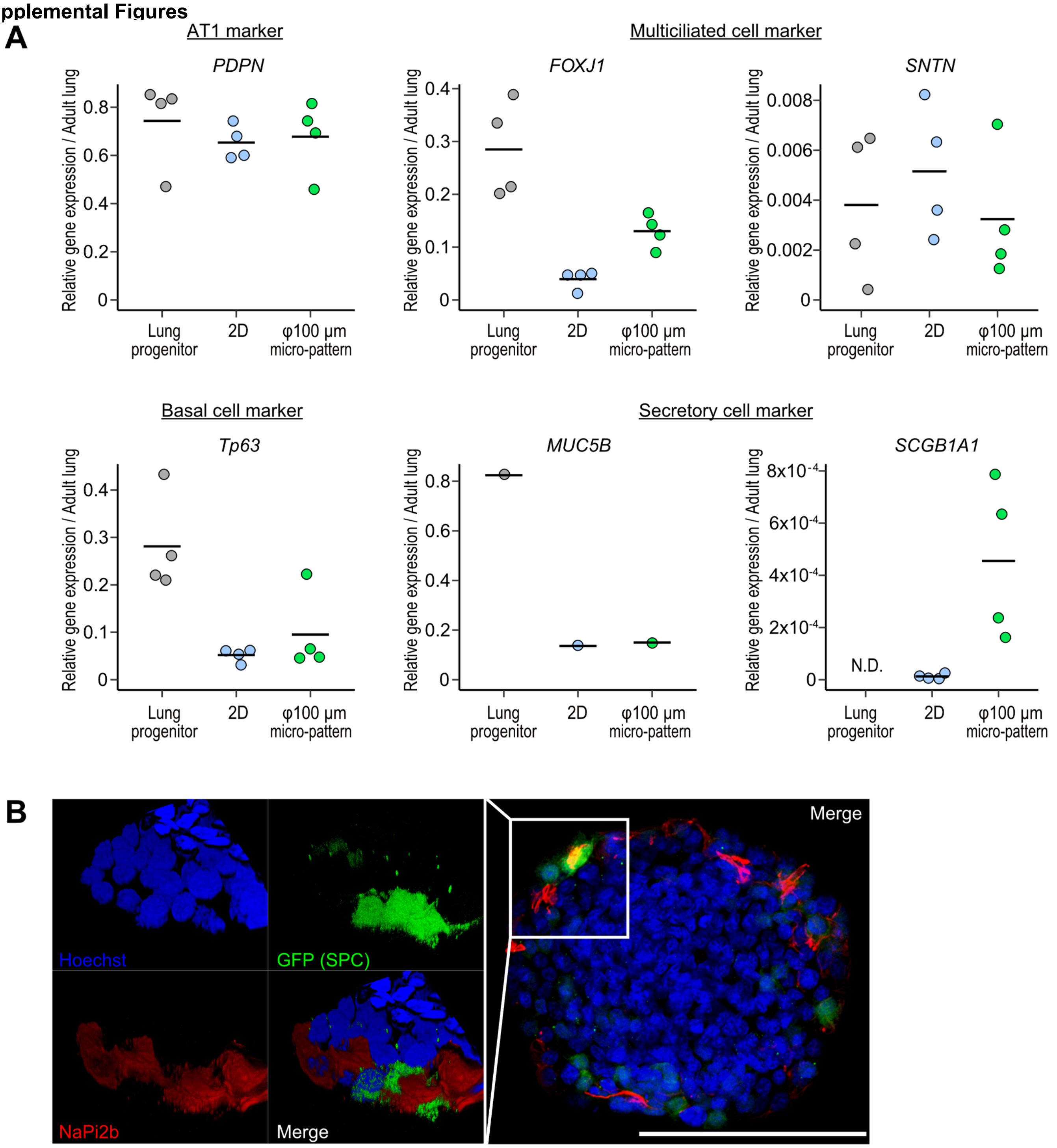

Figure S1. AT2s cultured in micro-patterned culture plates exhibit the apical side outward. Related to Figure 1.
A. Gene expressions of alveolar cells in micro-patterned or conventional 2D culture measured by qRT-PCR (n = 4 independent experiments).
B. 3D reconstructed imaging of alveolar epithelial cells cultured in micro-patterned plates. NaPi2B covers the surface of *SFTPC*^{GFP}-positive cells. Scale bar: 100 μm.

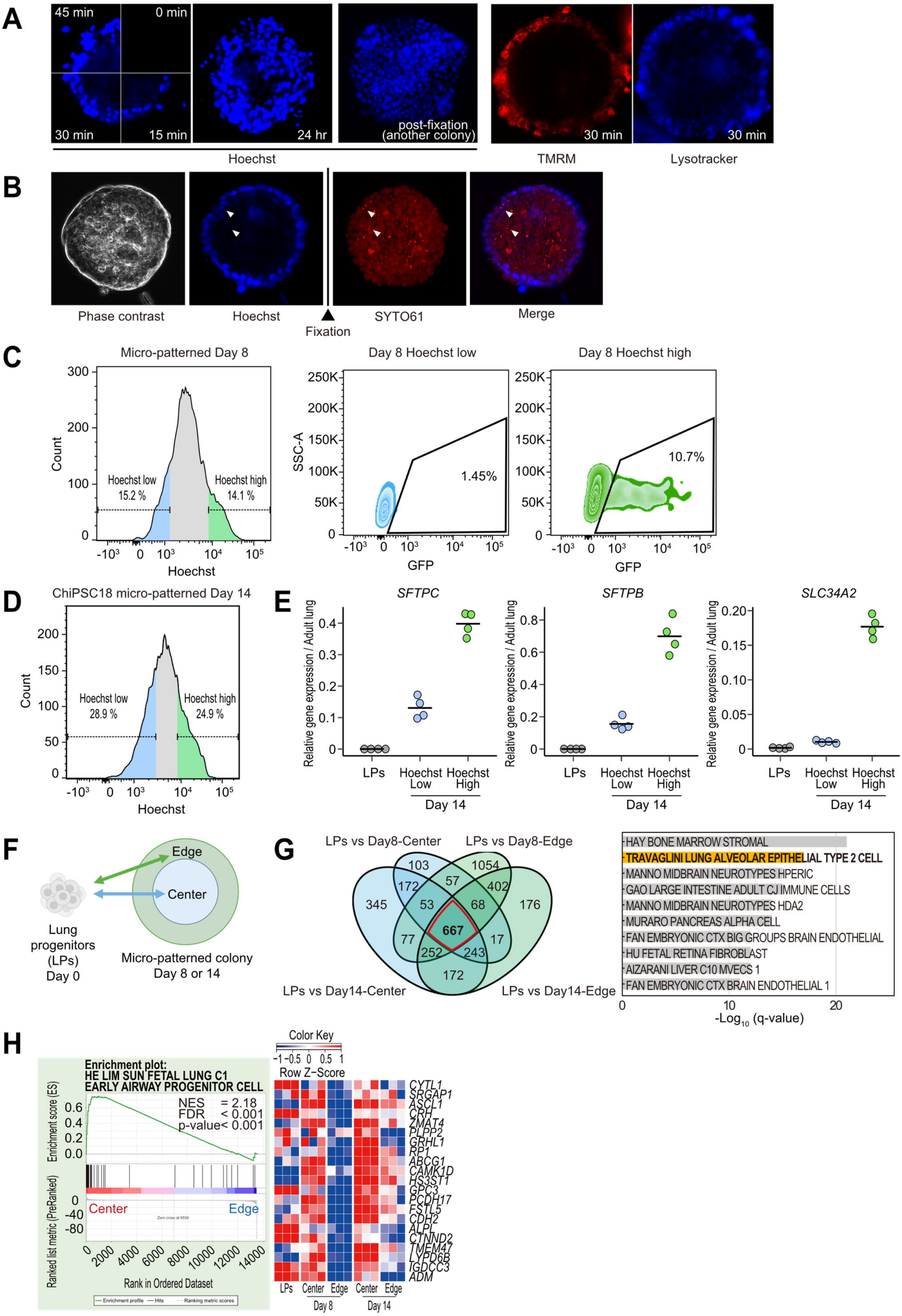
A
45 min
0 min
30 min
15 min
24 hr
post-fixation (another colony)
30 min
30 min
Hoechst
TMRM
Lysotracker
B
Phase contrast
Hoechst
Fixation
SYTO61
Merge
C
Micro-patterned Day 8
Count
Hoechst low 15.2 %
Hoechst high 14.1 %
Hoechst
Day 8 Hoechst low
SSC-A
1.45%
GFP
Day 8 Hoechst high
10.7%
GFP
D
ChiPSC18 micro-patterned Day 14
Count
Hoechst low 28.9 %
Hoechst high 24.9 %
Hoechst
E
SFTPC
SFTPB
SLC34A2
Relative gene expression / Adult lung
LPs
Hoechst Low
Hoechst High
Day 14
F
Edge
Center
Lung progenitors (LPs) Day 0
Micro-patterned colony Day 8 or 14
G
LPs vs Day8-Center
LPs vs Day8-Edge
LPs vs Day14-Center
LPs vs Day14-Edge
103
1054
172
57
402
345
53
68
176
667
77
17
252
243
172
HAY BONE MARROW STROMAL
TRAVAGLINI LUNG ALVEOLAR EPITHELIAL TYPE 2 CELL
MANNO MIDBRAIN NEUROTYPES HPERIC
GAO LARGE INTESTINE ADULT CJ IMMUNE CELLS
MANNO MIDBRAIN NEUROTYPES HDA2
MURARO PANCREAS ALPHA CELL
FAN EMBRYONIC CTX BIG GROUPS BRAIN ENDOTHELIAL
HU FETAL RETINA FIBROBLAST
AIZARANI LIVER C10 MVECS 1
FAN EMBRYONIC CTX BRAIN ENDOTHELIAL 1
-Log10 (q-value)
H
Enrichment plot:
HE LIM SUN FETAL LUNG C1 EARLY AIRWAY PROGENITOR CELL
NES = 2.18
FDR < 0.001
p-value< 0.001
Enrichment score (ES)
Ranked list metric (PreRanked)
Center
Edge
Rank in Ordered Dataset
Color Key
Row Z-Score
CYTL1
SRGAP1
ASCL1
CRH
ZMAT4
PLPP2
GRHL1
RP1
ABCG1
CAMK1D
HS3ST1
GPC3
PCDH17
FSTL5
CDH2
ALPL
CTNND2
TMEM47
LYPD6B
IGDCC3
ADM
LPs
Center
Edge
Center
Edge
Day 8
Day 14

Figure S2. Living AT2 colonies show high dye staining at the periphery for short periods. Related to Figure 2.

A. Live cell imaging of the Hoechst-, TMRM-, or Lysotracker-stained micro-patterned alveolar cell colonies. Cells in the post-fixation cells images were fixed in 4% PFA/PBS for 15 min and Hoechst-stained.

B. Images of live and fixed lung epithelial progenitor cells pattern-cultured for 8 days in DCIK+3i medium. Live cells were stained with Hoechst for 30 min, and phase contrast and fluorescence images were captured. The fixed cells were stained with SYTO61 for nuclear staining. Arrowheads indicate cells in mitosis.

C. Gating of Hoechst-Low or Hoechst-High cell populations at Day 8 in the micro-patterned culture and each rate of $SFTPC^{GFP}$ -positive cells.

D. A histogram of fluorescence intensity of Hoechst-stained cell colonies derived from ChiPSC18 iPSC in the micro-patterned culture analyzed using FACS. The rate of Hoechst-low cells was defined as the percentage equivalent to that of Hoechst-high cells on the opposite side of the histogram.

E. Representative gene expression levels of micro-patterned ChiPSC18 iPSC measured using qRT-PCR (n=4 independent experiments). AT2 marker genes were enriched in the Hoechst-high periphery of the cell colonies.

F. Comparison of differentially expressed genes (DEGs) between Edge and Center cell populations in micro-patterned cultures. The transcriptomes of the Edge and Center cells in the micro-patterned culture were compared with those of lung progenitors (LPs), and DEGs were defined as genes satisfying the following criteria: padj < 0.01, |log2 fold change| > 1.

G. 667 genes were identified as DEGs common across all time points and colony regions. Enrichment analysis was performed on these DEGs to determine cell type specificity.

H. GSEA analysis of "HE LIM Fetal lung C1 early airway progenitor cell" and heatmaps of their leading-edge genes that appear in the ranked list at or before the point at which the running sum reaches its maximum deviation from zero. Data from the micro-patterned Day 14 samples ranked by P-value comparing Center and Edge cell populations using DESeq2.

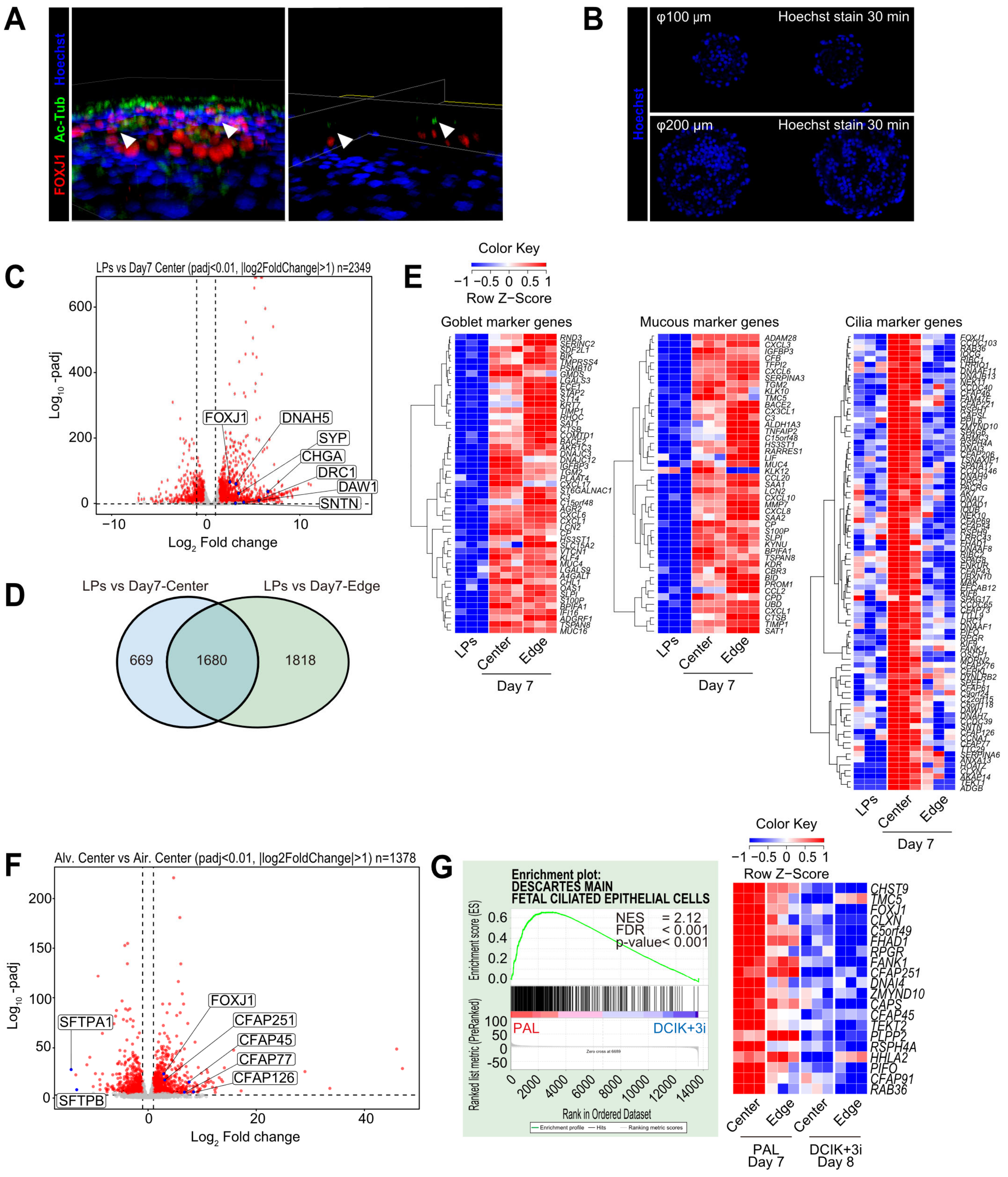
A
FOXJ1 Ac-Tub Hoechst
B
φ100 μm
Hoechst stain 30 min
φ200 μm
Hoechst stain 30 min
Hoechst
C
LPs vs Day7 Center (padj<0.01, |log2FoldChange|>1) n=2349
FOXJ1
DNAH5
SYP
CHGA
DRC1
DAW1
SNTN
Log10 -padj
Log2 Fold change
D
LPs vs Day7-Center
LPs vs Day7-Edge
669
1680
1818
E
Color Key
Row Z-Score
Goblet marker genes
Mucous marker genes
Cilia marker genes
LPs
Center
Edge
Day 7
F
Alv. Center vs Air. Center (padj<0.01, |log2FoldChange|>1) n=1378
SFTPA1
SFTPB
FOXJ1
CFAP251
CFAP45
CFAP77
CFAP126
Log10 -padj
Log2 Fold change
G
Enrichment plot:
DESCARTES MAIN
FETAL CILIATED EPITHELIAL CELLS
NES = 2.12
FDR < 0.001
p-value< 0.001
Enrichment score (ES)
Ranked list metric (PreRanked)
PAL
DCIK+3i
Rank in Ordered Dataset
Color Key
Row Z-Score
CHST9
TMC5
FOXJ1
CLXN
C5orf49
FHAD1
RPGR
FANK1
CFAP251
DNAI4
ZMYND10
CAPS
CFAP45
TEKT2
PLPP2
RSPH4A
HHLA2
PIFO
CFAP91
RAB36
Center
Edge
PAL
Day 7
DCIK+3i
Day 8

Figure S3. Multiciliated cells cultured in micro-patterned culture plates face the apical side outward. Related to Figure 3.

A. 3D reconstructed imaging of the airway epithelial cells cultured in the micro-patterned plates. Acetylated tubulin (Ac-Tub: arrowhead) is expressed on the colony surface.

B. Live cell imaging of the Hoechst-stained micro-patterned airway cell colonies on Day 14. The nuclei of the multiciliated cells in the center and those in the periphery of the colonies were strongly stained with Hoechst.

C. Volcano plot from DESeq2 analysis comparing LPs to Day 7 Center cell population (n=3 independent experiments).

D. Venn diagram of the two DEG groups. DEGs of LPs vs. Day 7 Center cells were compared with the other DEGs of LPs vs. Day 7 Edge cells.

E. Heatmaps presented with Z-scores of goblet, mucous, or cilia epithelial marker genes. Z-scores were calculated from log (TPM value) (n = 3 independent experiments).

F. Volcano plot from DESeq2 analysis to compare the center populations from DCIK+3i culture (Alv.) and PAL culture (Air.). (n = 3 independent experiments).

G. GSEA analysis of "Descartes Main Fetal Ciliated Epithelial Cells" and heatmaps of their leading top 20 -edge genes. Data from micro-patterned samples collected on Day 7 or 8 were used. These samples were ranked based on P-values obtained by comparing the central populations of Alv. cells and Air. cells using DESeq2.

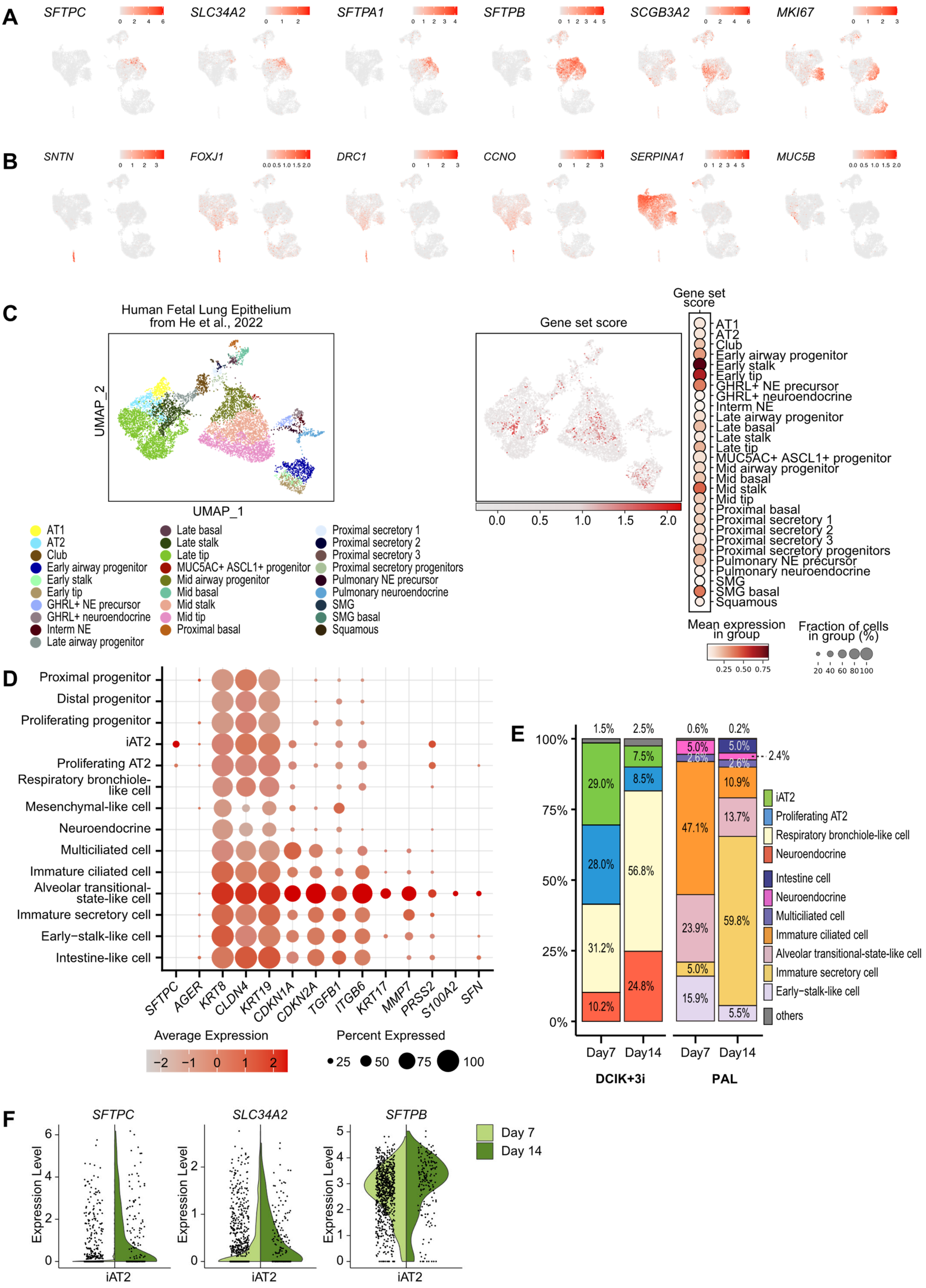

A
SFTPC
SLC34A2
SFTPA1
SFTPB
SCGB3A2
MKI67
B
SNTN
FOXJ1
DRC1
CCNO
SERPINA1
MUC5B
C
Human Fetal Lung Epithelium
from He et al., 2022
UMAP_2
UMAP_1
Gene set score
0.0 0.5 1.0 1.5 2.0
Gene set score
AT1
AT2
Club
Early airway progenitor
Early stalk
Early tip
GHRL+ NE precursor
GHRL+ neuroendocrine
Interm NE
Late airway progenitor
Late basal
Late stalk
Late tip
MUC5AC+ ASCL1+ progenitor
Mid airway progenitor
Mid basal
Mid stalk
Mid tip
Proximal basal
Proximal secretory 1
Proximal secretory 2
Proximal secretory 3
Proximal secretory progenitors
Pulmonary NE precursor
Pulmonary neuroendocrine
SMG
SMG basal
Squamous
Mean expression in group
0.25 0.50 0.75
Fraction of cells in group (%)
20 40 60 80 100
D
Proximal progenitor
Distal progenitor
Proliferating progenitor
iAT2
Proliferating AT2
Respiratory bronchiole-like cell
Mesenchymal-like cell
Neuroendocrine
Multiciliated cell
Immature ciliated cell
Alveolar transitional-state-like cell
Immature secretory cell
Early−stalk-like cell
Intestine-like cell
SFTPC AGER KRT8 CLDN4 KRT19 CDKN1A CDKN2A TGFB1 ITGB6 KRT17 MMP7 PRSS2 S100A2 SFN
Average Expression
−2 −1 0 1 2
Percent Expressed
25 50 75 100
E
100%
75%
50%
25%
0%
1.5% 2.5% 0.6% 0.2%
29.0% 28.0% 31.2% 10.2%
7.5% 8.5% 56.8% 24.8%
5.0% 2.6% 47.1% 23.9% 5.0% 15.9%
5.0% 2.6% 2.4% 10.9% 13.7% 59.8% 5.5%
Day7 Day14 Day7 Day14
DCIK+3i
PAL
iAT2
Proliferating AT2
Respiratory bronchiole-like cell
Neuroendocrine
Intestine cell
Neuroendocrine
Multiciliated cell
Immature ciliated cell
Alveolar transitional-state-like cell
Immature secretory cell
Early−stalk-like cell
others
F
SFTPC
SLC34A2
SFTPB
Expression Level
iAT2
Day 7
Day 14

Figure S4. Cellular composition and characteristics of lung epithelial cells in micro-patterned cultures. Related to Figure 4.

A and B. UMAP visualizations of gene expression patterns for iAT2-related (A) and airway epithelial-related (B) genes from single-cell RNA-seq data. Color intensity reflects expression levels.

C. UMAP visualization of human fetal lung epithelial cells derived from scRNA-Seq data in He et al., 2022. Left panel: color-coded by cell type; right panel: color-coded gene set score, calculated from 30 marker genes of the "Early-stalk-like cell" cluster identified in this study's scRNA-Seq analysis, with a Dot Plot accompanying it to the side.

D. Dot plot showing transitional AT2-related gene expressions in various cell types. X-axis presents selected genes; y-axis categorizes cell types. Dot size indicates the proportion of cells expressing each gene, and color intensity represents the average expression magnitude.

E. Stacked bar chart depicting the cell type composition on Day 7 and Day 14 for samples cultured in DCIK+3i (Alv.) and PAL media (Air.). Colored segments represent distinct cell types, with respective proportions displayed within each segment.

F. Violin plots overlaid with scatter plots depicting the expression of AT2-related genes in iAT2 cells on Day 7 (light green) and Day 14 (dark green). Violin plots represent data distribution, and scatter plots show individual expression levels at each time point.

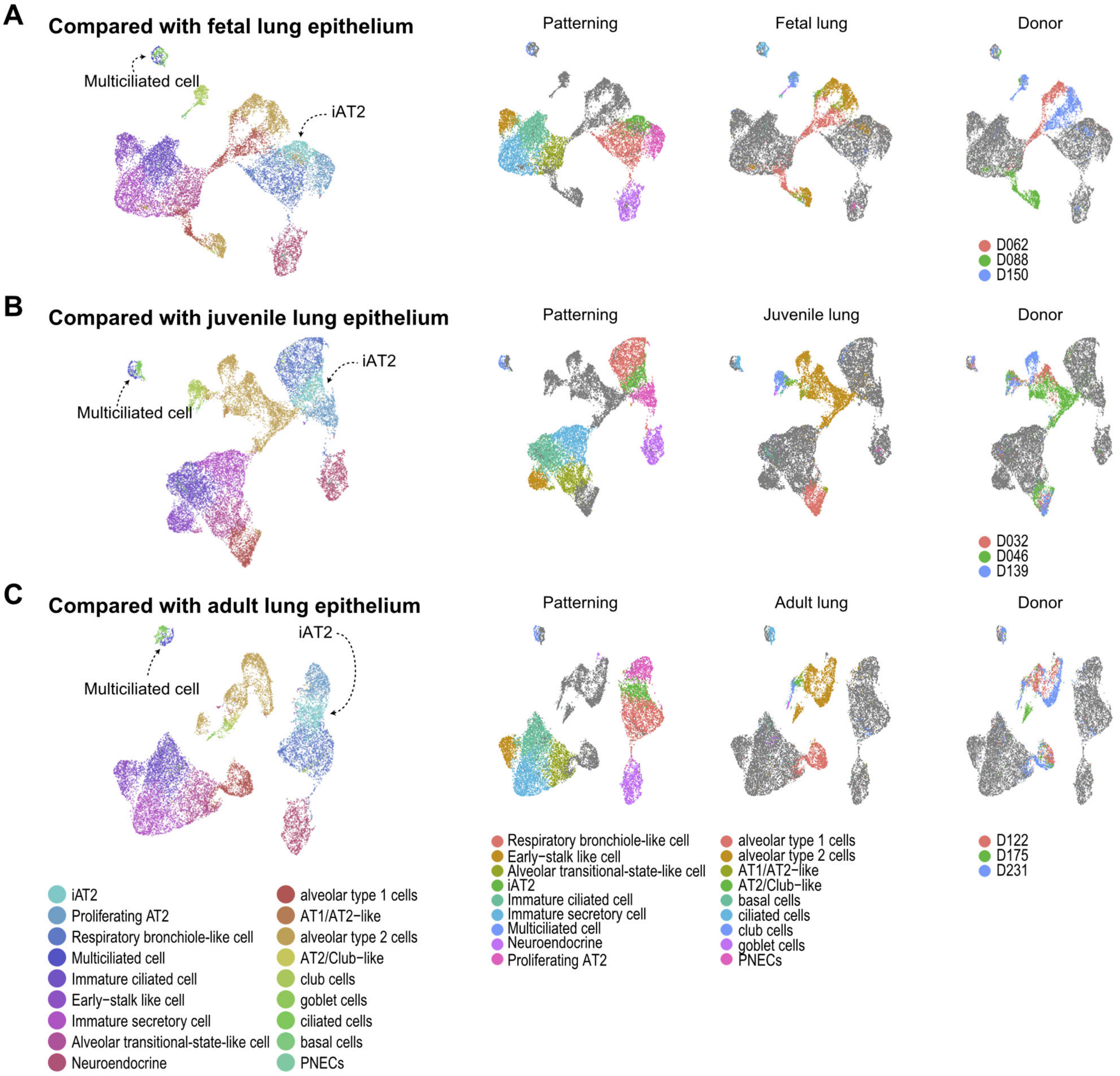

Figure S5. Overview of scRNA-seq transcriptomes of lung epithelial cells induced in micro-patterned culture in comparison with snRNA-Seq ones of primary lung epithelial cells (GSE161383). Related to Figure 4.

A, B, and C. UMAP visualization integrating scRNA-seq transcriptomes of the cells in the micro-patterned culture and snRNA-seq data from fetal (A), juvenile (B), and adult (C) cells, respectively. The annotated snRNA-seq data were obtained from GSE161383[1].

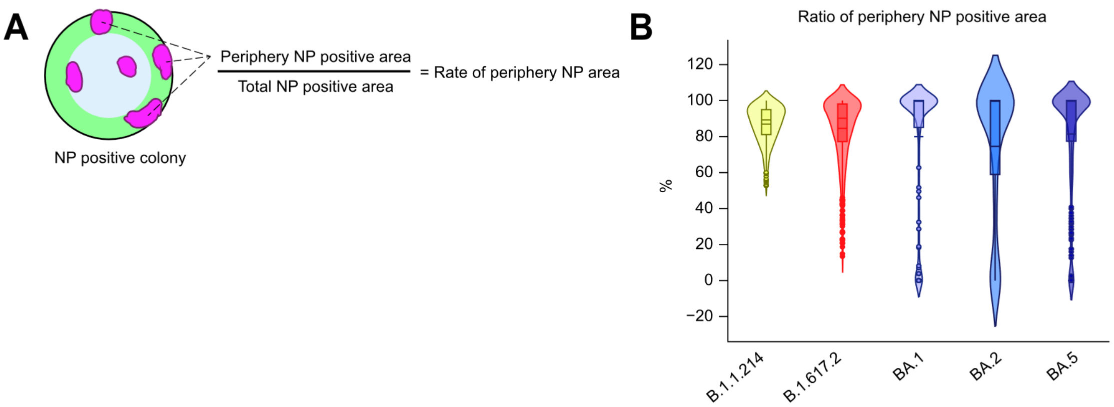

Figure S6. Quantification of the peripheral SARS-CoV-2 NP area ratio. Related to Figure 6.
A. Schematic diagram for quantifying the peripheral SARS-CoV-2 NP area ratio defined as the percentage of SARS-CoV-2 NP signal area in the periphery of the colonies. The total SARS-CoV-2 NP positive area was defined as the area of SARS-CoV-2 NP signals in the positive colonies.
B. Violin plot of the peripheral SARS-CoV-2 NP positive area ratio for alveolar epithelial cells infected with each variant. Measurements were performed by counting only SARS-CoV-2 NP-positive colonies. The number of colonies ranged from 105 to 974.

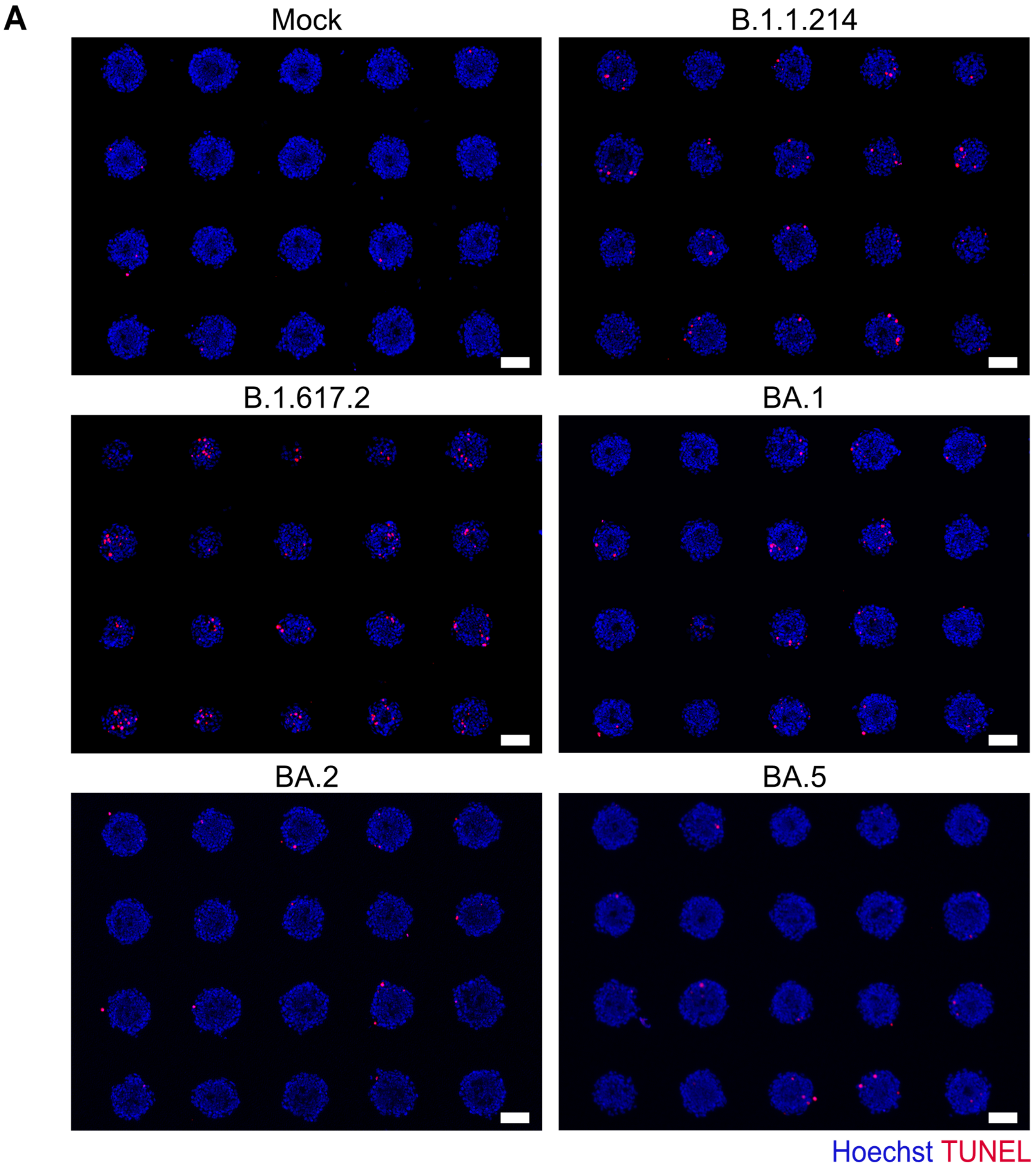

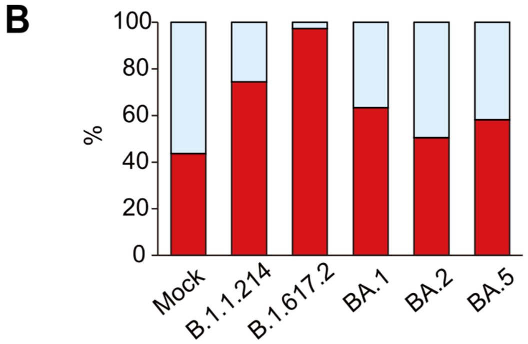

Variant	Total spot	TUNEL positive rate
Mock	1140	43.7 %
B.1.1.214	1169	74.4 %
B.1.617.2	1141	97.3 %
BA.1	1137	63.3 %
BA.2	1141	50.5 %
BA.5	1133	58.2 %

Figure S7. B.1.617.2 variant induces apoptosis of the airway epithelial cells. Related to Figure 7.
A. TUNEL staining images of the airway epithelial cells at 4 dpi with SARS-CoV-2 in the micro-patterned plate. Scale bar: 100 μm.
B. Ratio of TUNEL-positive iPSC-derived airway epithelial cell colonies infected with SARS-CoV-2 in micro-patterned culture. The number of colonies ranged from 1,133 to 1,169 in one representative experiment.

Supplemental Tables

Table S1. Genes are highly expressed in each colony region. Related to Figure 4.

DCIK+3i medium (Alv.)		PAL (Air.)	
**Center**	**Edge**	**Center**	**Edge**
CLDN5	*ICAM1*	*TMEM176B*	*TAGLN*
HES6	*WIF1*	*ASRGL1*	*SCEL*
HEPACAM2	*NMU*	*LIFR*	*VIM*
RGS4	*NRK*	*ST6GALNAC3*	*IGFBP4*
BTBD17	*FGG*	*SMOC2*	*COL4A2*
DLL3	*COL3A1*	*SOX21-AS1*	*FHL2*
SULF2	*PLAU*	*ELAPOR1*	*MMP7*
IGFBP5	*CDKN1A*	*MEIS1*	*GLIPR1*
RET	*CD55*	*LINC02381*	*ADAM8*
DLL1	*ME1*	*LRIG1*	*CYP1A1*
CRIP2	*CCNA2*	*DACT2*	*CD9*
CBFA2T2	*LAMC2*	*GSTA4*	*FSTL3*
BRINP2	*TK1*	*CFI*	*TNFRSF12A*
TFF3	*KIF23*	*PCDH19*	*LAMC2*
MIAT	*IL32*	*DMD*	*SH3BGRL3*
PRDM16	*SLPI*	*LINC00261*	*TM4SF1*
EGR2	*NSG1*	*TEKT2*	*AREG*
SUSD4	*PGC*	*SPON1*	*BCAT1*
ADM	*SLC34A2*	*SEMA6A*	*INHBA*
DAPK1	*DLC1*	*ELOVL2*	*CLDN7*
VCAN	*NAPSA*	*SCARA3*	*SFXN3*
NIBAN1	*GK*	*ZMYND10*	*IDS*
LRIG1	*SFTPC*	*CCNO*	*LAYN*
PGAP4	*TSPAN7*	*COL2A1*	*MFGE8*
GLB1L2	*ROS1*	*TPD52L1*	*AKAP12*
MYCL	*F3*	*LRAT*	*COL6A2*
ZFP36L2	*THBD*	*ASCL1*	*RAB11FIP1*
NPHP1	*ANKRD1*	*SERPINI1*	*KRT7*
SERPINF1	*EMP2*	*GPC3*	*TRIM16*
ADRA2A	*TUBB6*	*GSTA1*	*GAS6*

Table S2. Primers for Taqman qRT-PCR

Gene	Taqman ID
18S rRNA	Hs99999901_s1
AGER	Hs00153957_m1
MUC5B	Hs00861595_m1
SFTPC	Hs00161628_m1

Table S3. Primers for SYBR qRT-PCR

Gene	Forward primer sequence (5′–3′)	Reverse primer sequence (3′–5)
ACE2	ACAGTCCACACTTGCCCAAAT	TGAGAGCACTGAAGACCCATT
FOXJ1	CCTGTCGGCCATCTACAAGT	AGACAGGTTGTGGCGGATT
GAPDH	GGAGCGAGATCCCTCCAAAAT	GGCTGTTGTCATACTTCTCATGG
IFNA1	GCCTCGCCCTTTGCTTTACT	CTGTGGGTCTCAGGGAGATCA
IFNB1	ATGACCAACAAGTGTCTCCTCC	GGAATCCAAGCAAGTTGTAGCTC
MX1	CTTATCCGTTAGCCGTGGTG	CAAGGTGGAGCGATTCTGAG
PDPN	TCCAGGAACCAGCGAAGAC	CGTGGACTGTGCTTTCTGA
SARS-CoV-2	AGCCTCTTCTCGTTCCTCATCAC	CCGCCATTGCCAGCCATTC
SARS-CoV-2 N	CCAGGTAACAAACCAACCAACTTTCG	GGTTACTGCCAGTTGAATCTGAGG
SCGB1A1	CACCATGAAACTCGCTGTCAC	AGTTCCATGGCAGCCTCATAAC
SCGB3A2	CAAGTGGAACCACTGGCTTG	CCAGAGGTAAAGGTGCCAAC
SFTPB	GAGCCGATGACCTATGCCAAG	AGCAGCTTCAAGGGGAGGA
SLC34A2	TCGCCACTGTCATCAAGAAG	CTCTGTACGATGAAGGTCATGC

SNTN	GCTGCAAACCCAATTTAGGA	TGCTCATCAAGTTCAGAAAGGA
TMPRSS2	GTCCCCACTGTCTACGAGGT	CAGACGACGGGGTTGGAAG
TP63	ACTGCCAAATTGCAAAGACA	TGACTAGGAGGGGCAATCTG

Supplemental videos

Video S1. Human iPSC-derived LPs cultured in the micro-patterned plate for 14 days to achieve airway differentiation involving multiple cilia observed at the center of the colony, related to Figure 3.

Video S2. 3D reconstructed imaging of the alveolar epithelial cells cultured in the micro-patterned plate, related to Figure S1B.

SUPPLEMENTAL EXPERIMENTAL PROCEDURES

Human iPSC culture

All cells were cultured in 5% CO_2 at 37 °C. Human iPSCs were cultured as described previously [2]. Briefly, *SFTPC*^{GFP} reporter iPSC (B2-3) [3] and ChiPSC18 (Takara Bio, Y00300) were maintained in Essential 8 medium (Thermo Fisher Scientific, A1517001) on a 6-cm dish coated with Geltrex (Thermo Fisher Scientific, A1413201) and the media was changed daily. The cells were passaged at an appropriate split ratio (1:6–1:10) once they achieved approximately 80–90% confluency. Thereafter, 10 µm Y27632 was added to the medium for 24 h. Each iPSC line was grown to 100% confluence to differentiate into NKX2-1+ LPs. Both *SFTPC*^{GFP} reporter iPSC (B2-3) and ChiPSC18 were exempt from ethical approval.

Differentiation of human iPSCs into NKX2-1+ LPs

Human iPSCs were differentiated stepwise into lung progenitor cells (LPs), as previously described [2–4]. Briefly, human iPSCs were differentiated into definitive endoderm (DE) on Geltrex-coated plates in RPMI-1640 medium (Nacalai Tesque, 30264–56) containing 100 ng/mL activin A (API, GF-001-050L), 1 µM CHIR-99021 (Axon medchem, CT99021), 2% B27 supplement (Thermo Fisher Scientific, 17504–001), and 50 U/mL Penicillin/streptomycin (Thermo Fisher Scientific, 15140-122). Sodium butyrate (Fujifilm Wako, 193–01522) was added to a final concentration of 0.25 mM 24 h after seeding (Day 1). From days 2 to 6, the cells were cultured in RPMI-1640 medium containing 100 ng/mL activin A, 1 µM CHIR-99021, 2% B27 supplement, 50 U/mL Penicillin/streptomycin, and 0.125 mM sodium butyrate. In subsequent steps, DMEM/F12 (Thermo Fisher Scientific, 10565-042) medium supplemented with GlutaMAX, 2% B-27 supplement, 50 U/mL Penicillin/streptomycin, 0.05 mg/mL L-ascorbic acid (Fujifilm Wako, 016-04805), and 0.4 mM monothioglycerol (Fujifilm Wako, 195-15791) was used as basal medium. From days 6 to 10, DE cells were cultured in a medium supplemented with 100 ng/mL noggin (R&D systems, 6057-NG-01M) and 10 µM SB431542 (Fujifilm Wako, 198-16543) to differentiate into anterior foregut endoderm (AFE) cells. The AFE cells were cultured in the medium supplemented with 3-µM CHIR99021, cell-specific optimized concentrations of all-trans retinoic acid (B2-3: 0.05 µM, ChiPSC18: 1 µM) (Sigma-Aldrich, R2625), and 20 ng/mL BMP4 (Proteintech, HZ-1078) for days 10–14 to differentiate into ventralized anterior foregut endoderm (VAFE) cells. VAFE cells were efficiently distalized in the medium supplemented with 3 µM CHIR99021, 10 ng/mL FGF10 (Pepro Tech, 100-26), 10 ng/mL KGF (Pepro Tech, 100-19), and 20 µM DAPT (Fujifilm Wako, 049-33583) for 7 days. On days 20–22, NKX2-1+ LPs were isolated using an autoMACS Pro separator (Miltenyi Biotec) with mouse anti-human carboxypeptidase M (CPM) (Fujifilm Wako, 014-27501) and anti-mouse IgG microbeads (Miltenyi Biotech, 130-048-402).

SARS-CoV-2 preparation

SARS-CoV-2 strains B.1.1.214 (GISAID accession number: EPI_ISL_2897162), B.1.617.2 (EPI_ISL_9636792), BA.1 (EPI_ISL_9638489), BA.2 (EPI_ISL_11900505), and BA.5 (EPI_ISL_14018093) were isolated from the nasopharyngeal swab samples of patients with COVID-19[5]. This study was approved by the research ethics committee of Kyoto University (R2379-3). Viruses were produced in TMPRSS2/Vero cells (JCRB1818, JCRB Cell Bank) [6] and stored at -80 °C until use. TMPRSS2/Vero cells were cultured in minimum essential medium (MEM, Sigma-Aldrich, F0385-500ML) supplemented with 5% fetal bovine serum and 1% penicillin/streptomycin. All live virus experiments were performed in a biosafety level 3 facility at Kyoto University, following strict regulations.

Transmission electron microscopy

Cells were cultured for 14 days in DCIK+3i medium on micro-patterned culture plates. The cells were incubated in Accutase (Funakoshi, AT104-500) at 37 °C for 20 min and detached through gentle pipetting. The cell suspension was centrifuged at 200x *g* for 10 min at 4 °C. The cell pellet was embedded in Matrigel. The pellet-embedded Matrigel was incubated in a fixative solution comprising 2.5% glutaraldehyde (Nacalai Tesque, 17003-92), 4% paraformaldehyde (Nacalai Tesque, 26126-54), 0.1% picric acid, 4% sucrose (Nacalai Tesque, 30404-45) and 0.1 M phosphate buffer (pH 7.4) at 4 °C for 2 h, followed by incubation in 1% uranyl acetate en bloc at room temperature for 1 h[7]. The samples were washed in an ascending concentration of ethanol and propylene oxide and embedded in Epon 812. Thin sections were doubly stained with uranyl acetate and lead citrate and examined under a Hitachi H-7650 transmission electron microscope.

Live cell imaging
Phase-contrast and fluorescence images and movies were obtained using a BZ-X710 or BZ-X810 microscope (Keyence). Images were analyzed using a BZ-X or BZ-X800 analyzer, and the Image Cytometer Module was used to quantify the number of GFP-positive colonies and the percentage of GFP-positive areas.

Live cell fluorescence staining
The cells on the micro-pattern culture plates were incubated for up to 24 h under 5% CO_2 at 37 °C in a medium containing Hoechst-33342 (1:500), 20nM TMRM (Thermo Fisher Scientific, I34361), or 50nM LysoTracker Deep Red (Thermo Fisher Scientific, L12492). Fluorescence images were obtained using a BZ-X710 system.

Flow cytometry
Cell suspension and washing were performed using a flow cytometry buffer: PBS (1% BSA and 100 μM Y-27632). The cells were incubated in Accutase at 37 °C for 20 min and detached through gentle pipetting. The cells cultured on micro-pattern culture plates were first stained with Hoechst-33342 (1:500) for 30 min at 37°C, followed by incubation in Accutase at 37 °C for 20 min and dissociation through gentle pipetting. To define the Hoechst-high population, unstained cell suspensions were first dissociated using the same procedure, followed by staining with Hoechst (1:500) for 30 min at 37°C. All cell suspensions were washed and resuspended in flow cytometry buffer supplemented with PI solution (1:1000; Dojindo, 341-07881). Hoechst-low (center) and Hoechst-high (Edge) populations were separated according to the intensity of Hoechst using FACS Aria III (BD Biosciences). The Hoechst-high population was defined and compared with the fluorescence intensity of the Hoechst-stained sample in suspension. The Hoechst-low population was sorted from the opposite side of the peak with a similar percentage of cells to the Hoechst-high population. After isolation, the cells were centrifuged at 170x *g* for 10 min at 4 °C to remove the supernatant, lysed with the RLT buffer of the RNeasy Micro Kit (Qiagen, 74004) and stored at -80°C until total RNA extraction for RNA-seq or qRT-PCR analysis.

Immunofluorescence analysis
The cells on the micro-patterned culture plates were fixed in 4% paraformaldehyde/PBS heated to 37 °C and incubated at room temperature for 15 min. The fixed cells were stored at 4 °C in PBS until staining. The samples were permeabilized with 0.2% Triton X-100 (Nacalai Tesque, 12967-32)/PBS and blocked with a blocking buffer of PBS containing 5% normal donkey serum (EMD-Millipore, 566460) and 1% BSA (Sigma-Aldrich, A9647). Furthermore, the samples were stained overnight at 4 °C with the following primary antibodies diluted in the blocking buffer: chicken anti- GFP antibody (1:500, Aves Labs, #GFP-1020), mouse anti-NaPi2b (1:100, kindly provided by Dr. Gerd Ritter (MX35)), rabbit anti-SPB (1:500, Abcam, ab40876), mouse anti-FOXJ1 (1:500, R.T. 1hr, Thermo Fisher Scientific, 14-9965-82), mouse anti-Acetylated Tubulin (1:4000, R.T. overnight, Sigma-Aldrich, T7451), mouse anti-WIF1 (1:50, Santa Cruz Biotechnology sc-373780), rabbit anti-DLL3 (1:50, Cell Signaling Technology #71804) and rabbit anti-SARS-CoV-2 Nucleocapsid (1:200, SinoBiological 40588-T62). Subsequently, the samples were stained for 1 h at R.T. with Hoechst-33342 (1:1000, Dojindo H342) and the following secondary antibodies: AlexaFluor546-conjugated donkey anti-rabbit IgG (1:500, Thermo Fisher Scientific #A-10040), AlexaFluor647-conjugated donkey anti-rabbit IgG (1:500, Thermo Fisher Scientific #A-31573), AlexaFluor546-conjugated donkey anti-mouse IgG (1:500, Thermo Fisher Scientific #A-10036), and AlexaFluor488-conjugated donkey anti-chicken IgY (1:500, Jackson Immuno Research #703-485-155). Nuclear staining was performed by Hoechst-33342 or SYTO61 Red Fluorescent Nucleic Acid Stain (1 mM, Thermo Fisher Scientific S11343). TUNEL staining was performed using the DeadEnd Fluorometric TUNEL System (Promega, G3250). Staining was performed according to the manufacturer's instructions. A BZ-X710 or a TCS SP8 confocal microscope (Leica microsystems) was used for imaging.

Image analysis of the position-specific signals
The channel merge images captured using the Keyence all-in-one fluorescence microscope BZ-X710 were processed with Python 3.9.13. The center coordinates of the colonies were determined from the binarized merged images using the center_of_mass function from SciPy (version 1.11.4). For all pixels in each channel-separated image, the distance was calculated from the center coordinates and intensity. The intensity of pixels at equal distances from the center was averaged, and the data were sorted based on distance. Each channel underwent normalization, setting the maximum Intensity to 1 and the minimum Intensity to 0; the results were plotted as line graphs in the same plot area.

qRT-PCR
The PureLink RNA Mini Kit (Thermo Fisher Scientific, 12183025) or the RNeasy Micro Kit (Qiagen, 74004) was used to extract total RNA, depending on the experiment. Total RNA was reverse-transcribed using the ReverTra Ace qPCR RT Master Mix with gDNA Remover (TOYOBO, FSQ-301) according to the manufacturer's protocol. The qRT-PCR was performed on a StepOnePlus Real-Time PCR System using Power SYBR Green PCR Master Mix (Thermo Fisher Scientific, 4368577) or THUNDERBIRD Probe qPCR Mix (TOYOBO, QPS-101). Primers used in this study are listed in Table S2. Gene expression was normalized to eukaryotic 18S rRNA and compared with that of the human adult lung 5 donor pool (BioChain, #R1234152-P, lot A811037) or each control.

SARS-CoV-2 genome copy in culture supernatant quantification

The cell culture supernatant was mixed with an equal volume of 2×RNA lysis buffer (distilled water containing 0.4 U/μL SUPERase In™ RNase Inhibitor (Thermo Fisher Scientific, AM2694), 2% Triton X-100, 50 mM KCl, 100 mM Tris-HCl (pH 7.4), and 40% glycerol) and incubated at room temperature for 10 min. The mixture was diluted 10 times with distilled water. For quantifying SARS-CoV-2 RNA, the One-Step TB Green PrimeScript PLUS RT-PCR Kit (Perfect Real Time) (Takara Bio, RR096A) was used on a QuantStudio 1 or QuantStudio 3 real-time PCR system (Thermo Fisher Scientific). Standard curves were prepared using SARS-CoV-2 RNA (10^5 copies/μL) purchased from Nihon Gene Research Laboratories. The primer sequences are presented in Table S3.

Image analysis of SARS-CoV-2 infection samples

The cells grown on the micro-patterned culture plates were fixed in 4% PFA after treatment with SARS-CoV-2 infection and subjected to immunostaining for various marker proteins. Fluorescence images were captured for each well using a BZ-X810 with the Imaging Cytometer Module. The fluorescence intensity, number, and size in each colony were obtained from the images captured using a BZ-X800 Analyzer. The acquired data were processed using the KNIME Analytics Platform (Infocom). The sizes of the colonies and the areas of the various epithelial cell marker signals were normalized, and data with |Z-score| > 3 were excluded as outliers. The SARS-CoV-2 variant NP area in the colony was normalized for each infection group, and data with a |Z-score| > 3 were excluded as outliers. An average of 10.1% of the data were excluded from this process. The remaining data were used for the quantitative analyses.

qRT-PCR of SARS-CoV-2-infected cells

Total RNA was isolated from the infected cells at 4 dpi using ISOGEN (NIPPON GENE, 319-90211). cDNA was synthesized from 500 ng of total RNA using the Superscript VILO cDNA Synthesis Kit (Thermo Fisher Scientific, 11754050). Real-time RT-PCR was performed with the SYBR Green PCR Master Mix (Thermo Fisher Scientific, 4344463) using the StepOnePlus Real-Time PCR System, QuantStudio 1, or QuantStudio 3 Real-Time PCR System (Thermo Fisher Scientific). The relative quantification of target mRNA levels was performed using the $2^{-\Delta\Delta CT}$ method. Values were normalized to the housekeeping gene glyceraldehyde 3-phosphate dehydrogenase (GAPDH). The PCR primer sequences are shown in Tables S1-S2.

RNA-seq of the micro-patterned cells

Total RNA was extracted using the RNeasy Micro Kit according to the manufacturer's protocol. The RNA integrity of each sample was confirmed to be >8 using the 2100 BioAnalyzer. To analyze the Hoechst-high and Hoechst-low cell populations, 1 ng of the total RNA was reverse transcribed and amplified for 11 cycles using SMART-Seq HT (Clontech Laboratories, Z4456N). The amplified cDNA was used to prepare sequencing libraries with a Nextera XT DNA Library Preparation Kit (Illumina, FC-131). Library sequencing was performed using a NovaSeq 6000 or NextSeq2000 with the 100 bp paired-end method. Finally, raw data from this study were submitted to the Gene Expression Omnibus (GEO) under the accession number GSE236839 and GSE236840.

RNA-seq of the SARS-CoV-2-infected cells

Total RNA was isolated using the ISOGENE. The integrity of the RNA was assessed using the 2100 Bioanalyzer (Agilent Technologies). Similarly, library preparation was performed using the TruSeq Stranded mRNA Sample Prep Kit (Illumina, 20020594) according to the manufacturer's instructions. Furthermore, we performed sequencing using Illumina NextSeq550. We generated the FASTQ files using bcl2fastq-2.20. Adapter sequences and low-quality bases were trimmed from the raw reads using Cutadapt ver v3.4 [8]. Mapping of the trimmed reads to human reference genome sequences (hg38) was conducted using STAR version 2.7.9a [9] with a GENCODE (release 36, GRCh38.p13) GTF file [10]. We calculated the raw counts using the htseq-count ver. 0.13.5 [11] with the GENCODE GTF file. Gene expression levels were determined as transcripts per kilobase million (TPM) using DESeq2 v1.30.1 [12] . Finally, raw data from this study were submitted to the Gene Expression Omnibus (GEO) under the accession number GSE236841.

Bioinformatic analysis of RNA-seq data

Sequenced reads of the Hoechst-low (Center) or Hoechst-high (Edge) cells were trimmed using fastp [13], and the trimmed reads were aligned to GRCh38 using STAR 2.7.1a [9]. Transcript per million (TPM) values were calculated using RSEM [14]. Low expression genes with average TPM values among the comparison data set of less than 1 were excluded from downstream analyses. Principal component analysis of log2 (TPM+0.01) was performed using the R function "prcomp" and visualized using the R package "ggplot2." The R package DESeq2 [12] was used to identify DEGs. Volcano plots were visualized using the R package "ggplot2" and "ggrepel," and Venn diagrams for multiple DEGs were visualized using the R package "Venn Diagram." GSEA [15] was performed using genes ordered by P-values calculated using DESeq2. Enrichment analysis for GO based on biological processes was performed using the Metascape online software[16]. A heatmap using log2 (TPM+0.01) was visualized by the R package "gplots."

scRNA-seq of the micro-patterned colony cells

The cell suspensions were prepared via enzymatic dissociation; the micro-patterned cells were washed in PBS pre-warmed to 37 °C and immersed in 0.5 mM EDTA/PBS at 37 °C for 12 min. Subsequently, the cells were incubated with Accutase at 37 °C for 25 min and dissociated via gentle pipetting. LPs were isolated from the cell suspensions using MACS-based isolation of CPM-positive cells. Single-cell RNA libraries for lung epithelial cells and LPs were prepared using a 10X

Genomics Chromium device, according to the manufacturer's protocols specified in the Single Cell 3′ Reagent Kits v3.1. The 10X Genomics Cell Ranger pipeline (version 7.1.0) was used to perform sample demultiplexing, alignment to the hg38 human reference genome (refdata-gex-GRCh38-2020-A from 10X Genomics) and the reporter sequences, barcode/UMI processing, and gene counting for each cell. In the quality control (QC) of sequencing data, dead cells or outliers were identified and excluded based on two criteria: cells with fewer than 200 detected genes and cells with a high percentage of counts mapped to mitochondrial genes, with a threshold set at 15%. We used the Seurat package of the R software for data analysis and visualization. For comparative analysis of scRNA-seq with snRNA-seq, each transcriptome was integrated, and UMAP was drawn according to the 'anchor-based' integration workflow on the official Seurat website (https://satijalab.org/seurat/).

SUPPLEMENTAL REFERENCES

1. Wang, A., Chiou, J., Poirion, O.B., Buchanan, J., Valdez, M.J., Verheyden, J.M., Hou, X., Kudtarkar, P., Narendra, S., Newsome, J.M., et al. (2020). Single cell multiomic profiling of human lung reveals cell type-specific and age-dynamic control of SARS-CoV2 host genes. Elife *9*, 1–28. 10.7554/eLife.62522.

2. Yamamoto, Y., Gotoh, S., Korogi, Y., Seki, M., Konishi, S., Ikeo, S., Sone, N., Nagasaki, T., Matsumoto, H., Muro, S., et al. (2017). Long-term expansion of alveolar stem cells derived from human iPS cells in organoids. Nat. Methods *14*, 1097–1106. 10.1038/nmeth.4448.

3. Gotoh, S., Ito, I., Nagasaki, T., Yamamoto, Y., Konishi, S., Korogi, Y., Matsumoto, H., Muro, S., Hirai, T., Funato, M., et al. (2014). Generation of Alveolar Epithelial Spheroids via Isolated Progenitor Cells from Human Pluripotent Stem Cells. Stem Cell Reports *3*, 394–403. 10.1016/j.stemcr.2014.07.005.

4. Konishi, S., Gotoh, S., Tateishi, K., Yamamoto, Y., Korogi, Y., Nagasaki, T., Matsumoto, H., Muro, S., Hirai, T., Ito, I., et al. (2016). Directed Induction of Functional Multi-ciliated Cells in Proximal Airway Epithelial Spheroids from Human Pluripotent Stem Cells. Stem Cell Reports *6*, 18–25. 10.1016/j.stemcr.2015.11.010.

5. Hashimoto, R., Tamura, T., Watanabe, Y., Sakamoto, A., Yasuhara, N., Ito, H., Nakano, M., Fuse, H., Ohta, A., Noda, T., et al. (2023). Evaluation of Broad Anti-Coronavirus Activity of Autophagy-Related Compounds Using Human Airway Organoids. Mol. Pharm. *20*, 2276–2287. 10.1021/acs.molpharmaceut.3c00114.

6. Matsuyama, S., Nao, N., Shirato, K., Kawase, M., Saito, S., Takayama, I., Nagata, N., Sekizuka, T., Katoh, H., Kato, F., et al. (2020). Enhanced isolation of SARS-CoV-2 by TMPRSS2-expressing cells. Proc. Natl. Acad. Sci. U. S. A. *117*, 7001–7003. 10.1073/pnas.2002589117.

7. Osanai, K., Higuchi, J., Oikawa, R., Kobayashi, M., Tsuchihara, K., Iguchi, M., Huang, J., Voelker, D.R., and Toga, H. (2010). Altered lung surfactant system in a Rab38-deficient rat model of Hermansky-Pudlak syndrome. Am. J. Physiol. Lung Cell. Mol. Physiol. *298*, L243-51. 10.1152/ajplung.00242.2009.

8. Kechin, A., Boyarskikh, U., Kel, A., and Filipenko, M. (2017). cutPrimers: A New Tool for Accurate Cutting of Primers from Reads of Targeted Next Generation Sequencing. J. Comput. Biol. *24*, 1138–1143. 10.1089/cmb.2017.0096.

9. Dobin, A., Davis, C.A., Schlesinger, F., Drenkow, J., Zaleski, C., Jha, S., Batut, P., Chaisson, M., and Gingeras, T.R. (2013). STAR: ultrafast universal RNA-seq aligner. Bioinformatics *29*, 15–21. 10.1093/bioinformatics/bts635.

10. Frankish, A., Diekhans, M., Ferreira, A.-M., Johnson, R., Jungreis, I., Loveland, J., Mudge, J.M., Sisu, C., Wright, J., Armstrong, J., et al. (2019). GENCODE reference annotation for the human and mouse genomes. Nucleic Acids Res. *47*, D766–D773. 10.1093/nar/gky955.

11. Andrews, P.W., Barbaric, I., Benvenisty, N., Draper, J.S., Ludwig, T., Merkle, F.T., Sato, Y., Spits, C., Stacey, G.N., Wang, H., et al. (2022). The consequences of recurrent genetic and epigenetic variants in human pluripotent stem cells. Cell Stem Cell *29*, 1624–1636. 10.1016/j.stem.2022.11.006.

12. Love, M.I., Huber, W., and Anders, S. (2014). Moderated estimation of fold change and dispersion for RNA-seq data with DESeq2. Genome Biol. *15*, 550. 10.1186/s13059-014-0550-8.

13. Chen, S., Zhou, Y., Chen, Y., and Gu, J. (2018). fastp: an ultra-fast all-in-one FASTQ preprocessor. Bioinformatics *34*, i884–i890. 10.1093/bioinformatics/bty560.

14. Li, B., and Dewey, C.N. (2011). RSEM: accurate transcript quantification from RNA-Seq data with or without a reference genome. BMC Bioinformatics *12*, 323. 10.1186/1471-2105-12-323.

15. Subramanian, A., Tamayo, P., Mootha, V.K., Mukherjee, S., Ebert, B.L., Gillette, M.A., Paulovich, A., Pomeroy, S.L., Golub, T.R., Lander, E.S., et al. (2005). Gene set enrichment analysis: a knowledge-based approach for interpreting genome-wide expression profiles. Proc. Natl. Acad. Sci. U. S. A. *102*, 15545–15550. 10.1073/pnas.0506580102.

16. Zhou, Y., Zhou, B., Pache, L., Chang, M., Khodabakhshi, A.H., Tanaseichuk, O., Benner, C., and Chanda, S.K. (2019). Metascape provides a biologist-oriented resource for the analysis of systems-level datasets. Nat. Commun. *10*, 1523. 10.1038/s41467-019-09234-6.
