# Supplementary material for: Micro-patterned culture of iPSC-derived alveolar and airway cells distinguishes SARS-CoV-2 variants
Source: Stem Cell Reports. 2024 Mar 28;19(4):545–61. doi: 10.1016/j.stemcr.2024.02.011 (PMC11096626; doi:10.1016/j.stemcr.2024.02.011)
Supplement: Document S4. Article plus supplemental information [file mmc4.pdf]

Stem Cell Reports

Article

OPEN ACCESS

Micro-patterned culture of iPSC-derived alveolar and airway cells distinguishes SARS-CoV-2 variants

Atsushi Masui,[1,2] Rina Hashimoto,[1] Yasufumi Matsumura,[3] Takuya Yamamoto,[1,4,5] Miki Nagao,[3] Takeshi Noda,[6,7] Kazuo Takayama,[1,*] and Shimpei Gotoh[1,2,8,*]

[1]Center for iPS Cell Research and Application (CiRA), Kyoto University, Kyoto 606-8507, Japan
[2]Department of Drug Discovery for Lung Diseases, Graduate School of Medicine, Kyoto University, Kyoto 606-8501, Japan
[3]Department of Clinical Laboratory Medicine, Graduate School of Medicine, Kyoto University, Kyoto 606-8507, Japan
[4]Medical-risk Avoidance Based on iPS Cells Team, RIKEN Center for Advanced Intelligence Project (AIP), Kyoto 606-8507, Japan
[5]Institute for the Advanced Study of Human Biology (WPI-ASHBi), Kyoto University, Kyoto 606-8501, Japan
[6]Laboratory of Ultrastructural Virology, Institute for Life and Medical Sciences, Kyoto University, Kyoto 606-8507, Japan
[7]Laboratory of Ultrastructural Virology, Graduate School of Biostudies, Kyoto University, Kyoto 606-8507, Japan
[8]Lead contact
*Correspondence: kazuo.takayama@cira.kyoto-u.ac.jp (K.T.), gotoh.shimpei.5m@kyoto-u.ac.jp (S.G.)
https://doi.org/10.1016/j.stemcr.2024.02.011

SUMMARY

The emergence of severe acute respiratory syndrome-coronavirus-2 (SARS-CoV-2) variants necessitated a rapid evaluation system for their pathogenesis. Lung epithelial cells are their entry points; however, in addition to their limited source, the culture of human alveolar epithelial cells is especially complicated. Induced pluripotent stem cells (iPSCs) are an alternative source of human primary stem cells. Here, we report a model for distinguishing SARS-CoV-2 variants at high resolution, using separately induced iPSC-derived alveolar and airway cells in micro-patterned culture plates. The position-specific signals induced the apical-out alveolar type 2 and multiciliated airway cells at the periphery and center of the colonies, respectively. The infection studies in each lineage enabled profiling of the pathogenesis of SARS-CoV-2 variants: infection efficiency, tropism to alveolar and airway lineages, and their responses. These results indicate that this culture system is suitable for predicting the pathogenesis of emergent SARS-CoV-2 variants.

INTRODUCTION

Alveolar type 2 epithelial cells (AT2s) are essential in tissue repair during lung injuries, highlighting the need to study AT2s using *in vitro* culture systems. Because *in vitro* culture of primary AT2s is still challenging, induced pluripotent stem cells (iPSCs) are useful sources of AT2s. Human iPSC-derived AT2s (iAT2s) and other pulmonary epithelial cells are stepwise differentiated via NKX2-1^{+} lung progenitor cells (LPCs) and require three-dimensional (3D) culture methods with extracellular matrix (ECM) components such as Matrigel for stable iAT2 induction and expansion (Gotoh et al., 2014; Hawkins et al., 2017; Jacob et al., 2017; Yamamoto et al., 2017). However, Matrigel-based 3D culture complicates organoid size control and direct image analysis.

The severe acute respiratory syndrome-coronavirus-2 (SARS-CoV-2) pandemic prompted the urgent establishment of *in vitro* culture systems of pulmonary epithelial cells, including AT2s, for infection modeling and pathophysiological studies. Because SARS-CoV-2 is a respiratory virus that rapidly mutates and transforms its properties, *in vivo*-like cell culture systems should aid in evaluating concerning variants. However, Matrigel-embedded alveolar and airway organoids were inconvenient for modeling SARS-CoV-2 infection because their apical epithelial surface, hosting the major SARS-CoV-2 receptor (angiotensin-converting enzyme 2 [ACE2]), faces inward (Mulay et al., 2021; Tamai et al., 2022). In early studies, air-liquid interface (ALI) culture was suitable for exposing the apical surface of iAT2 to SARS-CoV-2 infection (Huang et al., 2020). However, it was difficult to clearly distinguish SARS-CoV-2 variants.

Micro-patterned culture technology enables manufacturing cells or organoids in defined sizes and shapes using the culture substrate or cell adhesion area. Recently, dome-like lung bud organoids, comprising various pulmonary cells derived from human embryonic stem cells (ESCs), were created using this method and subjected to SARS-CoV-2 infection (Rosado-Olivieri et al., 2023). However, identifying airway and alveolar cells within the 3D structure posed a challenge. We then separated the infection models of airway and alveolar epithelial cells into micro-patterned culture plates, thereby enabling a highly differentiated state of multiciliated airway cells and AT2s and quantitatively characterizing their infection by SARS-CoV-2 variants.

RESULTS

Micro-patterned culture induces apical-out AT2s derived from iPSCs

Micro-patterned culture plates consist of nonadhesive areas coated with a nonadhesive polymer and circular

© 2024 The Author(s).
This is an open access article under the CC BY license (http://creativecommons.org/licenses/by/4.0/).

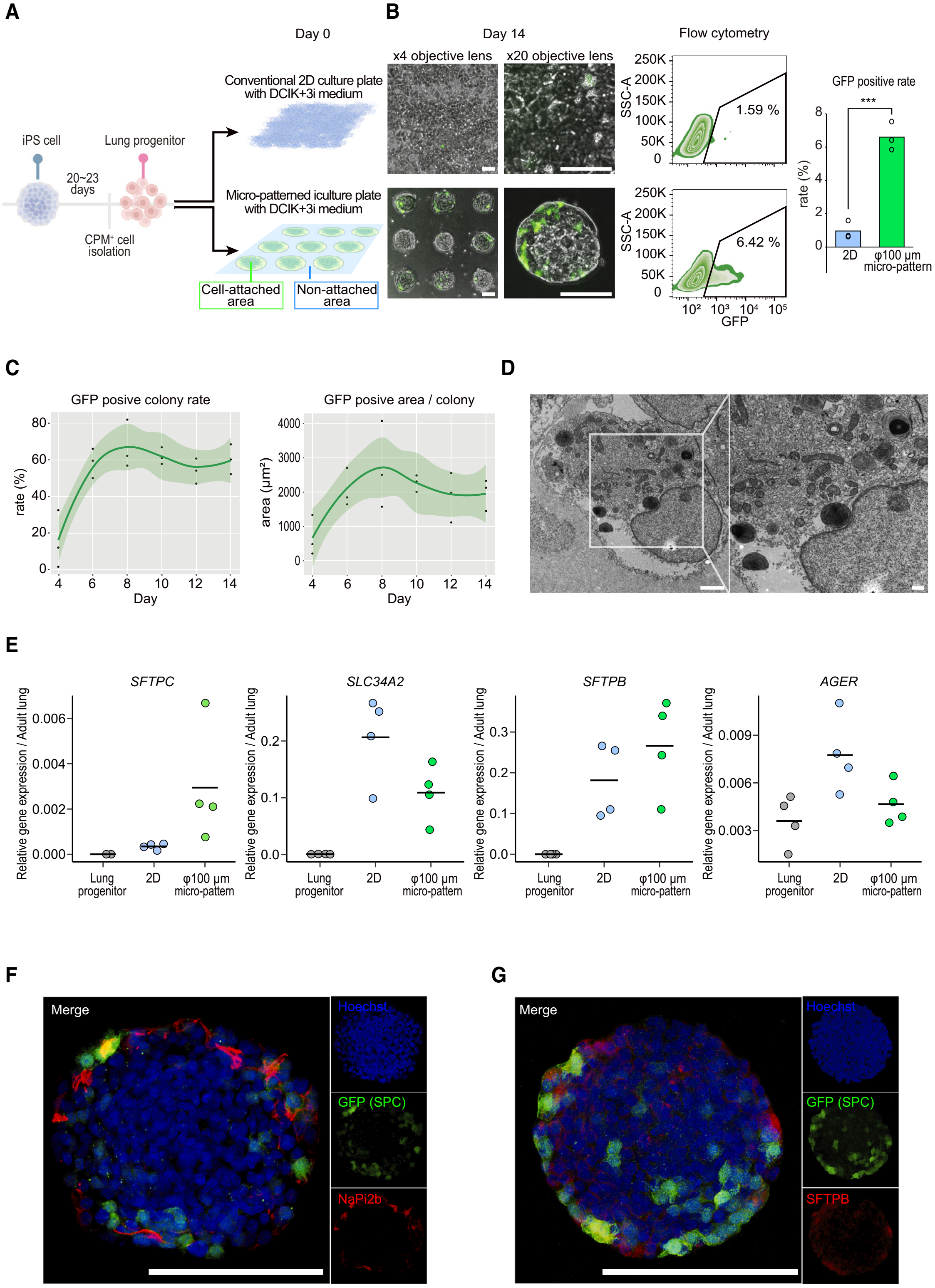

(legend on next page)

cell-adhesion areas of regularly aligned 100- or 200-μm diameter. We investigated whether human iPSC-AT2s could be induced in these plates. Carboxypeptidase M (CPM)$^+$ LPCs were induced stepwise from human *SFTPC*^{GFP} reporter iPSCs (B2-3) in which *SFTPC* is an AT2 marker (Gotoh et al., 2014), seeded onto these plates (2.5-dimensional culture plate, Tosoh, Japan), and maintained in the DCIK+3i medium, which we reported as fibroblast-free alveolarization medium (Yamamoto et al., 2017), resulting in the emergence of GFP$^+$ cells in the micro-patterned culture plates. Furthermore, we compared the number of GFP$^+$ cells in the micro-patterned culture plate with the conventional two-dimensional (2D) culture plate as a control after 14 days of culture (Figure 1A). Flow cytometry showed 6.6% ± 0.85% GFP$^+$ cells on the micro-patterned plate, compared to 0.97% ± 0.54% in the submerged condition on the conventional 2D culture plates (Figure 1B). We monitored the number of GFP$^+$ colonies and their area within colonies over time in micro-patterned cultures using image analysis (Figure 1C). GFP$^+$ cells emerged 6 days after seeding and were maintained for 2 weeks. Transmission electron microscopy showed lamellar bodies—restoring the pulmonary surfactant—(Figure 1D). These results suggest that AT2s can be induced from human iPSCs on a micro-patterned culture plate.

In quantitative reverse transcriptase-PCR (qRT-PCR), the *SFTPC* of the cells cultured in micro-patterned plates, unlike that in 2D cultures, increased compared with that of LPCs, consistent with *SFTPC*^{GFP} reporter expression (Figure 1E). *SLC34A2* and *SFTPB*—also AT2 marker genes—were upregulated in the 2D and micro-patterned cultures. The *AGER* expression—an AT1 marker gene—in cells in micro-patterned plates did not change compared with that in LPCs. We found no upregulation of any other pulmonary epithelial cell marker gene in the micro-patterned cells compared with LPCs (Figure S1A). These results suggest that LPCs cultured with DCIK+3i medium in micro-patterned plates differentiated into AT2 cells, not specifically into other pulmonary epithelial cells. In immunofluorescence analysis, GFP was localized to the periphery of each colony, consistent with the expression of NaPi2b, a phosphate transporter expressed on the apical surface of AT2s, and SFTPB (Figures 1F and 1G). Confocal microscopy revealed that NaPi2b was expressed toward the outside of colonies (Figure S1B). These results indicate that the iPSC-AT2s induced in the micro-patterned plates were localized to the periphery of each colony, where their apical surface expressing NaPi2b faced outward.

Region-specific program promotes the induction of iPSC-AT2s within the peripheral region of micro-patterned LPC colonies

We found that Hoechst33342 quickly stained live cells at the periphery of the colony. We isolated the periphery and central cell fractions from micro-patterned culture plates on days 8 or 14 using this phenomenon and counted the rate of GFP$^+$ cells. The peripheral cells exhibited intense Hoechst33342 staining for up to 45 min (Figures 2A and S2A), in contrast to the central region's lower intensity. The staining differences disappeared for 24 h of exposure to Hoechst33342 or staining after fixation with 4% paraformaldehyde (Figure S2A). After confirming the live-cell Hoechst staining, the cells were fixed and nuclei were stained with SYTO61. The DNA quantity determined based on the fluorescence intensity of SYTO61 did not correlate with Hoechst staining (Figure S2B). Subsequently, we collected the peripheral and central cells of the colonies via fluorescence-activated cell sorting (FACS) based on differences in Hoechst intensity (Figure 2B). The ratios of Hoechst high- and low-stained cells were 15.53% ± 1.23% and 16.80% ± 1.16% of the total cells on day 8, and 11.6% ± 0.96% and 11.9% ± 1.47% of the total cells on day 14, respectively (Figures 2C and S2B). On days 8 and 14, the percentage of GFP$^+$ cells was significantly higher in the Hoechst high-stained cells from the colony periphery than in low-stained cells from the center (Figures 2D and 2E). This indicated that iPSC-AT2s in the micro-patterned plates were localized to the periphery of the colonies.

Figure 1. Micro-patterned culture of human iPSC-derived alveolar epithelial cells

(A) Schematic diagram of human iPSC-derived alveolar epithelial cell development in a micro-patterned culture plate. The *SFTPC*^{GFP} reporter iPSC-derived LPCs sorted using anti-CPM IgG and magnetic beads were seeded onto the micro-patterned or conventional 2D culture plates (day 0) and cultured for 14 days.

(B) Live-cell imaging of micro-patterned or conventional 2D culture at day 14 and quantification of the GFP$^+$ cell rate. Data are means (n = 3 independent experiments). Unpaired 2-tailed Student's t test; ***p < 0.001. Scale bar: 100 μm.

(C) Local regression plots of the rate of GFP$^+$ colonies and GFP$^+$ area within colonies (n = 3 independent experiments).

(D) Transmission electron microscopy imaging of the alveolar epithelial cells in micro-patterned culture on day 14. Scale bars: 2 μm (left) and 500 nm (right).

(E) Representative gene expression of human iPSC-derived alveolar epithelial cells in micro-patterned or conventional 2D culture analyzed using qRT-PCR (n = 4 independent experiments).

(F and G) Immunofluorescent images of human iPSC-derived alveolar epithelial cells cultured on micro-patterned plates for 14 days. Double immunostaining of GFP with NaPi2b (F) and SFTPB (G) are shown. Scale bar: 100 μm.

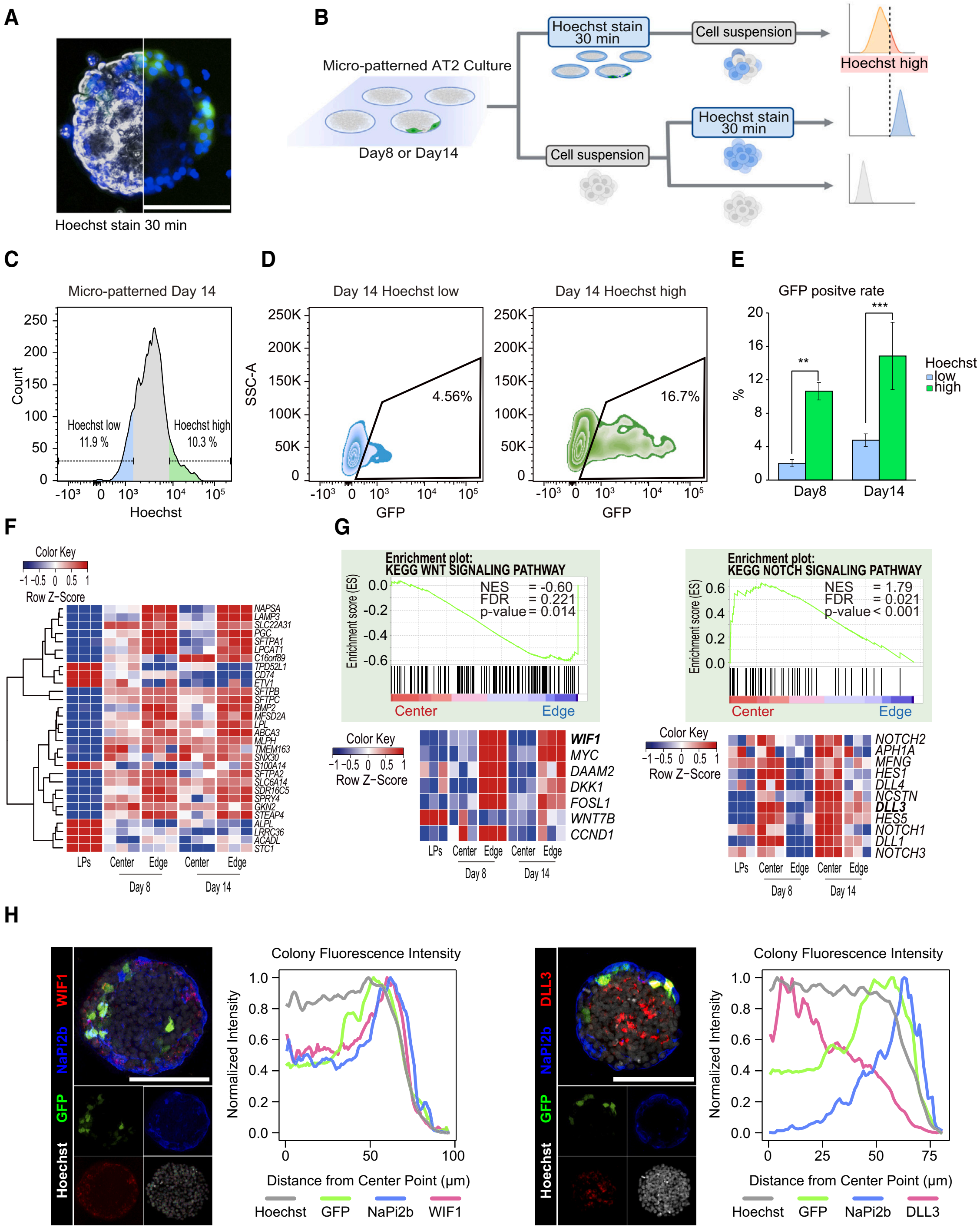

Figure 2. Alveolar epithelial cells are preferentially induced at the edge of the colonies in micro-patterned culture

(A) Live-cell imaging of the Hoechst33342-stained colonies in the micro-patterned culture. Scale bar: 100 μm.

(B) Experimental strategy for Hoechst-based separation of human iPSC-derived alveolar epithelial cells. Micro-patterned colonies were stained with Hoechst for 30 min and dissociated. Hoechst high and Hoechst low cells were isolated using FACS (top). For Hoechst high gating, the micro-patterned cells were dissociated into single cells, stained with Hoechst for 30 min, and analyzed using flow cytometry (center). Unstained cells were used as a negative control (bottom).

(C) Histogram of fluorescence intensity of Hoeschst-stained micro-patterned culture cells analyzed using FACS. The rate of Hoechst low cells was defined as the percentage equivalent to that of Hoechst high cells on the opposite side of the histogram.

(legend continued on next page)

To verify the high Hoechst staining intensity of iPSC-AT2s in the peripheral cells of the colonies not derived from the $SFTPC^{GFP}$ reporter cell line, we performed the same experiment using ChiPSC18, derived from another healthy donor (Figures S2C and S2D). After 2 weeks in micro-patterned culture, the periphery of the ChiPSC18 cell colony exhibited high Hoechst staining, like that of $SFTPC^{GFP}$ reporter cells (Figure S2D). In addition, following recovery of the peripheral and central cells, significant enrichment of the AT2 marker gene was observed in Hoechst high cells (Figure S2E). Therefore, the periphery of the colonies in the micro-patterned culture was suitable for generating iPSC-AT2s from their LPCs.

We hypothesized that position-specific signals in each colony caused LPCs to differentiate into AT2s in the micro-patterned culture. To test this hypothesis, we performed RNA sequencing (RNA-seq) on day 0 LPCs and Hoechst low (Center) and Hoechst high (Edge) cells in the micro-patterned colonies on days 8 and 14 (Figure 2F). The differentially expressed genes (DEGs) between LPCs and day 14 Edge cells were compared, and 1,997 DEGs were identified (Figure S2F). AT2 marker genes (*SFTPC*, *SFTPB*, *ABCA3*, *SLC34A2*, and *NAPSA*) were significantly upregulated (Figure 2F). Although AT2 signatures were enriched in the 667 DEGs across all time points and cell positions (Figures S2G and S2H), they were markedly higher in the peripheral cells of the colonies in the micro-patterned culture. Furthermore, gene set enrichment analysis (GSEA) on day 14 revealed WNT signal enrichment in Edge cells (nominal $p = 0.014$, false discovery rate [FDR] $q = 0.221$) (Figure 2G). More specifically, *WIF1* and *DKK1*, the canonical WNT repressors, were upregulated in Edge cells. This suggests that canonical WNT signaling is suppressed in the peripheral regions of the colonies. In contrast, Notch signaling was activated in Center cells (nominal $p < 0.001$, FDR $q = 0.021$) (Figure 2G). Next, we performed fluorescent immunostaining to visualize the positional signals identified via RNA-seq. Image analysis revealed that WIF1 was localized to the periphery of the colonies. In contrast, DLL3, a NOTCH ligand, was localized in the center of the colonies (Figure 2H). Although Notch signaling is reportedly essential for airway and alveolar epithelial cell differentiation (Rock et al., 2011), GSEA detected enrichment of the early airway progenitor cell signature in Center cells (Figure S2H). Accordingly, we hypothesized that the central region of the colonies is suitable for airway epithelial cell differentiation, considering the position-specific preference for alveolar epithelial cell differentiation.

Airway epithelial cells are differentiated in the central region of the micro-patterned colonies

We investigated whether airway epithelial cells differentiated in the micro-patterned culture (Figure 3A). In DCIK+3i medium, the transcriptome patterns resembled those of airway epithelial progenitor cells; however, multiciliated airway epithelial cells were not detected. Therefore, to promote their differentiation toward mature airway epithelial cells, LPCs seeded on the micro-patterned plates were cultured in PAL medium, which we previously reported useful for inducing multiciliated epithelial cells (Konishi et al., 2016). We observed moving cilia in the center of the colonies at approximately day 10 (Video S1). qRT-PCR revealed upregulations of *FOXJ1* and *SNTN*, multiciliated cell markers, on day 14. Their expression was higher in the 3×10^5 cells/well condition with a spot diameter of φ200 μm than in the other conditions (Figure 3B). This could be because the colony center with ciliated epithelium was larger in the spot with φ200 μm than φ100 μm. We performed the subsequent experiments using a plate with φ100 μm spots to match the alveolar epithelial cell cultures. Other pulmonary epithelial cell marker genes showed unstable induction or low expression (Figure 3B). $FOXJ1^+$ and acetylated $tubulin^+$ multiciliated cells were identified in the colony center using immunofluorescence staining (Figures 3C and S3A). Acetylated tubulin localized to the cell apices in the colony center (Figure S3A), indicating outward-facing airway epithelial cells. Furthermore, we quantified the percentage of $FOXJ1^+$ cells using image analysis, revealing a higher percentage in micro-patterned cultures compared to the conventional 2D culture, where

(D and E) Rates of GFP^+ cells in Hoechst low or Hoechst high cells (D). Data are mean ± SEM (n = 3 independent experiments) (E). Two-way ANOVA multiple comparisons: $^{**}p < 0.01$; $^{***}p < 0.001$.
(F) Comparison of DEGs between the edge and center cell populations in the micro-patterned cultures. The transcriptomes of the edge and center cells in the micro-patterned culture were compared with those of LPCs, and DEGs were defined as genes satisfying the following criteria: padj <0.01, |log2 fold change| > 1. The heatmap presents the *Z* scores for AT2-related genes, calculated from log (transcripts per kilobase million [TPM] value) (n = 3 independent experiments).
(G) GSEA analyses of the KEGG (Kyoto Encyclopedia of Genes and Genomes) WNT signaling pathway and the KEGG NOTCH signaling pathway, with each heatmap of the leading genes in the Edge cell population. Data from day 14 micro-patterned samples were ranked using p values comparing the Center and Edge cell populations using DESeq2.
(H) Immunofluorescence image analyses showing position-specific signals. Line graphs illustrate the relationship between the distance from the colony center and the fluorescence intensity, representing average values from 5 colonies in 1 representative experiment. Scale bar: 100 μm.

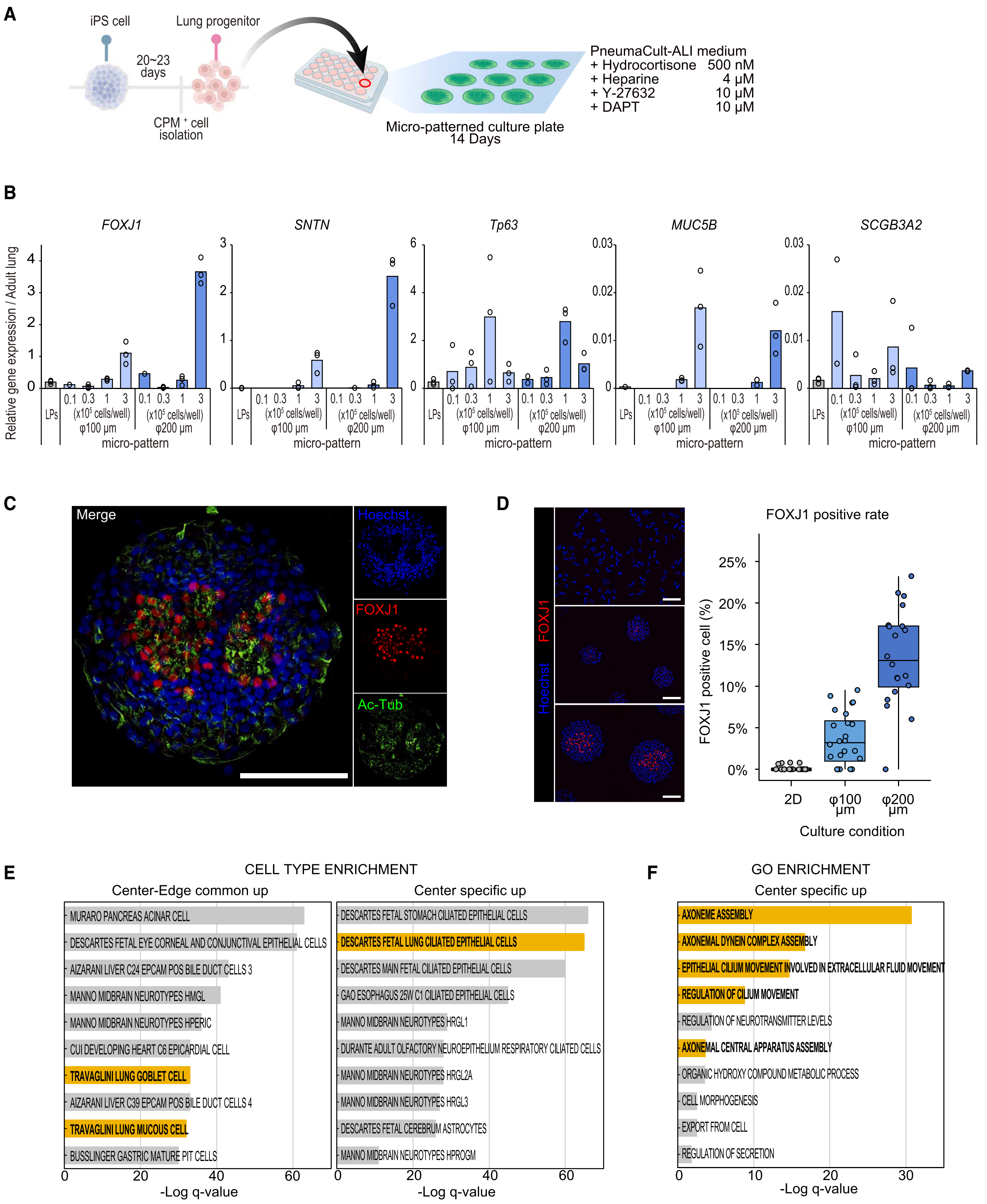

(legend on next page)

the percentage of FOXJ1-positive cells was markedly lower in the submerged condition (Figure 3D).

Subsequently, we analyzed isolated Center and Edge cells via RNA-seq, as described for alveolar epithelial cells. Considering that multiciliated cells in the center of the colonies on day 14 exhibited high Hoechst intensity (Figure S3B), separating the Center and Edge cells using Hoechst intensity was challenging at this time point. Therefore, we isolated the Center and Edge cells on day 7, before multiciliated cells emerged, and compared the transcriptomes of LPCs on day 0 with those of Center and Edge cells in the micro-patterned colonies. Several marker genes for multiciliated and neuroendocrine cells were upregulated within the colony center on day 7 compared with the LPCs (Figure S3C). We identified 669 genes as Center-specific DEGs, 1,818 as Edge-specific DEGs, and 1,680 as common to Center and Edge cells (Figure S3D). The upregulated genes common to the Center and Edge cells enriched the goblet and mucous cell lineages. The Gene Ontology (GO) term for lung-ciliated epithelial cells was enriched in the upregulated center-specific DEGs (Figures 3E and S3E). Furthermore, GO enrichment analysis of Center-specific DEGs revealed multiple enriched terms associated with cilia (Figure 3F). Next, we compared the transcriptomes of Center cells cultured in the DCIK+3i and PAL media. A total of 1,378 DEGs were identified. Center cells in PAL medium showed significantly upregulated multiciliated cell-related genes and downregulation of AT2-related genes compared to DCIK+3i medium (Figure S3F). GSEA also identified "fetal ciliated epithelial cells" marker genes enrichment (nominal $p < 0.001$, FDR $q < 0.001$) (Figure S3G). These results suggest that multiciliated airway epithelial cells appear in the colony center, providing a suitable microenvironment for airway epithelial cell differentiation.

Single-cell RNA-seq (scRNA-seq) analysis of the micro-patterned colonies of alveolar and airway epithelial cells

To characterize the specific cell types that make up the micro-patterned colonies, scRNA-seq was performed at each stage of CPM$^+$ progenitor cell differentiation in DCIK+3i and PAL media on days 7 and 14 (Figure 4A). The analysis revealed distinct transcriptomes among the progenitor and micro-patterned cells in each medium (Figure 4B). Within the CPM$^+$ progenitor cells, three subpopulations were identified, each predominantly expressing *SOX2*, *SOX9*, or *MKI67* (Figures 4C and 4D). These findings were consistent with the potential of CPM$^+$ progenitor cells to differentiate into alveolar and airway epithelial lineages. The cell population cultured in DCIK+3i medium mainly comprised three clusters: (1) iAT2 cluster characterized by high expression of AT2 marker genes (*SFTPC*, *SLC34A2*, *SFTPA1*, and *SFTPB*); (2) proliferating AT2 cluster with low AT2 marker expression and positive for MKI67; and (3) respiratory bronchiole-like cell expressing *SFTPB* and *SCGB3A2* (Figure S4A) (Basil et al., 2022; Kadur Lakshminarasimha Murthy et al., 2022). The third cluster is suggested to represent cells located in the central region of the colonies by the high expression of genes characteristic of central cells (Table S1), as observed in the bulk RNA-seq results (Figure 4E). The cells cultured in PAL medium contained a multiciliated cluster expressing markers, such as *SNTN* and *FOXJ1* (Figure 4C). The remaining clusters, still immature, expressed genes associated with ciliated epithelial cells (*FOXJ1*, *DRC1*, and *CCNO*) or immature secretory cells (*SERPINA1* and *MUC5B*) (Figure S4B). In addition, stalk cell-like clusters, similar to those described by He et al. (2022), were observed, indicating the presence of cells in an earlier developmental stage compared to the previously described clusters (Figure S4C). Cells in the peripheral region formed an independent cluster that interestingly resembled the transcriptome of the intermediate cells differentiated from AT2s to AT1s (Figure S4D). This cluster was annotated as alveolar transitional state-like cell, previously reported in the literature (Kobayashi et al., 2020). Neuroendocrine cells were detected in DCIK+3i and PAL cultures. Mesenchymal-like and intestinal epithelial-like cells were rare in these cultures (Figures 4D and S4E). We assessed the similarity of scRNA-seq transcriptomes data of iAT2 and multiciliated cells induced in micro-patterned

Figure 3. Airway epithelial cells are induced at the center of the colonies in micro-patterned culture

(A) Schematic diagram of generating human iPSC-derived airway epithelial cells in a micro-patterned culture plate. LPCs were differentiated into airway epithelial cells on a micro-patterned plate for 14 days.

(B) Representative gene expression of human iPSC-derived airway epithelial cells in the micro-patterned culture analyzed using qRT-PCR (n = 3 independent experiments). Data are presented as mean values.

(C) Immunofluorescence imaging of the airway epithelial cells in the micro-patterned culture for 14 days. Scale bar: 100 μm.

(D) Immunofluorescence imaging for quantifying the percentage of FOXJ1$^+$ multiciliated cells (top: 2D culture, center: patterned culture φ100 μm, bottom: patterned culture φ200 μm). The boxplot shows the percentage of FOXJ1$^+$ cells. Each dot represents the percentage of FOXJ1$^+$ cells per field of view (2D) or per colony (micro-patterned culture) from 1 representative experiment. n = 20–24. Scale bar: 100 μm.

(E) Cell-type enrichment analysis of the commonly upregulated genes in Center and Edge cells (1,003 genes) and the Center-specific upregulated DEGs (368 genes).

(F) GO enrichment analysis of the Center-specific upregulated DEGs (368 genes).

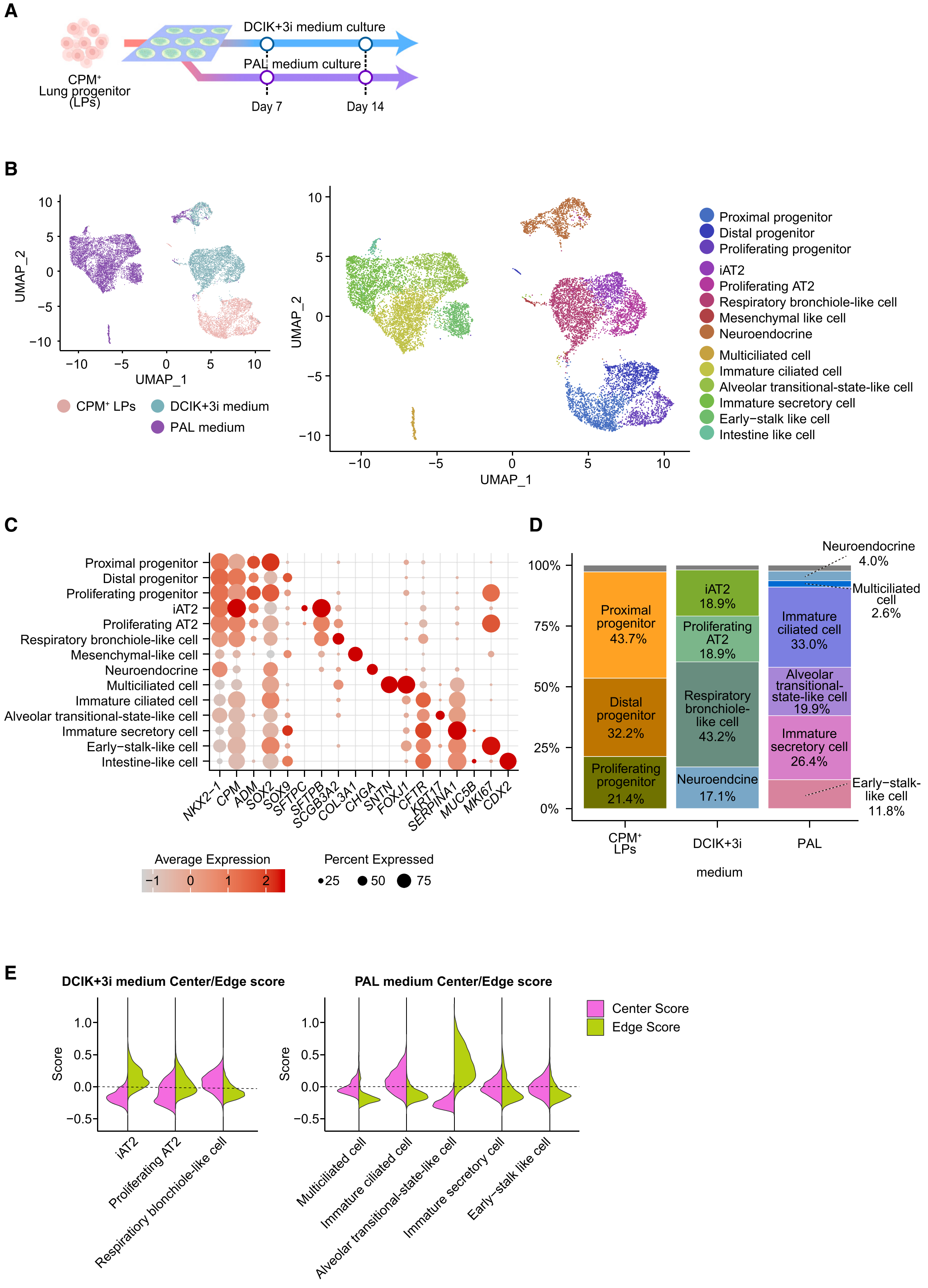

(legend on next page)

culture to snRNA-seq transcriptomes data of primary pulmonary epithelial cells at the fetal, juvenile, and adult stages (Wang et al., 2020) using uniform manifold approximation and projection (UMAP) (Figure S5). Our findings showed that iAT2 cells aligned more closely with fetal or juvenile AT2 cells (Figures S5A and S5B), but not adult cells (Figure S5C), indicating substantial resemblance to early-stage AT2 cells. Despite the limitations posed by integrating the different analytical methods, our findings showed that iAT2 cells aligned more closely with fetal or juvenile AT2 cells (Figures S5A and S5B), not adults (Figure S5C), indicating substantial resemblance to early-stage AT2 cells. In contrast, multiciliated cells overlapped with those from all stages, suggesting a high similarity to adult ones.

On day 7 of the DCIK+3i culture, 57.0% of the cells were iAT2 or proliferating AT2, and 31.7% were *SFTPB*$^{+}$ *SCGB3A2*$^{+}$ cells, mainly AT2-like and respiratory bronchiolar cells (Figure S4E). By day 14, the proportion of iAT2 and proliferating AT2 decreased to 16.0%, whereas *SFTPC* was upregulated, indicating a more refined AT2 cell population (Figure S4F). On day 7 in the PAL culture, 49.7% of the population comprised multiciliated or immature ciliated cells, whereas the rest were more immature airway epithelial cells. By day 14, the proportion of ciliated cell clusters decreased to 13.5%, whereas that of immature secretory cells increased to 58.9% (Figure S4E).

Micro-patterned culture is suitable for modeling the SARS-CoV-2 infection model

We conducted infection experiments with five SARS-CoV-2 variants (MOI 0.1) in micro-patterned cultures: B.1.1.214, B.1.617.2 (delta), BA.1, BA.2, and BA.5. The alveolar epithelial cells were infected on day 6, once GFP^{+} cells appeared, and airway epithelial cells were infected on day 11, after multiciliated cell detection (Figure 5A). Viral amplification in alveolar and airway epithelial cells was assessed based on detectable viral genomes in the supernatant (Figures 5B and 5C). In alveolar epithelial cells, the B.1.1.214- and B.1.617.2-infected groups released many viruses continuously (maximum: 2.14×10^5 and 2.34×10^5 viral copy number/μL, respectively). In contrast, the Omicron variants (BA.1, BA.2, and BA.5) released fewer viruses (maximum: 3.74×10^3, 9.38×10^3, and 2.68×10^4 viral copy number/μL, respectively). In airway epithelial cells, B.1.617.2 continuously released the most virus (maximum: 9.85×10^5 viral copy number/μL); however, that released by B.1.1.214 and BA.5 was similar 3 days postinfection (dpi) (9.74×10^5 and 8.80×10^5 viral copy number/μL, respectively). Meanwhile, viral genome levels decreased at 4 dpi (0.437×10^3 and 2.64×10^3 viral copy number/μL, respectively). In addition, viral release into the supernatant from BA.1- and BA.2-infected airway cells was negligible (maximum: 321 and 219 viral copy number/μL, respectively) (Figure 5C).

Next, we collected intracellular RNA from alveolar and airway epithelial cells at 4 dpi and assessed viral gene expression via qRT-PCR (Figure 5D). Alveolar cells infected with B.1.1.214 and B.1.617.2 showed increased *SARS-CoV-2 N*, whereas in airway cells, only B.1.617.2 infected cells led to a persistent increase, indicating high tropism of this variant for both cells. *SARS-CoV-2 N* levels in Omicron variant-infected alveolar epithelial cells were lower, consistent with the virus release results. Meanwhile, BA.1, which rarely infects alveolar epithelial cells, showed *SARS-CoV-2 N* levels comparable to those of B.1.617.2 in airway epithelial cells. The discrepancy between the low virus release and high *SARS-CoV-2 N* levels in BA.1-infected airway cells may indicate differences in the extracellular virus release mechanisms of BA.1. *ACE2*—gene encoding a SARS-CoV-2 receptor—was downregulated in alveolar and airway epithelial cells, mirroring *SARS-CoV-2 N* levels. The levels of interferon response-related genes (*IFNA1*, *IFNB1*, and *MX1*) were elevated in cells infected by the B.1.617.2 and BA.2 variants. These results, not closely linked to

Figure 4. Cell-type scRNA-seq analysis of lung epithelial cells in the micro-patterned culture

(A) Schematic diagram of the samples used for analysis. *SFTPC*^{GFP} knockin reporter iPSC-derived CPM^{+} LPCs were seeded onto micro-patterned culture plates and induced to differentiate into alveolar or airway epithelial cells. The LPCs and differentiated cells at each time point were dissociated for scRNA-seq.

(B) UMAP projections of the consolidated data from all of the samples. Left: color-coded by culture conditions; right: color-coded by cell-type annotations after clustering.

(C) Dot plot showing gene expressions in different cell types. The x axis lists selected genes, and the y axis categorizes cell types. Dot size indicates the percentage of cells expressing each gene, and color shade represents the magnitude of the average expression level of each gene.

(D) Stacked bar chart showing the distribution of each cell type for LPCs and cultured samples. The first bar represents the LPCs labeled as CPM, and the subsequent bars represent samples cultured in DCiK+3i and PAL media, respectively. The color-coded segments and corresponding labels indicate the percentage of each cell type in the total population within each condition.

(E) Violin plots comparing gene expression scores in the cell populations cultured in DCIK+3i medium (left) and PAL medium (right). Each plot represents the distribution of Center (purple) and Edge (green) scores for specific cell types, indicating the variability within the colony regions.

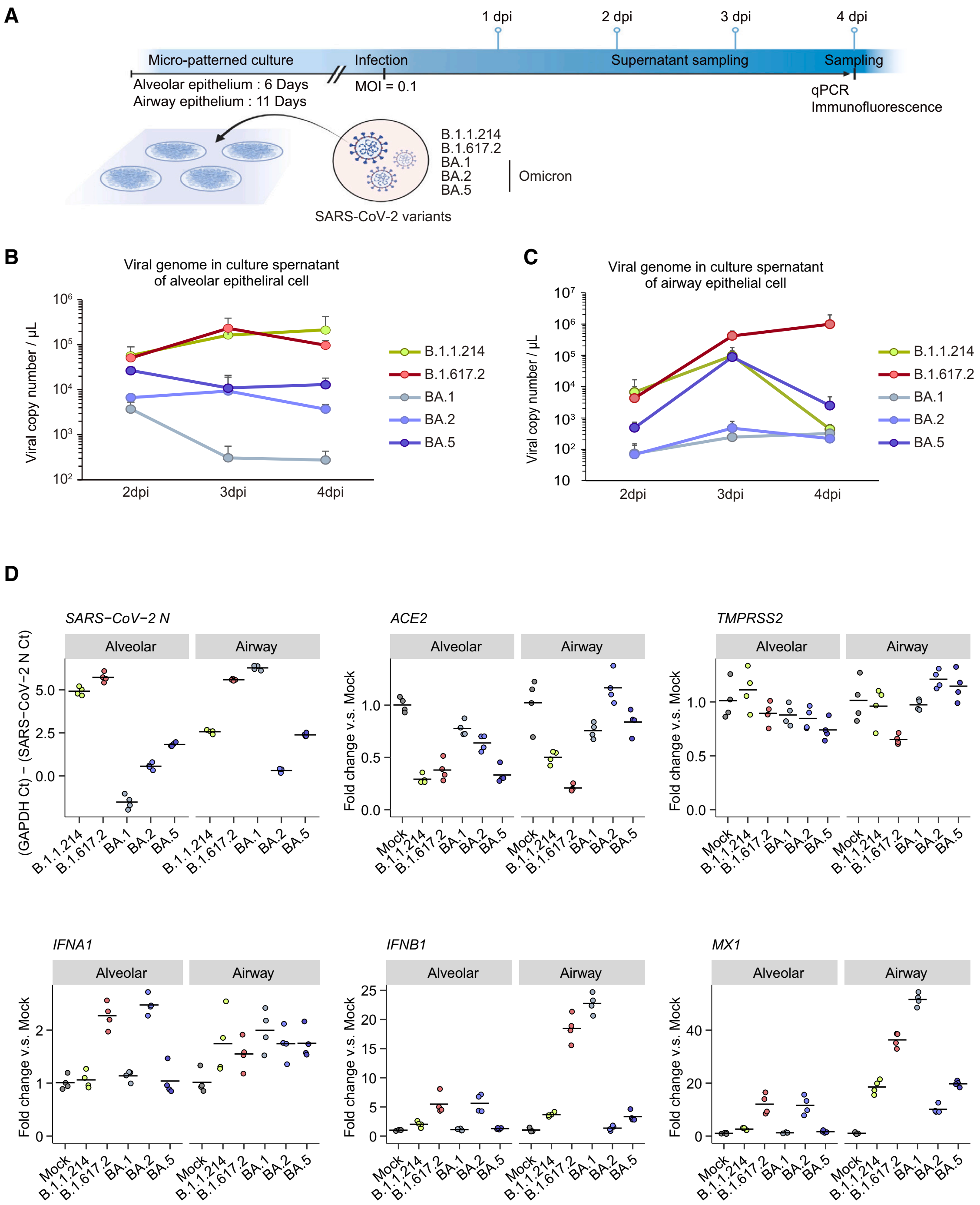

Figure 5. Tropism of SARS-CoV-2 variants in iPSC-derived alveolar and airway epithelial cells in the micro-patterned culture

(A) Schematic diagram of SARS-CoV-2 infection experiments. The iPSC-derived alveolar and airway epithelial cells in the micro-patterned culture plate were infected with each SARS-CoV-2 variant.

(B and C) The viral RNA copy number in the cell culture supernatant of iPSC-derived alveolar (B) and airway (C) epithelial cells at 2, 3, and 4 dpi were measured using qRT-PCR.

(D)Representative gene expression levels of iPSC-derived alveolar and airway cells at 4 dpi measured using qRT-PCR.

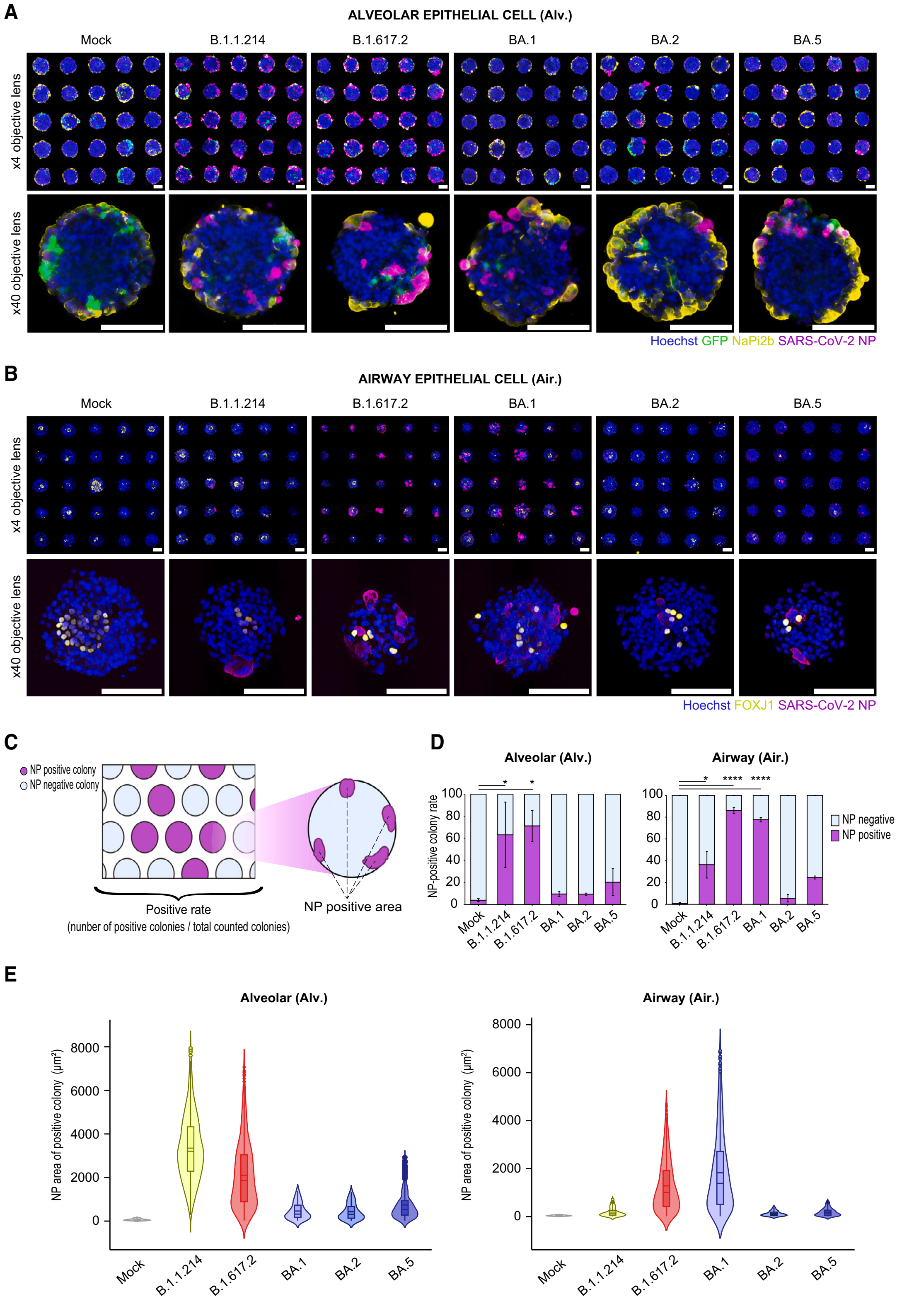

(legend on next page)

SARS-CoV-2 N levels, may reflect differences in the immune response intensity of each variant. Therefore, our model could distinguish between SARS-CoV-2 variants based on unique infection efficiency and the initiation of innate immune responses in alveolar and airway systems.

Quantification of SARS-CoV-2 variant tropism using micro-patterned alveolar and airway cells

SARS-CoV-2 infection was quantified at the protein level using immunofluorescence analysis. Lineage markers and SARS-CoV-2 nucleocapsid protein (NP) were analyzed in micro-patterned alveolar and airway epithelial cell cultures. SARS-CoV-2 NP was detected in the alveolar and airway epithelial cells of the SARS-CoV-2-infected group (Figures 6A and 6B). Subsequently, we segmented alveolar epithelial cell colonies into center and periphery and calculated NP^+ area percentages (Figure S6A). NP^+ cells were preferentially localized at the periphery of the colonies, where AT2s were detected (Figure S6B). Furthermore, we developed an infection-efficiency index based on quantitative image analysis. We considered the viral infection of numerous colonies stochastic and calculated the percentage of NP^+ colonies as an indicator of the tropism of each variant (Figure 6C). Approximately 1,000 colonies were detected in each well of the 24-well micro-patterned plate. A high percentage of SARS-CoV-2 NP^+ colonies was calculated in the B.1.1.214- and B.1.617.2-infected groups (63.0% ± 29.7% and 71.2% ± 14.1%, respectively). In contrast, the Omicron variants showed lower percentages: 9.4% ± 2.4% (BA.1), 9.3% ± 1.0% (BA.2), and 20.1% ± 12.2% (BA.5) (Figure 6D). These results correlated with the amount of virus released into the culture supernatant (Figure 5B) and intracellular *SARS-CoV-2 N* levels (Figure 5C). Hence, the percentage of SARS-CoV-2 NP^+ colonies effectively reflected the stochastic SARS-CoV-2 infection rate in target cells. Next, we quantified their NP^+ areas of NP^+ colonies (Figure 6C) as an index of intracellular viral proliferation and local propagation. The mean NP^+ areas were of the 3,350 μm^2 (B.1.1.214) and 2,102 μm^2 (B.1.617.2), larger than the Omicron variants 461.5 μm^2 (BA.1), 429.7 μm^2 (BA.2), and 739.4 μm^2 (BA.5) (Figure 6E). These results, correlating with intracellular *SARS-CoV-2 N* levels, indicate that both the percentage of NP^+ colonies and NP^+ areas are reliable indicators of variant tropism. The percentage of SARS-CoV-2 NP^+ colonies in micro-patterned airway epithelial cells was high following B.1.617.2 and BA.1 infection (86.3% ± 2.8% and 77.8% ± 2.0%, respectively) and low after B.1.1.214 (36.4% ± 12.3%), BA.2 (5.6% ± 3.5%) and BA.5 (24.6% ± 1.4%) infection (Figure 6D). Mean NP^+ areas for the B.1.617.2- and BA.1-infected groups were 1,341 and 1,558 μm^2, respectively, whereas those for B.1.1.214-, BA.2-, and BA.5-infected groups were 399.7, 106.8, and 226.2 μm^2, respectively (Figure 6E). These results correlated with intracellular *SARS-CoV-2 N* levels (Figure 6D). Conclusively, the high-tropism variants differed between alveolar and airway epithelial cells, indicating that SARS-CoV-2 tropism depends on the target cell lineages and variants.

Micro-patterned culture system distinguishes the pathogenesis of each SARS-CoV-2 variant

We next compared the transcriptomes of B.1.617.2- and BA.1-infected cells using principal-component analysis (PCA), revealing that SARS-CoV-2 infection shifted the plots parallel to the PC2 axis (Figure 7A). The BA.1-exposed micro-patterned alveolar cell transcriptomes overlapped with the mock control, corresponding to the low tropism of BA.1 for alveolar epithelial cells. These results suggest that the transcriptomic changes after SARS-CoV-2 infection were similar in micro-patterned alveolar and airway epithelial cells. We performed pathway enrichment analysis on the 26 DEGs common to the three infection groups: B.1.617.2 in micro-patterned alveolar and airway epithelial cells and BA.1 in micro-patterned airway epithelial cells (Figure 7B). In the common DEGs, the gene set related to IFN reactions was enriched (Figures 7C and 7D). These results suggested that IFN responses were induced in the alveolar and airway epithelial cells after SARS-CoV-2 infection. Subsequently, we compared the transcriptomes of the airway epithelial cells infected with B.1.617.2 or BA.1, which exhibited comparable tropism, although virus release was relatively limited in the BA.1 infection culture (Figure 7E). We investigated 502 DEGs specific to the B.1.617.2-infected airway epithelial cells. The regulation of apoptotic signaling pathway and the positive regulation of apoptotic process were enriched in the 306 upregulation genes (Figure 7F). We then performed TUNEL staining for the cells at 4 dpi in the SARS-CoV-2 mutant infection

Figure 6. Image analyses of iPSC-derived alveolar and airway epithelial cells to characterize SARS-CoV-2 variants

(A and B) Immunofluorescent images of SARS-CoV-2-infected alveolar (A) and airway (B) epithelial cells in a micro-patterned culture plate at 4 dpi. Scale bar: 100 μm. Top: low-magnification images; bottom: high-magnification images.

(C) Schematic of a SARS-CoV-2 NP^+ colony. The number of SARS-CoV-2 NP^+ colonies was defined as the percentage of colonies positive for SARS-CoV-2 NP. The SARS-CoV-2 NP^+ area was defined as the area of SARS-CoV-2 NP signals in the N $signal^+$ colonies.

(D) Rate of SARS-CoV-2 NP^+ colonies in alveolar and airway epithelial cells infected with each SARS-CoV-2 variant. Data are mean ± SEM (n = 3 independent experiments). Two-way ANOVA with Dunnett's multiple comparison tests; *p < 0.05, ****p < 0.0001.

(E) Violin plots of SARS-CoV-2 NP^+ areas in alveolar and airway epithelial cells infected with each SARS-CoV-2 variant.

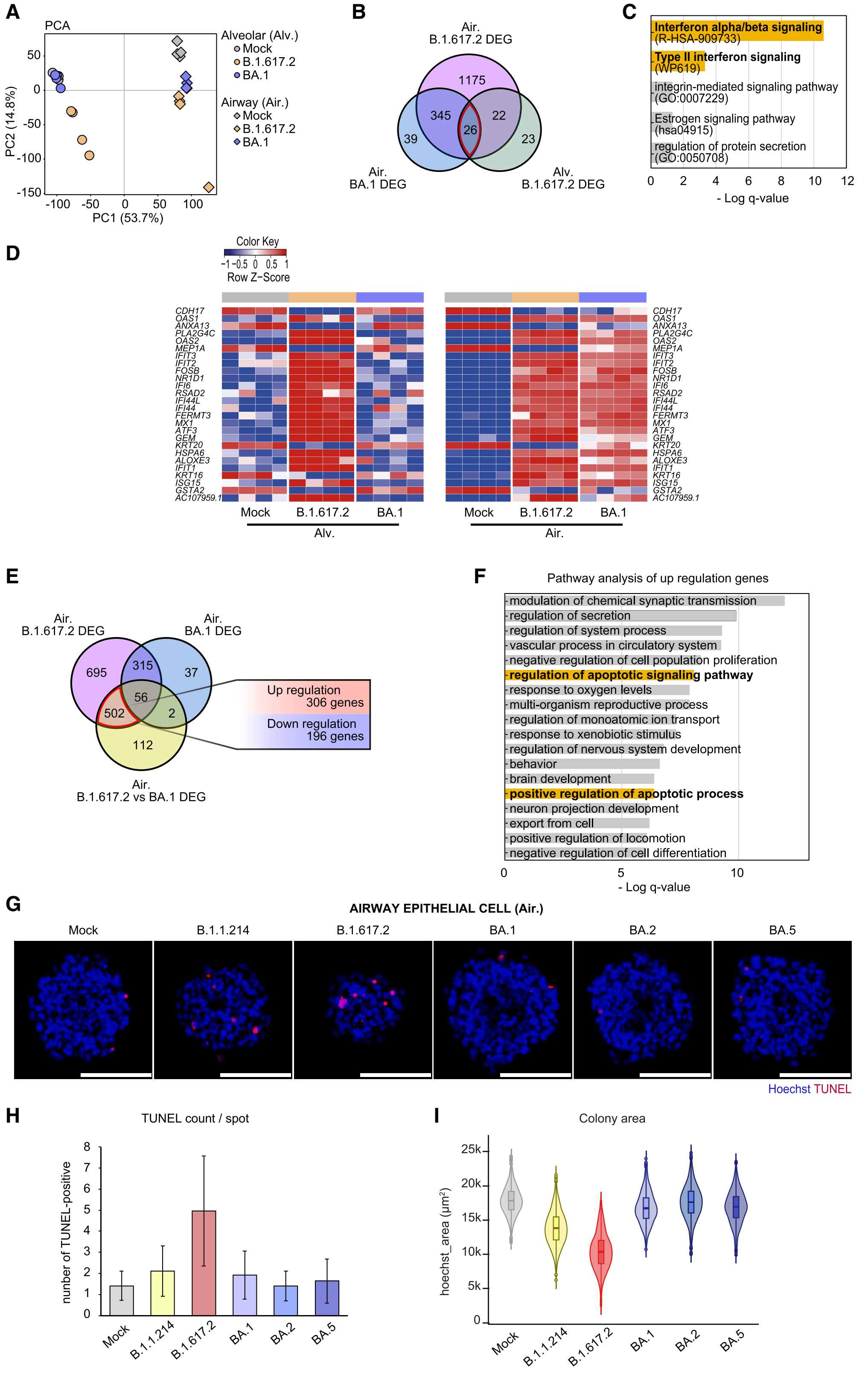

(legend on next page)

experiment (Figures 7G and S7A). More TUNEL$^+$ cells were observed in the B.1.617.2-infected group (4.96 ± 2.61 count/colony) than in the other variant-infected groups (Figures 7H and S7B). In addition, the colony size of the B.1.617.2-infected group (10.4 ± 2.64 × 10^3 μm^2) was smaller than that of other variant-infected groups and served as a surrogate indicator of apoptosis (Figure 7I). These results suggest that the B.1.617.2 variant induces apoptosis in micro-patterned airway epithelial cells and that the micro-patterned culture system can be adopted to detect unique variant phenotypes.

DISCUSSION

AT2s are notorious for their rapid change when cultured on plastic surfaces (Borok et al., 1998; Foster et al., 2007). Although successfully grown in 3D cultures embedded in ECM, such as Matrigel, their methods and image analyses are still complicated. In addition, AT2s in spheroids within Matrigel orient their apical surfaces toward the lumen, representing a considerable obstacle to modeling SARS-CoV-2 infection (Mulay et al., 2021; Salahudeen et al., 2020; Tamai et al., 2022). Micro-patterned culture is expected to address these issues. In the present study, we demonstrated that the separately cultured iPSC-derived alveolar and airway epithelial cells in micro-patterned plates could distinguish SARS-CoV-2 variants. The cells within the heterogeneous cell colonies on micro-patterned plates seem to be from two to four overlaid layers (Video S2) with high cell density. Importantly, iAT2s in micro-patterned cultures were apical-out, exposing their apical surface to the medium without the additional steps of enzymatic digestion of the Matrigel or culture in suspension. In contrast, highly confluent cells at the center of the colonies were suitable for inducing multiciliated airway cells.

Distinct staining properties of various dyes, including Hoechst, within the center and periphery of micro-patterned iPSC colonies have been reported (Kim et al., 2022). This finding was replicated in our micro-patterned cultures, aiding in separating central and peripheral colony regions for transcriptome analysis and elucidating that the position-specific signals differed between the center and periphery of the colonies. Specifically, the WNT signal was suppressed at the periphery of the colonies, consistent with the previous finding that appropriate WNT inhibition promoted AT2 differentiation (Jacob et al., 2017). Meanwhile, RNA-seq analysis revealed the upregulation of Notch signaling in the central region, indicating its contribution to the differentiation of airway lineages. The enrichments of NOTCH signaling- and airway progenitor cell-related genes in the center of the colony are consistent with the early stages of airway development in mice (Guha et al., 2012; Stupnikov et al., 2019). In addition, the suppression of NOTCH signaling by DAPT (a γ-secretase inhibitor) in the airway epithelial induction medium promoted multiciliated cell differentiation, consistent with a previous report (Rock et al., 2011). These results support the notion that position-specific signals within the colonies of the micro-patterned culture plates can facilitate alveolar and airway cell induction.

The different infection profiles of SARS-CoV-2 variants were reflected in various parameters. For example, quantifying virus release into the culture supernatant recapitulated viral spread in the lung epithelium. Moreover, analysis of intracellular gene expression levels, quantification of the number and size of infected colonies, and assessment of the abundance of viral antigen and other essential markers were performed using the two major lung lineages in a micro-patterned culture. This study proposes an unprecedented parameter, the percentage of SARS-CoV-2 N$^+$ colonies, as an index of infection efficiency. This hypothesis is based on the idea that colony viral infection occurs

Figure 7. Comparing SARS-CoV-2 variants elucidates viral pathogenicity

(A) PCA of the transcriptomes of the alveolar and airway epithelial cells infected with or without SARS-CoV-2: B.1.617.2 or BA.1. log (TPM value) was used for the PCA.

(B) A total of 26 genes were extracted as commonly altered in DEGs between mock- and B.1.617.2-infected alveolar epithelial cells and those among mock-, B.1.617.2-, and BA.1-infected airway epithelial cells.

(C) GO analysis of the 26 extracted genes.

(D) Heatmaps presented with *Z* scores of the 26 extracted genes from the alveolar (left) and airway (right) epithelial cells infected with or without SARS-CoV-2: B.1.617.2 or BA.1. *Z* scores were calculated from log (TPM value) (n = 4 biological replicates).

(E) The 502 genes were extracted from DEGs between mock vs. B.1.617.2 infection in the airway epithelial cells, excluding DEGs between mock vs. BA.1. The 509 genes comprised 306 upregulated and 196 downregulated genes.

(F) GO analysis of the 306 upregulated genes.

(G) Representative TUNEL staining images of the SARS-CoV-2-infected airway epithelial cells in the micro-patterned culture plate at 4 dpi. Scale bar: 100 μm.

(H) The number of TUNEL$^+$ cells within the SARS-CoV-2-infected airway epithelial cell colonies in the micro-patterned culture plate at 4 dpi. Data are mean ± SD. The number of colonies ranged from 498 to 1,110 from 1 representative experiment.

(I) Violin plot of the Hoechst area of airway epithelial cells infected with each variant. All of the colonies were quantified. The number of colonies ranged from 1,133 to 1,169 from 1 representative experiment.

stochastically and that the ratio of infected colonies depends on the viral tropism for cells.

Our results indicated that each variant had different infection efficiencies in the two major lung regions, alveolar and airway lineages, and that the efficient infection of B.1.1.214 and B.1.617.2 in iPSC-derived alveolar epithelial cells was consistent with the clinical phenotypes of the early SARS-CoV-2 variants, which frequently caused virus-induced pneumonia (van Doremalen et al., 2022). In addition, the Delta variant (B.1.617.2) released the largest number of viral genomes and proliferated the most actively among the SARS-CoV-2 variants (B.1.1.214, B.1.617.2, BA.1, BA.2, and BA.5) in the airway and alveolar lineages, and induced apoptosis in airway epithelial cells. These features of B.1.617.2 may be associated with its invasiveness, which caused severe pneumonia at a higher frequency than the other variants (Li et al., 2020). Furthermore, our results show that the Omicron variant (BA.1, BA.2, and BA.5) has distinct features. BA.2 shows higher viral release than BA.1 in alveolar epithelial cells, whereas BA.5 exhibits the strongest viral release among the three variants and relatively high intracellular proliferation capacity in the airway and alveolar lineages. These differences without the immune system may suggest that evading immunity was not the sole reason for the main pandemic variant being replaced by BA.1 to BA.2 and BA.5 (Kimura et al., 2022). Our study differs from a previous report on patterning lung bud cultures derived from human ESCs (Rosado-Olivieri et al., 2023) because our system enables detailed analysis of SARS-CoV-2 variants by separately inducing alveolar and airway epithelial cells with each lineage benchmarked. Hence, our system has the advantage of supporting the evaluation of differences among variants, particularly in terms of their tropism and phenotypic characteristics.

In conclusion, our culture system enables the robust induction of iPSC-derived alveolar and airway cells. The position-specific appearance of AT2s and multiciliated airway cells allows for the characterization of crucial signals required for their induction. Our results indicate that this strategy can be effectively adopted to analyze the pathogenesis of known SARS-CoV-2 variants, but also for characterizing the emerging SARS-CoV-2 variants. In this way, our system can be applied to predict the severity of new variants before they become prevalent.

EXPERIMENTAL PROCEDURES

Resource availability

Lead contact

Further information and requests for resources and reagents should be directed to and will be fulfilled by the corresponding authors. The lead contact is Shimpei Gotoh (gotoh.shimpei.5m@kyoto-u.ac.jp).

Materials availability

The materials included in this study are available from the corresponding authors upon reasonable request.

Data and code availability

The accession numbers for the RNA-seq and scRNA-seq raw data reported in the present study are GEO: GSE236842 and GSE249762.

Micro-patterned plate culture

The magnetic-activated cell sorting (MACS) -isolated LPCs were seeded in 24-well micro-patterned culture plates (2.5D culture plates) (Tosoh) with multiple round cell-attachment areas in a 100- or 200-μm diameter precoated with 0.5 μg/cm^2 of iMatrix-511 silk (TaKaRa Bio, 892021) at cell densities ranging from 0.1 to 3 × 10^5 cells/well. The LPCs were differentiated into alveolar epithelial cells in DCIK+3i medium: Ham's F12 (Fujifilm Wako, 087-08335) containing 50 nM dexamethasone (Sigma-Aldrich, D4902), 100 μM 8-Br-cAMP (Biolog Life Science Institute, B007), 100 μM 3-isobutyl-1-methylxanthine (Fujifilm Wako, 099–03411), 10 ng/mL keratinocyte growth factor (KGF), 1% B-27 supplement, 0.25% bovine albumin fraction V (Thermo Fisher Scientific, 15260-037), 15 mM HEPES (Thermo Fisher Scientific, 17557-94), 0.8 mM $CaCl_2$ (Fujifilm Wako, 036-19731), 0.1% ITS premix (Corning, 354352), 50 U/mL penicillin/streptomycin, 3 μM CHIR-99021, 10 μM SB431542 (Fujifilm Wako, 198-16543), and 10 μM Y27632 (LC Laboratories, Y-5301). The LPCs were differentiated into airway epithelial cells in the medium as follows: PneumaCult-ALI medium (Veritas, ST-05001) containing 500 nM hydrocortisone (Sigma-Aldrich, H4001), 4 μM heparin (Nacalai Tesque, 17513-54), and 10 μM Y27632 for the initial 2 days and the same medium but supplemented with 10 μM DAPT ("PAL medium") for the remaining period. The medium was changed every 2 days.

SARS-CoV-2 variant infection postmicro-patterning culture

Alveolar or airway epithelial cells differentiated in 24-well micro-patterned culture plates were infected with 0.1 MOI SARS-CoV-2 for 2 h. Infected alveolar or airway epithelial cells were cultured in DCIK+3i medium or PneumaCult-ALI medium for 4 days. The culture medium was changed daily after infection.

Statistical analysis

Data are presented as mean ± SEM. The number of biological replicates and statistical tests is described in each figure legend. All of the statistical tests were performed using Prism7 software (GraphPad). Statistical significance was set at $p < 0.05$.

SUPPLEMENTAL INFORMATION

Supplemental information can be found online at https://doi.org/10.1016/j.stemcr.2024.02.011.

ACKNOWLEDGMENTS

We thank Tosoh Ltd. for providing the 2.5D culture plates; Ms. Naoko Yasuhara, Ms. Ayaka Sakamoto, Ms. Natsumi Mimura, and Mr. Hiroki Futatsusako at CiRA, Kyoto University, for performing

the experiments and analyses of SARS-CoV-2 infection; Ms. Kazusa Okita, Ms. Satoko Sakurai, and Ms. Kazumi Deguchi at the genome analysis group in the CiRA common equipment management office for RNA-seq library preparation and sequencing with analysis; Dr. Yoshio Koyanagi, Dr. Kazuya Shimura, and Dr. Yukiko Muramoto at Kyoto University for the setup and operation of the BSL-3 laboratory at Kyoto University; Dr. Shinya Yamanaka, Dr. Knut Woltjen, Dr. Yoshinori Yoshida, Dr. Naoyuki Sone, Dr. Ryuta Mikawa, Dr. Yuko Ohnishi, and Dr. Yusuke Tsutsui at CiRA, Kyoto University, and Dr. Toyohiro Hirai and Dr. Masatoshi Hagiwara at Graduate School of Medicine, Kyoto University, for helpful discussions and comments; all of the members of the Center for Anatomical, Pathological, and Forensic Medical Research, Kyoto University, for electron microscopy; and all of the members of the Medical Research Support Center, Kyoto University, for adjustment of laboratory equipment. This study was funded by The COVID-19 Private Fund (to Dr. Shinya Yamanaka's laboratory, CiRA), AMED-CREST (JP21gm1610005), the JST Core Research for Evolutional Science and Technology (JPMJCR20HA), the Joint Usage/Research Center Program of Institute for Life and Medical Sciences at Kyoto University, the iPS Cell Research Fund for CiRA at Kyoto University, AMED (JP17bm0804007, JP22bm1123013, and JP23bm1323001), JSPS KAKENHI (JP22K19525 and JP22H03077) and Research Fund to Department of Drug Discovery for Lung Diseases at Kyoto University from Kyorin Pharmaceutical Co. Ltd.

AUTHOR CONTRIBUTIONS

Conceptualization: A.M., K.T., and S.G.; methodology: A.M., K.T., and S.G.; validation: R.H.; formal analysis: A.M., R.H., and T.Y.; investigation: A.M. and R.H.; resources: Y.M., M.N., and T.N.; data curation: A.M., R.H., and T.Y.; writing – original draft: A.M., and S.G.; writing – review & editing: A.M., K.T., and S.G.; visualization: A.M.; supervision: M.N., T.N., K.T., and S.G.; project administration: K.T. and S.G.; and funding acquisition: T.N., K.T., and S.G.

DECLARATION OF INTERESTS

A.M. is an employee and shareholder of Kyorin Pharmaceutical Co. Ltd. S.G. is a founder, shareholder, and an external board member of HiLung. S.G. filed the patents related to the methods of generating iPSC-derived lung cells in this study: WO2014168264A1, WO2016143803A1, and WO2016148307A1.

Received: August 6, 2023
Revised: February 27, 2024
Accepted: February 28, 2024
Published: March 28, 2024

REFERENCES

Basil, M.C., Cardenas-Diaz, F.L., Kathiriya, J.J., Morley, M.P., Carl, J., Brumwell, A.N., Katzen, J., Slovik, K.J., Babu, A., Zhou, S., et al. (2022). Human distal airways contain a multipotent secretory cell that can regenerate alveoli. Nature *604*, 120–126. https://doi.org/10.1038/s41586-022-04552-0.

Borok, Z., Danto, S.I., Lubman, R.L., Cao, Y., Williams, M.C., and Crandall, E.D. (1998). Modulation of T1α expression with alveolar epithelial cell phenotype in vitro. Am. J. Physiol. *275*, 155–164. https://doi.org/10.1152/ajplung.1998.275.1.l155.

van Doremalen, N., Singh, M., Saturday, T.A., Yinda, C.K., Perez-Perez, L., Bohler, W.F., Weishampel, Z.A., Lewis, M., Schulz, J.E., Williamson, B.N., et al. (2022). SARS-CoV-2 Omicron BA.1 and BA.2 are attenuated in rhesus macaques as compared to Delta. Sci. Adv. *8*, eade1860. https://doi.org/10.1126/sciadv.ade1860.

Foster, C.D., Varghese, L.S., Skalina, R.B., Gonzales, L.W., and Guttentag, S.H. (2007). In Vitro Transdifferentiation of Human Fetal Type II Cells Toward a Type I–like Cell. Pediatr. Res. *61*, 404–409. https://doi.org/10.1203/pdr.0b013e3180332c6d.

Gotoh, S., Ito, I., Nagasaki, T., Yamamoto, Y., Konishi, S., Korogi, Y., Matsumoto, H., Muro, S., Hirai, T., Funato, M., et al. (2014). Generation of Alveolar Epithelial Spheroids via Isolated Progenitor Cells from Human Pluripotent Stem Cells. Stem Cell Rep. *3*, 394–403. https://doi.org/10.1016/j.stemcr.2014.07.005.

Guha, A., Vasconcelos, M., Cai, Y., Yoneda, M., Hinds, A., Qian, J., Li, G., Dickel, L., Johnson, J.E., Kimura, S., et al. (2012). Neuroepithelial body microenvironment is a niche for a distinct subset of Clara-like precursors in the developing airways. Proc. Natl. Acad. Sci. USA *109*, 12592–12597. https://doi.org/10.1073/pnas.1204710109.

Hawkins, F., Kramer, P., Jacob, A., Driver, I., Thomas, D.C., McCauley, K.B., Skvir, N., Crane, A.M., Kurmann, A.A., Hollenberg, A.N., et al. (2017). Prospective isolation of NKX2-1-expressing human lung progenitors derived from pluripotent stem cells. J. Clin. Invest. *127*, 2277–2294. https://doi.org/10.1172/JCI89950.

He, P., Lim, K., Sun, D., Pett, J.P., Jeng, Q., Polanski, K., Dong, Z., Bolt, L., Richardson, L., Mamanova, L., et al. (2022). A human fetal lung cell atlas uncovers proximal-distal gradients of differentiation and key regulators of epithelial fates. Cell *185*, 4841–4860.e25. https://doi.org/10.1016/j.cell.2022.11.005.

Huang, J., Hume, A.J., Abo, K.M., Werder, R.B., Villacorta-Martin, C., Alysandratos, K.D., Beermann, M.L., Simone-Roach, C., Lindstrom-Vautrin, J., Olejnik, J., et al. (2020). SARS-CoV-2 Infection of Pluripotent Stem Cell-Derived Human Lung Alveolar Type 2 Cells Elicits a Rapid Epithelial-Intrinsic Inflammatory Response. Cell Stem Cell *27*, 962–973.e7. https://doi.org/10.1016/j.stem.2020.09.013.

Jacob, A., Morley, M., Hawkins, F., McCauley, K.B., Jean, J.C., Heins, H., Na, C.L., Weaver, T.E., Vedaie, M., Hurley, K., et al. (2017). Differentiation of Human Pluripotent Stem Cells into Functional Lung Alveolar Epithelial Cells. Cell Stem Cell *21*, 472–488.e10. https://doi.org/10.1016/j.stem.2017.08.014.

Kadur Lakshminarasimha Murthy, P., Sontake, V., Tata, A., Kobayashi, Y., Macadlo, L., Okuda, K., Conchola, A.S., Nakano, S., Gregory, S., Miller, L.A., et al. (2022). Human distal lung maps and lineage hierarchies reveal a bipotent progenitor. Nature *604*, 111–119. https://doi.org/10.1038/s41586-022-04541-3.

Kim, Y., Jang, H., Seo, K., Kim, J.H., Lee, B., Cho, H.M., Kim, H.J., Yang, E., Kim, H., Gim, J.-A., et al. (2022). Cell position within human pluripotent stem cell colonies determines apical specialization via an actin cytoskeleton-based mechanism. Stem Cell Rep. *17*, 68–81. https://doi.org/10.1016/j.stemcr.2021.11.005.

Kimura, I., Yamasoba, D., Tamura, T., Nao, N., Suzuki, T., Oda, Y., Mitoma, S., Ito, J., Nasser, H., Zahradnik, J., et al. (2022). Virological characteristics of the SARS-CoV-2 Omicron BA.2 subvariants, including BA.4 and BA.5. Cell *185*, 3992–4007.e16. https://doi.org/10.1016/j.cell.2022.09.018.

Kobayashi, Y., Tata, A., Konkimalla, A., Katsura, H., Lee, R.F., Ou, J., Banovich, N.E., Kropski, J.A., and Tata, P.R. (2020). Persistence of a regeneration-associated, transitional alveolar epithelial cell state in pulmonary fibrosis. Nat. Cell Biol. *22*, 934–946. https://doi.org/10.1038/s41556-020-0542-8.

Konishi, S., Gotoh, S., Tateishi, K., Yamamoto, Y., Korogi, Y., Nagasaki, T., Matsumoto, H., Muro, S., Hirai, T., Ito, I., et al. (2016). Directed Induction of Functional Multi-ciliated Cells in Proximal Airway Epithelial Spheroids from Human Pluripotent Stem Cells. Stem Cell Rep. *6*, 18–25. https://doi.org/10.1016/j.stemcr.2015.11.010.

Li, S., Zhang, Y., Guan, Z., Li, H., Ye, M., Chen, X., Shen, J., Zhou, Y., Shi, Z.-L., Zhou, P., and Peng, K. (2020). SARS-CoV-2 triggers inflammatory responses and cell death through caspase-8 activation. Signal Transduct. Targeted Ther. *5*, 235. https://doi.org/10.1038/s41392-020-00334-0.

Mulay, A., Konda, B., Garcia, G., Jr., Yao, C., Beil, S., Villalba, J.M., Koziol, C., Sen, C., Purkayastha, A., Kolls, J.K., et al. (2021). SARS-CoV-2 infection of primary human lung epithelium for COVID-19 modeling and drug discovery. Cell Rep. *35*, 109055. https://doi.org/10.1016/j.celrep.2021.109055.

Rock, J.R., Gao, X., Xue, Y., Randell, S.H., Kong, Y.-Y., and Hogan, B.L.M. (2011). Notch-dependent differentiation of adult airway basal stem cells. Cell Stem Cell *8*, 639–648. https://doi.org/10.1016/j.stem.2011.04.003.

Rosado-Olivieri, E.A., Razooky, B., Le Pen, J., De Santis, R., Barrows, D., Sabry, Z., Hoffmann, H.-H., Park, J., Carroll, T.S., Poirier, J.T., et al. (2023). Organotypic human lung bud microarrays identify BMP-dependent SARS-CoV-2 infection in lung cells. Stem Cell Rep. *18*, 1107–1122. https://doi.org/10.1016/j.stemcr.2023.03.015.

Salahudeen, A.A., Choi, S.S., Rustagi, A., Zhu, J., van Unen, V., de la O, S.M., Flynn, R.A., Margalef-Català, M., Santos, A.J.M., Ju, J., et al. (2020). Progenitor identification and SARS-CoV-2 infection in human distal lung organoids. Nature *588*, 670–675. https://doi.org/10.1038/S41586-020-3014-1.

Stupnikov, M.R., Yang, Y., Mori, M., Lu, J., and Cardoso, W.V. (2019). Jagged and Delta-like ligands control distinct events during airway progenitor cell differentiation. Elife *8*, e50487. https://doi.org/10.7554/eLife.50487.

Tamai, K., Sakai, K., Yamaki, H., Moriguchi, K., Igura, K., Maehana, S., Suezawa, T., Takehara, K., Hagiwara, M., Hirai, T., et al. (2022). iPS cell-derived mesenchymal cells that support alveolar organoid development. Cell Reports Methods *100314*. https://doi.org/10.1016/j.crmeth.2022.100314.

Wang, A., Chiou, J., Poirion, O.B., Buchanan, J., Valdez, M.J., Verheyden, J.M., Hou, X., Kudtarkar, P., Narendra, S., Newsome, J.M., et al. (2020). Single cell multiomic profiling of human lung reveals cell type-specific and age-dynamic control of SARS-CoV2 host genes. Elife *9*, 1–28. https://doi.org/10.7554/eLife.62522.

Yamamoto, Y., Gotoh, S., Korogi, Y., Seki, M., Konishi, S., Ikeo, S., Sone, N., Nagasaki, T., Matsumoto, H., Muro, S., et al. (2017). Long-term expansion of alveolar stem cells derived from human iPS cells in organoids. Nat. Methods *14*, 1097–1106. https://doi.org/10.1038/nmeth.4448.

Stem Cell Reports, Volume *19*

Supplemental Information

Micro-patterned culture of iPSC-derived alveolar and airway cells distinguishes SARS-CoV-2 variants

Atsushi Masui, Rina Hashimoto, Yasufumi Matsumura, Takuya Yamamoto, Miki Nagao, Takeshi Noda, Kazuo Takayama, and Shimpei Gotoh

SUPPLEMENTAL INFORMATION

Supplemental Figures

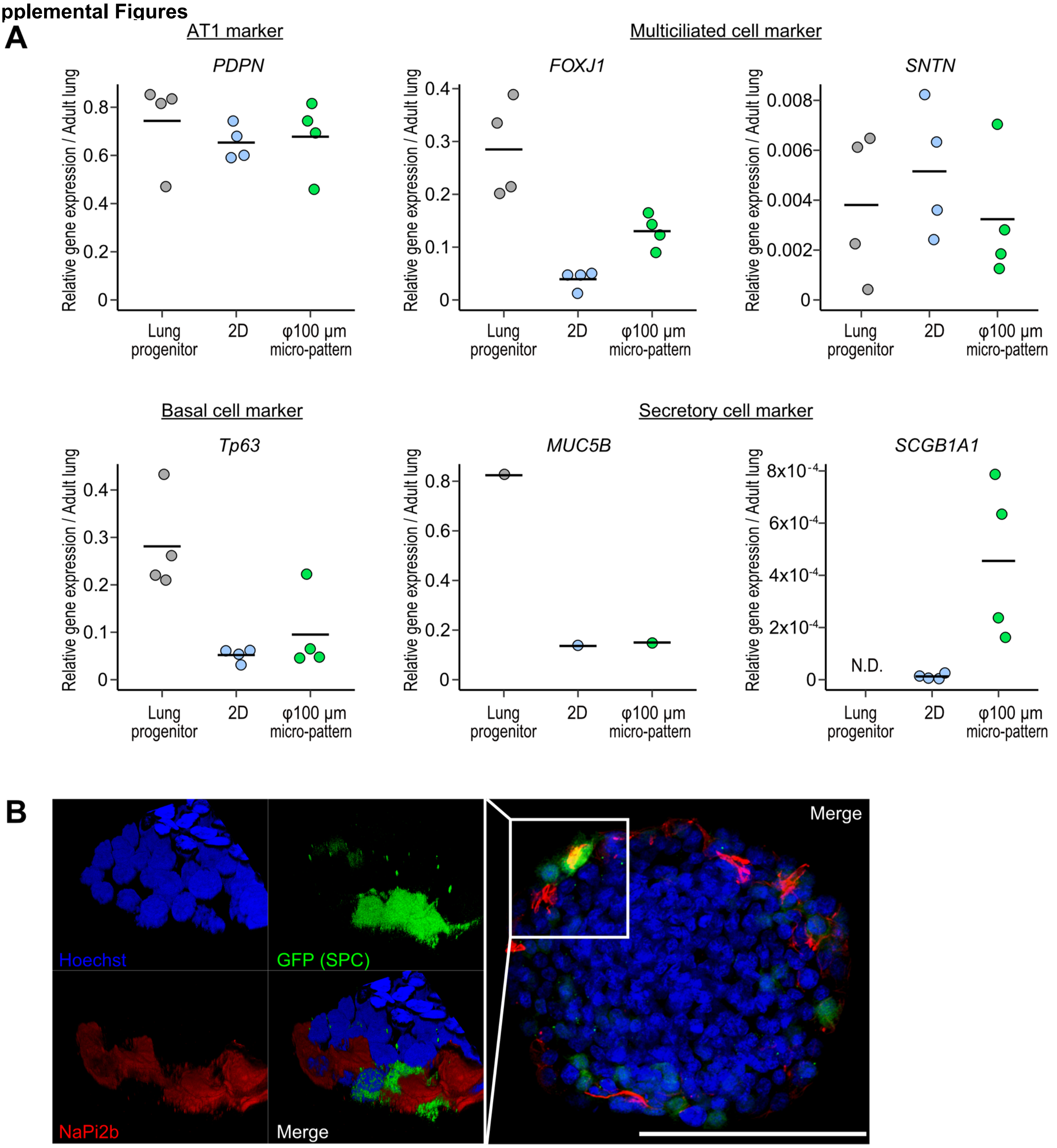

Figure S1. AT2s cultured in micro-patterned culture plates exhibit the apical side outward. Related to Figure 1.
A. Gene expressions of alveolar cells in micro-patterned or conventional 2D culture measured by qRT-PCR (n = 4 independent experiments).
B. 3D reconstructed imaging of alveolar epithelial cells cultured in micro-patterned plates. NaPi2B covers the surface of *SFTPC*^{GFP}-positive cells. Scale bar: 100 μm.

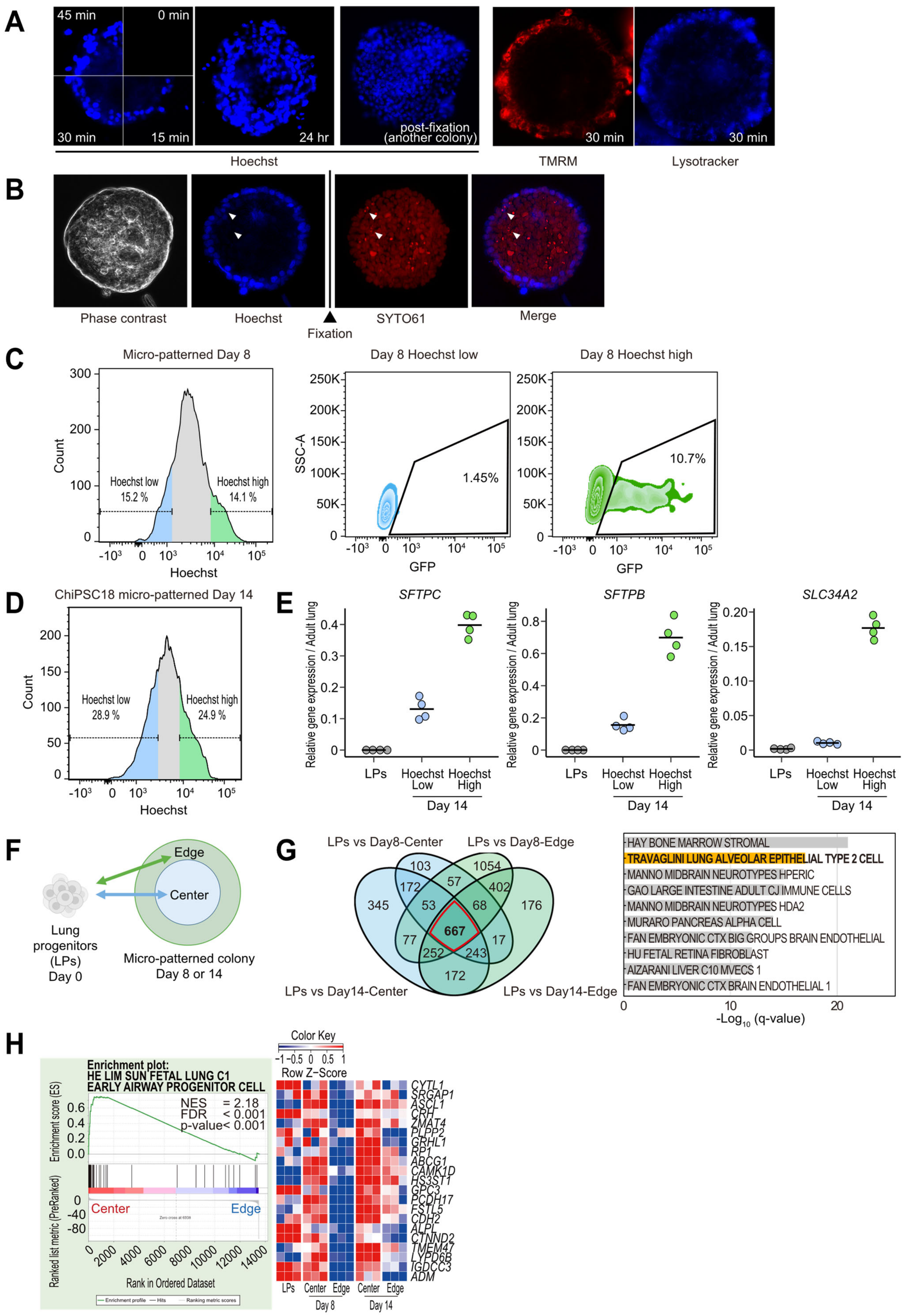
A
45 min
0 min
30 min
15 min
24 hr
post-fixation
(another colony)
30 min
30 min
Hoechst
TMRM
Lysotracker
B
Phase contrast
Hoechst
SYTO61
Merge
Fixation
C
Micro-patterned Day 8
Count
Hoechst low
15.2 %
Hoechst high
14.1 %
Hoechst
Day 8 Hoechst low
Day 8 Hoechst high
SSC-A
1.45%
10.7%
GFP
GFP
D
ChiPSC18 micro-patterned Day 14
Count
Hoechst low
28.9 %
Hoechst high
24.9 %
Hoechst
E
SFTPC
SFTPB
SLC34A2
Relative gene expression / Adult lung
LPs
Hoechst Low
Hoechst High
Day 14
F
Lung progenitors (LPs) Day 0
Edge
Center
Micro-patterned colony Day 8 or 14
G
LPs vs Day8-Center
LPs vs Day8-Edge
LPs vs Day14-Center
LPs vs Day14-Edge
103
1054
172
57
402
345
53
68
176
667
77
17
252
243
172
HAY BONE MARROW STROMAL
TRAVAGLINI LUNG ALVEOLAR EPITHELIAL TYPE 2 CELL
MANNO MIDBRAIN NEUROTYPES HPERIC
GAO LARGE INTESTINE ADULT CJ IMMUNE CELLS
MANNO MIDBRAIN NEUROTYPES HDA2
MURARO PANCREAS ALPHA CELL
FAN EMBRYONIC CTX BIG GROUPS BRAIN ENDOTHELIAL
HU FETAL RETINA FIBROBLAST
AIZARANI LIVER C10 MVECS 1
FAN EMBRYONIC CTX BRAIN ENDOTHELIAL 1
-Log10 (q-value)
H
Enrichment plot:
HE LIM SUN FETAL LUNG C1
EARLY AIRWAY PROGENITOR CELL
NES = 2.18
FDR < 0.001
p-value< 0.001
Enrichment score (ES)
Ranked list metric (PreRanked)
Center
Edge
Rank in Ordered Dataset
Color Key
Row Z-Score
CYTL1
SRGAP1
ASCL1
CRH
ZMAT4
PLPP2
GRHL1
RP1
ABCG1
CAMK1D
HS3ST1
GPC3
PCDH17
FSTL5
CDH2
ALPL
CTNND2
TMEM47
LYPD6B
IGDCC3
ADM
LPs
Center
Edge
Center
Edge
Day 8
Day 14

Figure S2. Living AT2 colonies show high dye staining at the periphery for short periods. Related to Figure 2.

A. Live cell imaging of the Hoechst-, TMRM-, or Lysotracker-stained micro-patterned alveolar cell colonies. Cells in the post-fixation cells images were fixed in 4% PFA/PBS for 15 min and Hoechst-stained.

B. Images of live and fixed lung epithelial progenitor cells pattern-cultured for 8 days in DCIK+3i medium. Live cells were stained with Hoechst for 30 min, and phase contrast and fluorescence images were captured. The fixed cells were stained with SYTO61 for nuclear staining. Arrowheads indicate cells in mitosis.

C. Gating of Hoechst-Low or Hoechst-High cell populations at Day 8 in the micro-patterned culture and each rate of SFTPC^{GFP} -positive cells.

D. A histogram of fluorescence intensity of Hoechst-stained cell colonies derived from ChiPSC18 iPSC in the micro-patterned culture analyzed using FACS. The rate of Hoechst-low cells was defined as the percentage equivalent to that of Hoechst-high cells on the opposite side of the histogram.

E. Representative gene expression levels of micro-patterned ChiPSC18 iPSC measured using qRT-PCR (n=4 independent experiments). AT2 marker genes were enriched in the Hoechst-high periphery of the cell colonies.

F. Comparison of differentially expressed genes (DEGs) between Edge and Center cell populations in micro-patterned cultures. The transcriptomes of the Edge and Center cells in the micro-patterned culture were compared with those of lung progenitors (LPs), and DEGs were defined as genes satisfying the following criteria: padj < 0.01, |log2 fold change| > 1.

G. 667 genes were identified as DEGs common across all time points and colony regions. Enrichment analysis was performed on these DEGs to determine cell type specificity.

H. GSEA analysis of "HE LIM Fetal lung C1 early airway progenitor cell" and heatmaps of their leading-edge genes that appear in the ranked list at or before the point at which the running sum reaches its maximum deviation from zero. Data from the micro-patterned Day 14 samples ranked by P-value comparing Center and Edge cell populations using DESeq2.

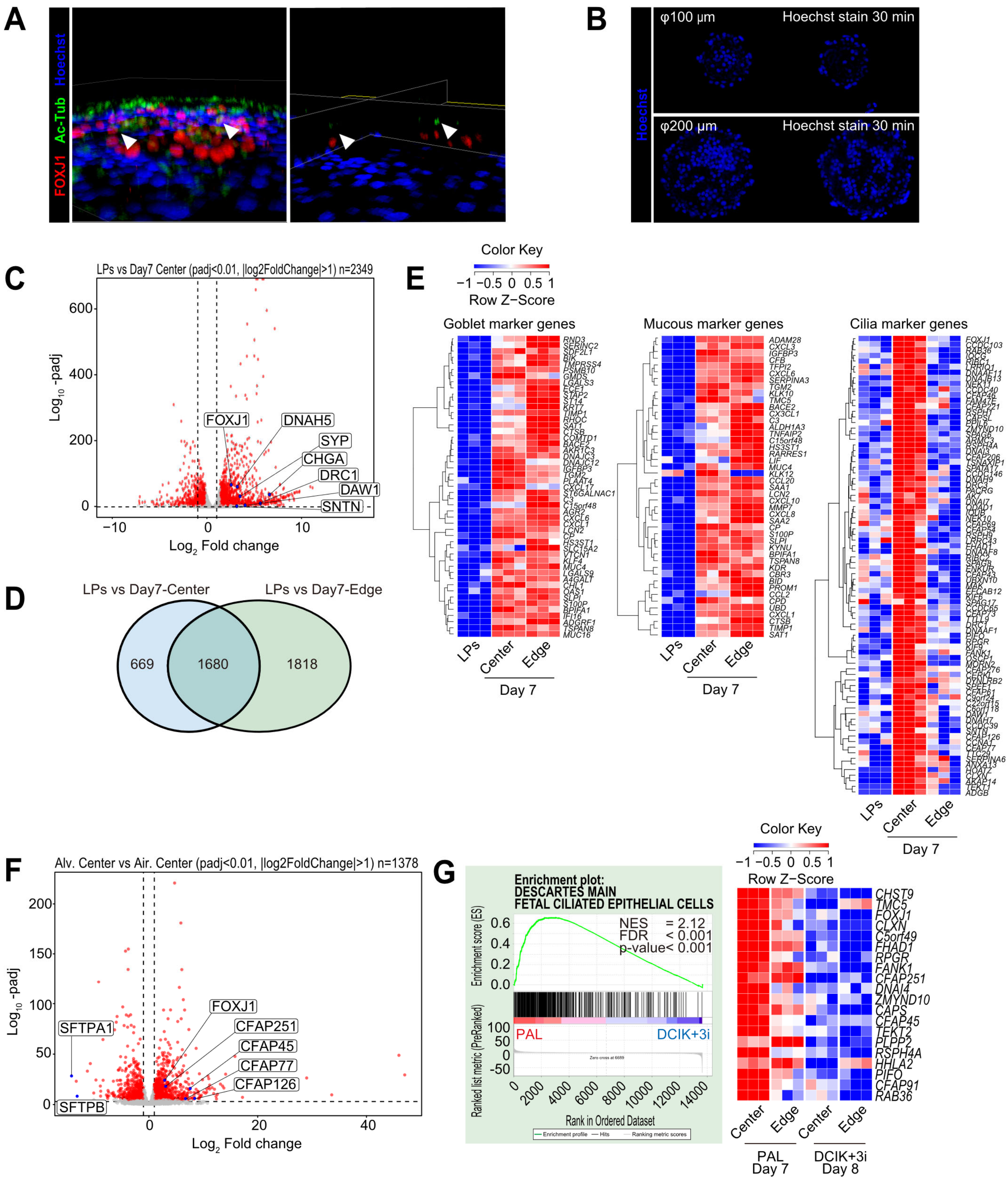
A
FOXJ1 Ac-Tub Hoechst
B
φ100 μm
Hoechst stain 30 min
φ200 μm
Hoechst stain 30 min
Hoechst
C
LPs vs Day7 Center (padj<0.01, |log2FoldChange|>1) n=2349
FOXJ1
DNAH5
SYP
CHGA
DRC1
DAW1
SNTN
Log10 -padj
Log2 Fold change
D
LPs vs Day7-Center
LPs vs Day7-Edge
669
1680
1818
E
Color Key
Row Z−Score
Goblet marker genes
Mucous marker genes
Cilia marker genes
LPs
Center
Edge
Day 7
F
Alv. Center vs Air. Center (padj<0.01, |log2FoldChange|>1) n=1378
SFTPA1
SFTPB
FOXJ1
CFAP251
CFAP45
CFAP77
CFAP126
Log10 -padj
Log2 Fold change
G
Enrichment plot:
DESCARTES MAIN
FETAL CILIATED EPITHELIAL CELLS
NES = 2.12
FDR < 0.001
p-value< 0.001
Enrichment score (ES)
Ranked list metric (PreRanked)
PAL
DCIK+3i
Rank in Ordered Dataset
Enrichment profile
Hits
Ranking metric scores
Color Key
Row Z−Score
CHST9
TMC5
FOXJ1
CLXN
C5orf49
FHAD1
RPGR
FANK1
CFAP251
DNAI4
ZMYND10
CAPS
CFAP45
TEKT2
PLPP2
RSPH4A
HHLA2
PIFO
CFAP91
RAB36
Center
Edge
Center
Edge
PAL
Day 7
DCIK+3i
Day 8

Figure S3. Multiciliated cells cultured in micro-patterned culture plates face the apical side outward. Related to Figure 3.

A. 3D reconstructed imaging of the airway epithelial cells cultured in the micro-patterned plates. Acetylated tubulin (Ac-Tub: arrowhead) is expressed on the colony surface.

B. Live cell imaging of the Hoechst-stained micro-patterned airway cell colonies on Day 14. The nuclei of the multiciliated cells in the center and those in the periphery of the colonies were strongly stained with Hoechst.

C. Volcano plot from DESeq2 analysis comparing LPs to Day 7 Center cell population (n=3 independent experiments).

D. Venn diagram of the two DEG groups. DEGs of LPs vs. Day 7 Center cells were compared with the other DEGs of LPs vs. Day 7 Edge cells.

E. Heatmaps presented with Z-scores of goblet, mucous, or cilia epithelial marker genes. Z-scores were calculated from log (TPM value) (n = 3 independent experiments).

F. Volcano plot from DESeq2 analysis to compare the center populations from DCIK+3i culture (Alv.) and PAL culture (Air.). (n = 3 independent experiments).

G. GSEA analysis of "Descartes Main Fetal Ciliated Epithelial Cells" and heatmaps of their leading top 20 -edge genes. Data from micro-patterned samples collected on Day 7 or 8 were used. These samples were ranked based on P-values obtained by comparing the central populations of Alv. cells and Air. cells using DESeq2.

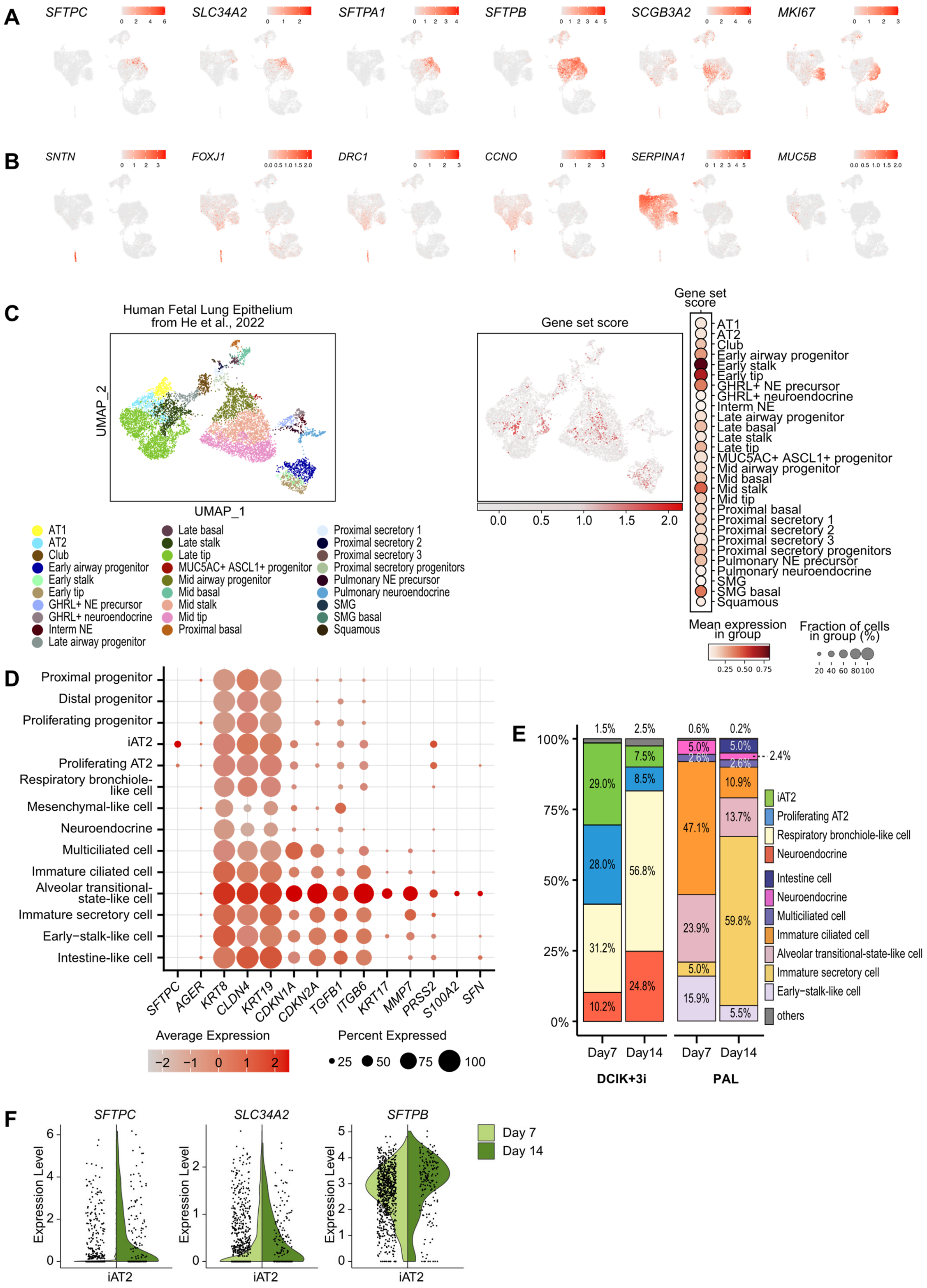
A
SFTPC
SLC34A2
SFTPA1
SFTPB
SCGB3A2
MKI67
B
SNTN
FOXJ1
DRC1
CCNO
SERPINA1
MUC5B
C
Human Fetal Lung Epithelium
from He et al., 2022
UMAP_2
UMAP_1
Gene set score
Gene set score
AT1
AT2
Club
Early airway progenitor
Early stalk
Early tip
GHRL+ NE precursor
GHRL+ neuroendocrine
Interm NE
Late airway progenitor
Late basal
Late stalk
Late tip
MUC5AC+ ASCL1+ progenitor
Mid airway progenitor
Mid basal
Mid stalk
Mid tip
Proximal basal
Proximal secretory 1
Proximal secretory 2
Proximal secretory 3
Proximal secretory progenitors
Pulmonary NE precursor
Pulmonary neuroendocrine
SMG
SMG basal
Squamous
Mean expression in group
Fraction of cells in group (%)
D
Proximal progenitor
Distal progenitor
Proliferating progenitor
iAT2
Proliferating AT2
Respiratory bronchiole-like cell
Mesenchymal-like cell
Neuroendocrine
Multiciliated cell
Immature ciliated cell
Alveolar transitional-state-like cell
Immature secretory cell
Early−stalk-like cell
Intestine-like cell
SFTPC AGER KRT8 CLDN4 KRT19 CDKN1A CDKN2A TGFB1 ITGB6 KRT17 MMP7 PRSS2 S100A2 SFN
Average Expression
Percent Expressed
E
DCIK+3i
PAL
Day7 Day14 Day7 Day14
1.5% 2.5% 0.6% 0.2%
29.0% 28.0% 31.2% 10.2%
7.5% 8.5% 56.8% 24.8%
5.0% 2.6% 47.1% 23.9% 5.0% 15.9%
5.0% 2.6% 10.9% 13.7% 59.8% 5.5%
2.4%
iAT2
Proliferating AT2
Respiratory bronchiole-like cell
Neuroendocrine
Intestine cell
Neuroendocrine
Multiciliated cell
Immature ciliated cell
Alveolar transitional-state-like cell
Immature secretory cell
Early−stalk-like cell
others
F
SFTPC
SLC34A2
SFTPB
Expression Level
iAT2
Day 7
Day 14

Figure S4. Cellular composition and characteristics of lung epithelial cells in micro-patterned cultures. Related to Figure 4.

A and B. UMAP visualizations of gene expression patterns for iAT2-related (A) and airway epithelial-related (B) genes from single-cell RNA-seq data. Color intensity reflects expression levels.

C. UMAP visualization of human fetal lung epithelial cells derived from scRNA-Seq data in He et al., 2022. Left panel: color-coded by cell type; right panel: color-coded gene set score, calculated from 30 marker genes of the "Early-stalk-like cell" cluster identified in this study's scRNA-Seq analysis, with a Dot Plot accompanying it to the side.

D. Dot plot showing transitional AT2-related gene expressions in various cell types. X-axis presents selected genes; y-axis categorizes cell types. Dot size indicates the proportion of cells expressing each gene, and color intensity represents the average expression magnitude.

E. Stacked bar chart depicting the cell type composition on Day 7 and Day 14 for samples cultured in DCIK+3i (Alv.) and PAL media (Air.). Colored segments represent distinct cell types, with respective proportions displayed within each segment.

F. Violin plots overlaid with scatter plots depicting the expression of AT2-related genes in iAT2 cells on Day 7 (light green) and Day 14 (dark green). Violin plots represent data distribution, and scatter plots show individual expression levels at each time point.

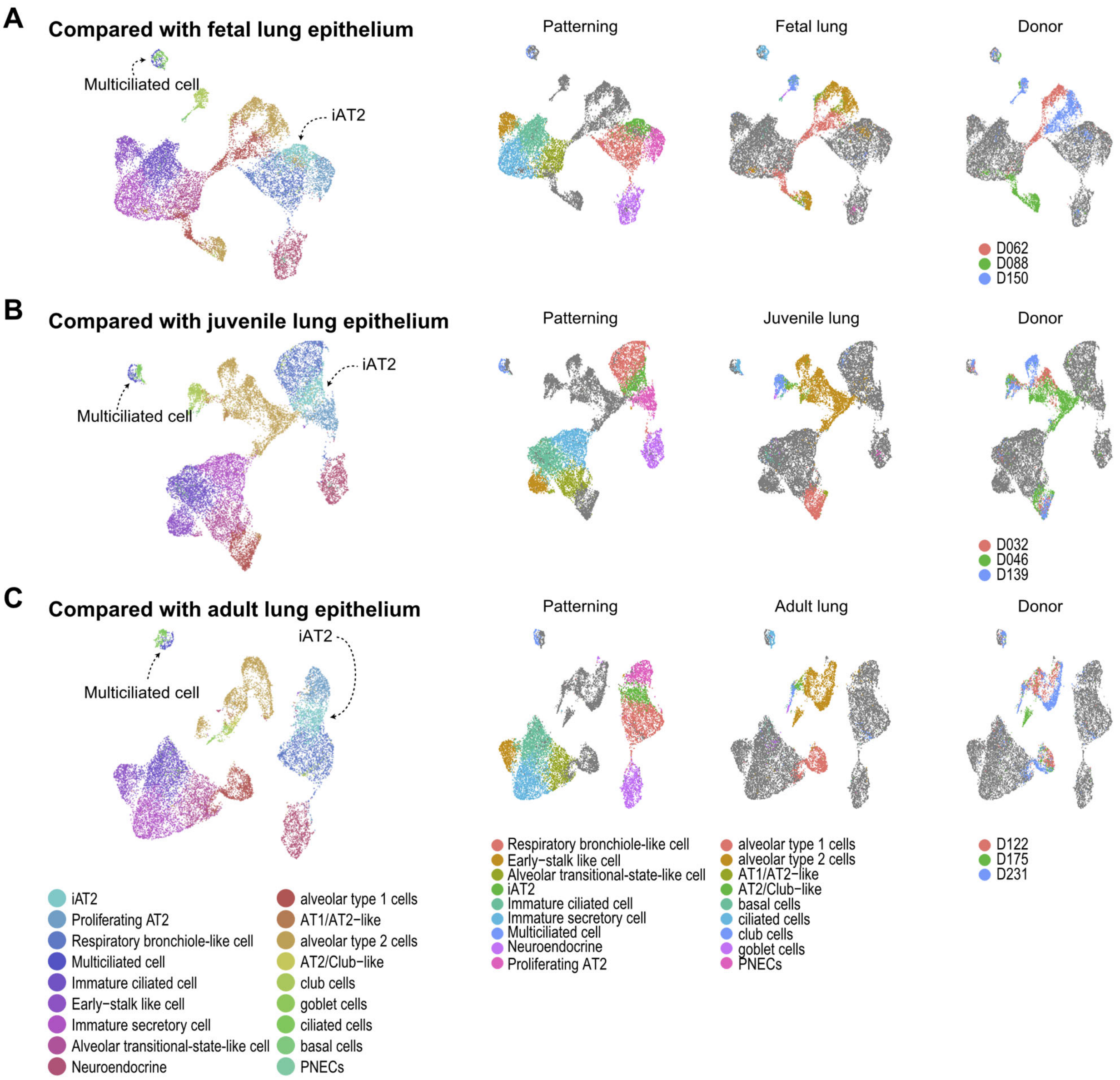

Figure S5. Overview of scRNA-seq transcriptomes of lung epithelial cells induced in micro-patterned culture in comparison with snRNA-Seq ones of primary lung epithelial cells (GSE161383). Related to Figure 4.

A, B, and C. UMAP visualization integrating scRNA-seq transcriptomes of the cells in the micro-patterned culture and snRNA-seq data from fetal (A), juvenile (B), and adult (C) cells, respectively. The annotated snRNA-seq data were obtained from GSE161383[1].

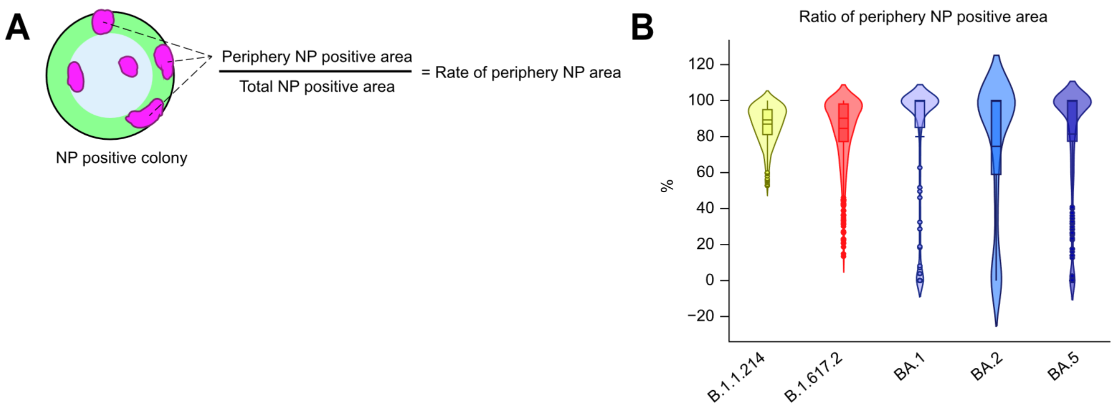

Figure S6. Quantification of the peripheral SARS-CoV-2 NP area ratio. Related to Figure 6.

A. Schematic diagram for quantifying the peripheral SARS-CoV-2 NP area ratio defined as the percentage of SARS-CoV-2 NP signal area in the periphery of the colonies. The total SARS-CoV-2 NP positive area was defined as the area of SARS-CoV-2 NP signals in the positive colonies.

B. Violin plot of the peripheral SARS-CoV-2 NP positive area ratio for alveolar epithelial cells infected with each variant. Measurements were performed by counting only SARS-CoV-2 NP-positive colonies. The number of colonies ranged from 105 to 974.

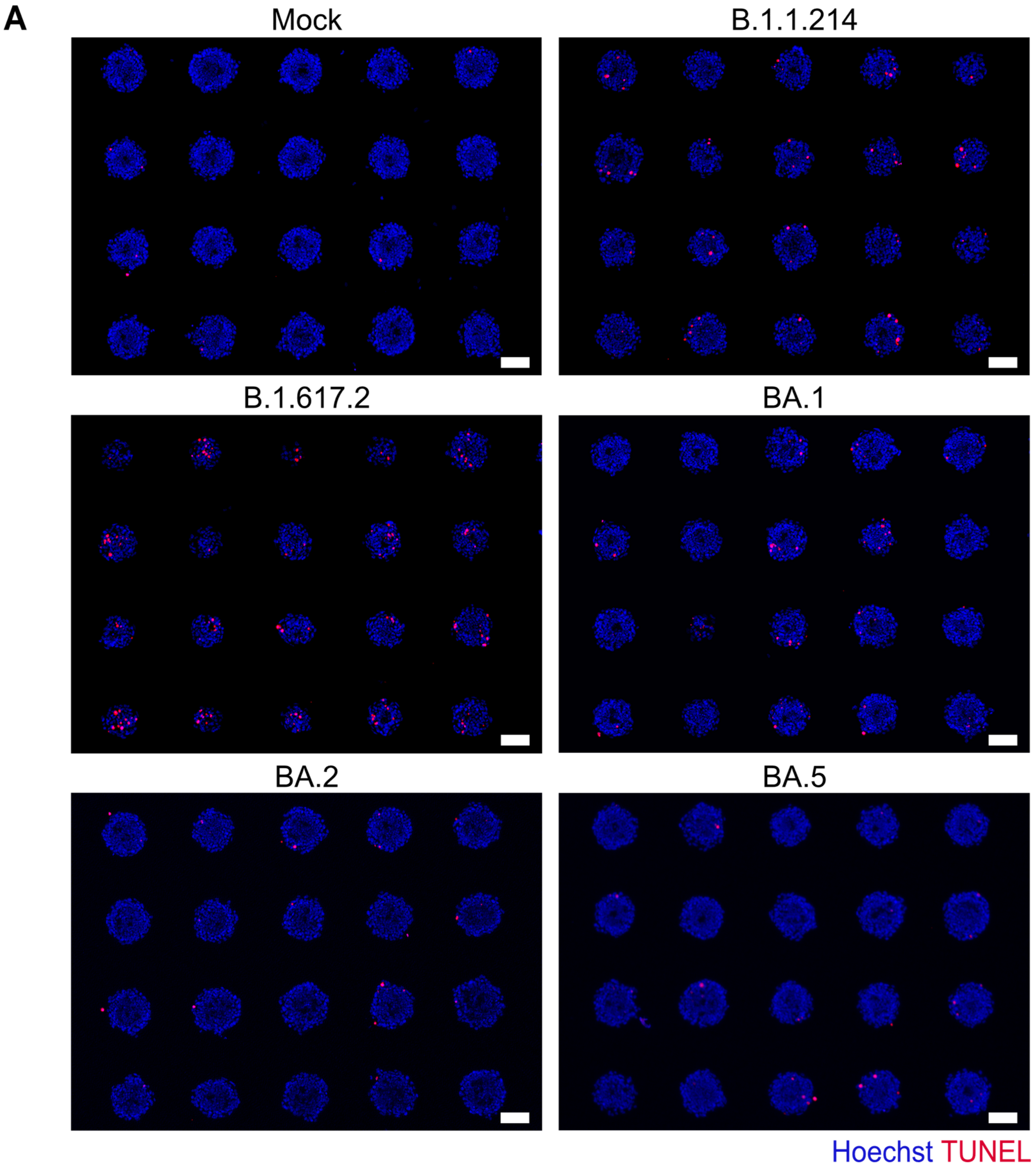

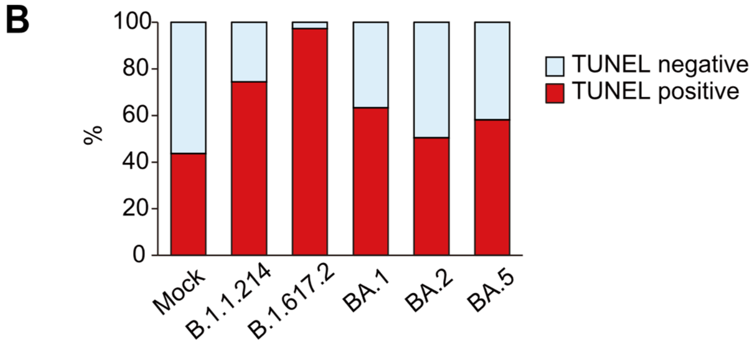

Variant	Total spot	TUNEL positive rate
Mock	1140	43.7 %
B.1.1.214	1169	74.4 %
B.1.617.2	1141	97.3 %
BA.1	1137	63.3 %
BA.2	1141	50.5 %
BA.5	1133	58.2 %

Figure S7. B.1.617.2 variant induces apoptosis of the airway epithelial cells. Related to Figure 7.

A. TUNEL staining images of the airway epithelial cells at 4 dpi with SARS-CoV-2 in the micro-patterned plate. Scale bar: 100 μm.

B. Ratio of TUNEL-positive iPSC-derived airway epithelial cell colonies infected with SARS-CoV-2 in micro-patterned culture. The number of colonies ranged from 1,133 to 1,169 in one representative experiment.

Supplemental Tables

Table S1. Genes are highly expressed in each colony region. Related to Figure 4.

DCIK+3i medium (Alv.)		PAL (Air.)	
**Center**	**Edge**	**Center**	**Edge**
CLDN5	*ICAM1*	*TMEM176B*	*TAGLN*
HES6	*WIF1*	*ASRGL1*	*SCEL*
HEPACAM2	*NMU*	*LIFR*	*VIM*
RGS4	*NRK*	*ST6GALNAC3*	*IGFBP4*
BTBD17	*FGG*	*SMOC2*	*COL4A2*
DLL3	*COL3A1*	*SOX21-AS1*	*FHL2*
SULF2	*PLAU*	*ELAPOR1*	*MMP7*
IGFBP5	*CDKN1A*	*MEIS1*	*GLIPR1*
RET	*CD55*	*LINC02381*	*ADAM8*
DLL1	*ME1*	*LRIG1*	*CYP1A1*
CRIP2	*CCNA2*	*DACT2*	*CD9*
CBFA2T2	*LAMC2*	*GSTA4*	*FSTL3*
BRINP2	*TK1*	*CFI*	*TNFRSF12A*
TFF3	*KIF23*	*PCDH19*	*LAMC2*
MIAT	*IL32*	*DMD*	*SH3BGRL3*
PRDM16	*SLPI*	*LINC00261*	*TM4SF1*
EGR2	*NSG1*	*TEKT2*	*AREG*
SUSD4	*PGC*	*SPON1*	*BCAT1*
ADM	*SLC34A2*	*SEMA6A*	*INHBA*
DAPK1	*DLC1*	*ELOVL2*	*CLDN7*
VCAN	*NAPSA*	*SCARA3*	*SFXN3*
NIBAN1	*GK*	*ZMYND10*	*IDS*
LRIG1	*SFTPC*	*CCNO*	*LAYN*
PGAP4	*TSPAN7*	*COL2A1*	*MFGE8*
GLB1L2	*ROS1*	*TPD52L1*	*AKAP12*
MYCL	*F3*	*LRAT*	*COL6A2*
ZFP36L2	*THBD*	*ASCL1*	*RAB11FIP1*
NPHP1	*ANKRD1*	*SERPINI1*	*KRT7*
SERPINF1	*EMP2*	*GPC3*	*TRIM16*
ADRA2A	*TUBB6*	*GSTA1*	*GAS6*

Table S2. Primers for Taqman qRT-PCR

Gene	Taqman ID
18S rRNA	Hs99999901_s1
AGER	Hs00153957_m1
MUC5B	Hs00861595_m1
SFTPC	Hs00161628_m1

Table S3. Primers for SYBR qRT-PCR

Gene	Forward primer sequence (5′–3′)	Reverse primer sequence (3′–5)
ACE2	ACAGTCCACACTTGCCCAAAT	TGAGAGCACTGAAGACCCATT
FOXJ1	CCTGTCGGCCATCTACAAGT	AGACAGGTTGTGGCGGATT
GAPDH	GGAGCGAGATCCCTCCAAAAT	GGCTGTTGTCATACTTCTCATGG
IFNA1	GCCTCGCCCTTTGCTTTACT	CTGTGGGTCTCAGGGAGATCA
IFNB1	ATGACCAACAAGTGTCTCCTCC	GGAATCCAAGCAAGTTGTAGCTC
MX1	CTTATCCGTTAGCCGTGGTG	CAAGGTGGAGCGATTCTGAG
PDPN	TCCAGGAACCAGCGAAGAC	CGTGGACTGTGCTTTCTGA
SARS-CoV-2	AGCCTCTTCTCGTTCCTCATCAC	CCGCCATTGCCAGCCATTC
SARS-CoV-2 N	CCAGGTAACAAACCAACCAACTTTCG	GGTTACTGCCAGTTGAATCTGAGG
SCGB1A1	CACCATGAAACTCGCTGTCAC	AGTTCCATGGCAGCCTCATAAC
SCGB3A2	CAAGTGGAACCACTGGCTTG	CCAGAGGTAAAGGTGCCAAC
SFTPB	GAGCCGATGACCTATGCCAAG	AGCAGCTTCAAGGGGAGGA
SLC34A2	TCGCCACTGTCATCAAGAAG	CTCTGTACGATGAAGGTCATGC

SNTN	GCTGCAAACCCAATTTAGGA	TGCTCATCAAGTTCAGAAAGGA
TMPRSS2	GTCCCCACTGTCTACGAGGT	CAGACGACGGGGTTGGAAG
TP63	ACTGCCAAATTGCAAAGACA	TGACTAGGAGGGGCAATCTG

Supplemental videos

Video S1. Human iPSC-derived LPs cultured in the micro-patterned plate for 14 days to achieve airway differentiation involving multiple cilia observed at the center of the colony, related to Figure 3.

Video S2. 3D reconstructed imaging of the alveolar epithelial cells cultured in the micro-patterned plate, related to Figure S1B.

SUPPLEMENTAL EXPERIMENTAL PROCEDURES

Human iPSC culture

All cells were cultured in 5% CO_2 at 37 °C. Human iPSCs were cultured as described previously [2]. Briefly, *SFTPC*^{GFP} reporter iPSC (B2-3) [3] and ChiPSC18 (Takara Bio, Y00300) were maintained in Essential 8 medium (Thermo Fisher Scientific, A1517001) on a 6-cm dish coated with Geltrex (Thermo Fisher Scientific, A1413201) and the media was changed daily. The cells were passaged at an appropriate split ratio (1:6–1:10) once they achieved approximately 80–90% confluency. Thereafter, 10 µm Y27632 was added to the medium for 24 h. Each iPSC line was grown to 100% confluence to differentiate into NKX2-1+ LPs. Both *SFTPC*^{GFP} reporter iPSC (B2-3) and ChiPSC18 were exempt from ethical approval.

Differentiation of human iPSCs into NKX2-1+ LPs

Human iPSCs were differentiated stepwise into lung progenitor cells (LPs), as previously described [2–4]. Briefly, human iPSCs were differentiated into definitive endoderm (DE) on Geltrex-coated plates in RPMI-1640 medium (Nacalai Tesque, 30264–56) containing 100 ng/mL activin A (API, GF-001-050L), 1 µM CHIR-99021 (Axon medchem, CT99021), 2% B27 supplement (Thermo Fisher Scientific, 17504–001), and 50 U/mL Penicillin/streptomycin (Thermo Fisher Scientific, 15140-122). Sodium butyrate (Fujifilm Wako, 193–01522) was added to a final concentration of 0.25 mM 24 h after seeding (Day 1). From days 2 to 6, the cells were cultured in RPMI-1640 medium containing 100 ng/mL activin A, 1 µM CHIR-99021, 2% B27 supplement, 50 U/mL Penicillin/streptomycin, and 0.125 mM sodium butyrate. In subsequent steps, DMEM/F12 (Thermo Fisher Scientific, 10565-042) medium supplemented with GlutaMAX, 2% B-27 supplement, 50 U/mL Penicillin/streptomycin, 0.05 mg/mL L-ascorbic acid (Fujifilm Wako, 016-04805), and 0.4 mM monothioglycerol (Fujifilm Wako, 195-15791) was used as basal medium. From days 6 to 10, DE cells were cultured in a medium supplemented with 100 ng/mL noggin (R&D systems, 6057-NG-01M) and 10 µM SB431542 (Fujifilm Wako, 198-16543) to differentiate into anterior foregut endoderm (AFE) cells. The AFE cells were cultured in the medium supplemented with 3-µM CHIR99021, cell-specific optimized concentrations of all-trans retinoic acid (B2-3: 0.05 µM, ChiPSC18: 1 µM) (Sigma-Aldrich, R2625), and 20 ng/mL BMP4 (Proteintech, HZ-1078) for days 10–14 to differentiate into ventralized anterior foregut endoderm (VAFE) cells. VAFE cells were efficiently distalized in the medium supplemented with 3 µM CHIR99021, 10 ng/mL FGF10 (Pepro Tech, 100-26), 10 ng/mL KGF (Pepro Tech, 100-19), and 20 µM DAPT (Fujifilm Wako, 049-33583) for 7 days. On days 20–22, NKX2-1+ LPs were isolated using an autoMACS Pro separator (Miltenyi Biotec) with mouse anti-human carboxypeptidase M (CPM) (Fujifilm Wako, 014-27501) and anti-mouse IgG microbeads (Miltenyi Biotech, 130-048-402).

SARS-CoV-2 preparation

SARS-CoV-2 strains B.1.1.214 (GISAID accession number: EPI_ISL_2897162), B.1.617.2 (EPI_ISL_9636792), BA.1 (EPI_ISL_9638489), BA.2 (EPI_ISL_11900505), and BA.5 (EPI_ISL_14018093) were isolated from the nasopharyngeal swab samples of patients with COVID-19[5]. This study was approved by the research ethics committee of Kyoto University (R2379-3). Viruses were produced in TMPRSS2/Vero cells (JCRB1818, JCRB Cell Bank) [6] and stored at -80 °C until use. TMPRSS2/Vero cells were cultured in minimum essential medium (MEM, Sigma-Aldrich, F0385-500ML) supplemented with 5% fetal bovine serum and 1% penicillin/streptomycin. All live virus experiments were performed in a biosafety level 3 facility at Kyoto University, following strict regulations.

Transmission electron microscopy

Cells were cultured for 14 days in DCIK+3i medium on micro-patterned culture plates. The cells were incubated in Accutase (Funakoshi, AT104-500) at 37 °C for 20 min and detached through gentle pipetting. The cell suspension was centrifuged at 200x *g* for 10 min at 4 °C. The cell pellet was embedded in Matrigel. The pellet-embedded Matrigel was incubated in a fixative solution comprising 2.5% glutaraldehyde (Nacalai Tesque, 17003-92), 4% paraformaldehyde (Nacalai Tesque, 26126-54), 0.1% picric acid, 4% sucrose (Nacalai Tesque, 30404-45) and 0.1 M phosphate buffer (pH 7.4) at 4 °C for 2 h, followed by incubation in 1% uranyl acetate en bloc at room temperature for 1 h[7]. The samples were washed in an ascending concentration of ethanol and propylene oxide and embedded in Epon 812. Thin sections were doubly stained with uranyl acetate and lead citrate and examined under a Hitachi H-7650 transmission electron microscope.

Live cell imaging
Phase-contrast and fluorescence images and movies were obtained using a BZ-X710 or BZ-X810 microscope (Keyence). Images were analyzed using a BZ-X or BZ-X800 analyzer, and the Image Cytometer Module was used to quantify the number of GFP-positive colonies and the percentage of GFP-positive areas.

Live cell fluorescence staining
The cells on the micro-pattern culture plates were incubated for up to 24 h under 5% CO_2 at 37 °C in a medium containing Hoechst-33342 (1:500), 20nM TMRM (Thermo Fisher Scientific, I34361), or 50nM LysoTracker Deep Red (Thermo Fisher Scientific, L12492). Fluorescence images were obtained using a BZ-X710 system.

Flow cytometry
Cell suspension and washing were performed using a flow cytometry buffer: PBS (1% BSA and 100 μM Y-27632). The cells were incubated in Accutase at 37 °C for 20 min and detached through gentle pipetting. The cells cultured on micro-pattern culture plates were first stained with Hoechst-33342 (1:500) for 30 min at 37°C, followed by incubation in Accutase at 37 °C for 20 min and dissociation through gentle pipetting. To define the Hoechst-high population, unstained cell suspensions were first dissociated using the same procedure, followed by staining with Hoechst (1:500) for 30 min at 37°C. All cell suspensions were washed and resuspended in flow cytometry buffer supplemented with PI solution (1:1000; Dojindo, 341-07881). Hoechst-low (center) and Hoechst-high (Edge) populations were separated according to the intensity of Hoechst using FACS Aria III (BD Biosciences). The Hoechst-high population was defined and compared with the fluorescence intensity of the Hoechst-stained sample in suspension. The Hoechst-low population was sorted from the opposite side of the peak with a similar percentage of cells to the Hoechst-high population. After isolation, the cells were centrifuged at 170x *g* for 10 min at 4 °C to remove the supernatant, lysed with the RLT buffer of the RNeasy Micro Kit (Qiagen, 74004) and stored at -80°C until total RNA extraction for RNA-seq or qRT-PCR analysis.

Immunofluorescence analysis
The cells on the micro-patterned culture plates were fixed in 4% paraformaldehyde/PBS heated to 37 °C and incubated at room temperature for 15 min. The fixed cells were stored at 4 °C in PBS until staining. The samples were permeabilized with 0.2% Triton X-100 (Nacalai Tesque, 12967-32)/PBS and blocked with a blocking buffer of PBS containing 5% normal donkey serum (EMD-Millipore, 566460) and 1% BSA (Sigma-Aldrich, A9647). Furthermore, the samples were stained overnight at 4 °C with the following primary antibodies diluted in the blocking buffer: chicken anti- GFP antibody (1:500, Aves Labs, #GFP-1020), mouse anti-NaPi2b (1:100, kindly provided by Dr. Gerd Ritter (MX35)), rabbit anti-SPB (1:500, Abcam, ab40876), mouse anti-FOXJ1 (1:500, R.T. 1hr, Thermo Fisher Scientific, 14-9965-82), mouse anti-Acetylated Tubulin (1:4000, R.T. overnight, Sigma-Aldrich, T7451), mouse anti-WIF1 (1:50, Santa Cruz Biotechnology sc-373780), rabbit anti-DLL3 (1:50, Cell Signaling Technology #71804) and rabbit anti-SARS-CoV-2 Nucleocapsid (1:200, SinoBiological 40588-T62). Subsequently, the samples were stained for 1 h at R.T. with Hoechst-33342 (1:1000, Dojindo H342) and the following secondary antibodies: AlexaFluor546-conjugated donkey anti-rabbit IgG (1:500, Thermo Fisher Scientific #A-10040), AlexaFluor647-conjugated donkey anti-rabbit IgG (1:500, Thermo Fisher Scientific #A-31573), AlexaFluor546-conjugated donkey anti-mouse IgG (1:500, Thermo Fisher Scientific #A-10036), and AlexaFluor488-conjugated donkey anti-chicken IgY (1:500, Jackson Immuno Research #703-485-155). Nuclear staining was performed by Hoechst-33342 or SYTO61 Red Fluorescent Nucleic Acid Stain (1 mM, Thermo Fisher Scientific S11343). TUNEL staining was performed using the DeadEnd Fluorometric TUNEL System (Promega, G3250). Staining was performed according to the manufacturer's instructions. A BZ-X710 or a TCS SP8 confocal microscope (Leica microsystems) was used for imaging.

Image analysis of the position-specific signals
The channel merge images captured using the Keyence all-in-one fluorescence microscope BZ-X710 were processed with Python 3.9.13. The center coordinates of the colonies were determined from the binarized merged images using the center_of_mass function from SciPy (version 1.11.4). For all pixels in each channel-separated image, the distance was calculated from the center coordinates and intensity. The intensity of pixels at equal distances from the center was averaged, and the data were sorted based on distance. Each channel underwent normalization, setting the maximum Intensity to 1 and the minimum Intensity to 0; the results were plotted as line graphs in the same plot area.

qRT-PCR
The PureLink RNA Mini Kit (Thermo Fisher Scientific, 12183025) or the RNeasy Micro Kit (Qiagen, 74004) was used to extract total RNA, depending on the experiment. Total RNA was reverse-transcribed using the ReverTra Ace qPCR RT Master Mix with gDNA Remover (TOYOBO, FSQ-301) according to the manufacturer's protocol. The qRT-PCR was performed on a StepOnePlus Real-Time PCR System using Power SYBR Green PCR Master Mix (Thermo Fisher Scientific, 4368577) or THUNDERBIRD Probe qPCR Mix (TOYOBO, QPS-101). Primers used in this study are listed in Table S2. Gene expression was normalized to eukaryotic 18S rRNA and compared with that of the human adult lung 5 donor pool (BioChain, #R1234152-P, lot A811037) or each control.

SARS-CoV-2 genome copy in culture supernatant quantification

The cell culture supernatant was mixed with an equal volume of 2×RNA lysis buffer (distilled water containing 0.4 U/μL SUPERase In^{TM} RNase Inhibitor (Thermo Fisher Scientific, AM2694), 2% Triton X-100, 50 mM KCl, 100 mM Tris-HCl (pH 7.4), and 40% glycerol) and incubated at room temperature for 10 min. The mixture was diluted 10 times with distilled water. For quantifying SARS-CoV-2 RNA, the One-Step TB Green PrimeScript PLUS RT-PCR Kit (Perfect Real Time) (Takara Bio, RR096A) was used on a QuantStudio 1 or QuantStudio 3 real-time PCR system (Thermo Fisher Scientific). Standard curves were prepared using SARS-CoV-2 RNA (10^5 copies/μL) purchased from Nihon Gene Research Laboratories. The primer sequences are presented in Table S3.

Image analysis of SARS-CoV-2 infection samples

The cells grown on the micro-patterned culture plates were fixed in 4% PFA after treatment with SARS-CoV-2 infection and subjected to immunostaining for various marker proteins. Fluorescence images were captured for each well using a BZ-X810 with the Imaging Cytometer Module. The fluorescence intensity, number, and size in each colony were obtained from the images captured using a BZ-X800 Analyzer. The acquired data were processed using the KNIME Analytics Platform (Infocom). The sizes of the colonies and the areas of the various epithelial cell marker signals were normalized, and data with |Z-score| > 3 were excluded as outliers. The SARS-CoV-2 variant NP area in the colony was normalized for each infection group, and data with a |Z-score| > 3 were excluded as outliers. An average of 10.1% of the data were excluded from this process. The remaining data were used for the quantitative analyses.

qRT-PCR of SARS-CoV-2-infected cells

Total RNA was isolated from the infected cells at 4 dpi using ISOGEN (NIPPON GENE, 319-90211). cDNA was synthesized from 500 ng of total RNA using the Superscript VILO cDNA Synthesis Kit (Thermo Fisher Scientific, 11754050). Real-time RT-PCR was performed with the SYBR Green PCR Master Mix (Thermo Fisher Scientific, 4344463) using the StepOnePlus Real-Time PCR System, QuantStudio 1, or QuantStudio 3 Real-Time PCR System (Thermo Fisher Scientific). The relative quantification of target mRNA levels was performed using the $2^{-\Delta\Delta CT}$ method. Values were normalized to the housekeeping gene glyceraldehyde 3-phosphate dehydrogenase (GAPDH). The PCR primer sequences are shown in Tables S1-S2.

RNA-seq of the micro-patterned cells

Total RNA was extracted using the RNeasy Micro Kit according to the manufacturer's protocol. The RNA integrity of each sample was confirmed to be >8 using the 2100 BioAnalyzer. To analyze the Hoechst-high and Hoechst-low cell populations, 1 ng of the total RNA was reverse transcribed and amplified for 11 cycles using SMART-Seq HT (Clontech Laboratories, Z4456N). The amplified cDNA was used to prepare sequencing libraries with a Nextera XT DNA Library Preparation Kit (Illumina, FC-131). Library sequencing was performed using a NovaSeq 6000 or NextSeq2000 with the 100 bp paired-end method. Finally, raw data from this study were submitted to the Gene Expression Omnibus (GEO) under the accession number GSE236839 and GSE236840.

RNA-seq of the SARS-CoV-2-infected cells

Total RNA was isolated using the ISOGENE. The integrity of the RNA was assessed using the 2100 Bioanalyzer (Agilent Technologies). Similarly, library preparation was performed using the TruSeq Stranded mRNA Sample Prep Kit (Illumina, 20020594) according to the manufacturer's instructions. Furthermore, we performed sequencing using Illumina NextSeq550. We generated the FASTQ files using bcl2fastq-2.20. Adapter sequences and low-quality bases were trimmed from the raw reads using Cutadapt ver v3.4 [8]. Mapping of the trimmed reads to human reference genome sequences (hg38) was conducted using STAR version 2.7.9a [9] with a GENCODE (release 36, GRCh38.p13) GTF file [10]. We calculated the raw counts using the htseq-count ver. 0.13.5 [11] with the GENCODE GTF file. Gene expression levels were determined as transcripts per kilobase million (TPM) using DESeq2 v1.30.1 [12] . Finally, raw data from this study were submitted to the Gene Expression Omnibus (GEO) under the accession number GSE236841.

Bioinformatic analysis of RNA-seq data

Sequenced reads of the Hoechst-low (Center) or Hoechst-high (Edge) cells were trimmed using fastp [13], and the trimmed reads were aligned to GRCh38 using STAR 2.7.1a [9]. Transcript per million (TPM) values were calculated using RSEM [14]. Low expression genes with average TPM values among the comparison data set of less than 1 were excluded from downstream analyses. Principal component analysis of log2 (TPM+0.01) was performed using the R function "prcomp" and visualized using the R package "ggplot2." The R package DESeq2 [12] was used to identify DEGs. Volcano plots were visualized using the R package "ggplot2" and "ggrepel," and Venn diagrams for multiple DEGs were visualized using the R package "Venn Diagram." GSEA [15] was performed using genes ordered by P-values calculated using DESeq2. Enrichment analysis for GO based on biological processes was performed using the Metascape online software[16]. A heatmap using log2 (TPM+0.01) was visualized by the R package "gplots."

scRNA-seq of the micro-patterned colony cells

The cell suspensions were prepared via enzymatic dissociation; the micro-patterned cells were washed in PBS pre-warmed to 37 °C and immersed in 0.5 mM EDTA/PBS at 37 °C for 12 min. Subsequently, the cells were incubated with Accutase at 37 °C for 25 min and dissociated via gentle pipetting. LPs were isolated from the cell suspensions using MACS-based isolation of CPM-positive cells. Single-cell RNA libraries for lung epithelial cells and LPs were prepared using a 10X

Genomics Chromium device, according to the manufacturer's protocols specified in the Single Cell 3′ Reagent Kits v3.1. The 10X Genomics Cell Ranger pipeline (version 7.1.0) was used to perform sample demultiplexing, alignment to the hg38 human reference genome (refdata-gex-GRCh38-2020-A from 10X Genomics) and the reporter sequences, barcode/UMI processing, and gene counting for each cell. In the quality control (QC) of sequencing data, dead cells or outliers were identified and excluded based on two criteria: cells with fewer than 200 detected genes and cells with a high percentage of counts mapped to mitochondrial genes, with a threshold set at 15%. We used the Seurat package of the R software for data analysis and visualization. For comparative analysis of scRNA-seq with snRNA-seq, each transcriptome was integrated, and UMAP was drawn according to the 'anchor-based' integration workflow on the official Seurat website (https://satijalab.org/seurat/).

SUPPLEMENTAL REFERENCES

1. Wang, A., Chiou, J., Poirion, O.B., Buchanan, J., Valdez, M.J., Verheyden, J.M., Hou, X., Kudtarkar, P., Narendra, S., Newsome, J.M., et al. (2020). Single cell multiomic profiling of human lung reveals cell type-specific and age-dynamic control of SARS-CoV2 host genes. Elife *9*, 1–28. 10.7554/eLife.62522.

2. Yamamoto, Y., Gotoh, S., Korogi, Y., Seki, M., Konishi, S., Ikeo, S., Sone, N., Nagasaki, T., Matsumoto, H., Muro, S., et al. (2017). Long-term expansion of alveolar stem cells derived from human iPS cells in organoids. Nat. Methods *14*, 1097–1106. 10.1038/nmeth.4448.

3. Gotoh, S., Ito, I., Nagasaki, T., Yamamoto, Y., Konishi, S., Korogi, Y., Matsumoto, H., Muro, S., Hirai, T., Funato, M., et al. (2014). Generation of Alveolar Epithelial Spheroids via Isolated Progenitor Cells from Human Pluripotent Stem Cells. Stem Cell Reports *3*, 394–403. 10.1016/j.stemcr.2014.07.005.

4. Konishi, S., Gotoh, S., Tateishi, K., Yamamoto, Y., Korogi, Y., Nagasaki, T., Matsumoto, H., Muro, S., Hirai, T., Ito, I., et al. (2016). Directed Induction of Functional Multi-ciliated Cells in Proximal Airway Epithelial Spheroids from Human Pluripotent Stem Cells. Stem Cell Reports *6*, 18–25. 10.1016/j.stemcr.2015.11.010.

5. Hashimoto, R., Tamura, T., Watanabe, Y., Sakamoto, A., Yasuhara, N., Ito, H., Nakano, M., Fuse, H., Ohta, A., Noda, T., et al. (2023). Evaluation of Broad Anti-Coronavirus Activity of Autophagy-Related Compounds Using Human Airway Organoids. Mol. Pharm. *20*, 2276–2287. 10.1021/acs.molpharmaceut.3c00114.

6. Matsuyama, S., Nao, N., Shirato, K., Kawase, M., Saito, S., Takayama, I., Nagata, N., Sekizuka, T., Katoh, H., Kato, F., et al. (2020). Enhanced isolation of SARS-CoV-2 by TMPRSS2-expressing cells. Proc. Natl. Acad. Sci. U. S. A. *117*, 7001–7003. 10.1073/pnas.2002589117.

7. Osanai, K., Higuchi, J., Oikawa, R., Kobayashi, M., Tsuchihara, K., Iguchi, M., Huang, J., Voelker, D.R., and Toga, H. (2010). Altered lung surfactant system in a Rab38-deficient rat model of Hermansky-Pudlak syndrome. Am. J. Physiol. Lung Cell. Mol. Physiol. *298*, L243-51. 10.1152/ajplung.00242.2009.

8. Kechin, A., Boyarskikh, U., Kel, A., and Filipenko, M. (2017). cutPrimers: A New Tool for Accurate Cutting of Primers from Reads of Targeted Next Generation Sequencing. J. Comput. Biol. *24*, 1138–1143. 10.1089/cmb.2017.0096.

9. Dobin, A., Davis, C.A., Schlesinger, F., Drenkow, J., Zaleski, C., Jha, S., Batut, P., Chaisson, M., and Gingeras, T.R. (2013). STAR: ultrafast universal RNA-seq aligner. Bioinformatics *29*, 15–21. 10.1093/bioinformatics/bts635.

10. Frankish, A., Diekhans, M., Ferreira, A.-M., Johnson, R., Jungreis, I., Loveland, J., Mudge, J.M., Sisu, C., Wright, J., Armstrong, J., et al. (2019). GENCODE reference annotation for the human and mouse genomes. Nucleic Acids Res. *47*, D766–D773. 10.1093/nar/gky955.

11. Andrews, P.W., Barbaric, I., Benvenisty, N., Draper, J.S., Ludwig, T., Merkle, F.T., Sato, Y., Spits, C., Stacey, G.N., Wang, H., et al. (2022). The consequences of recurrent genetic and epigenetic variants in human pluripotent stem cells. Cell Stem Cell *29*, 1624–1636. 10.1016/j.stem.2022.11.006.

12. Love, M.I., Huber, W., and Anders, S. (2014). Moderated estimation of fold change and dispersion for RNA-seq data with DESeq2. Genome Biol. *15*, 550. 10.1186/s13059-014-0550-8.

13. Chen, S., Zhou, Y., Chen, Y., and Gu, J. (2018). fastp: an ultra-fast all-in-one FASTQ preprocessor. Bioinformatics *34*, i884–i890. 10.1093/bioinformatics/bty560.

14. Li, B., and Dewey, C.N. (2011). RSEM: accurate transcript quantification from RNA-Seq data with or without a reference genome. BMC Bioinformatics *12*, 323. 10.1186/1471-2105-12-323.

15. Subramanian, A., Tamayo, P., Mootha, V.K., Mukherjee, S., Ebert, B.L., Gillette, M.A., Paulovich, A., Pomeroy, S.L., Golub, T.R., Lander, E.S., et al. (2005). Gene set enrichment analysis: a knowledge-based approach for interpreting genome-wide expression profiles. Proc. Natl. Acad. Sci. U. S. A. *102*, 15545–15550. 10.1073/pnas.0506580102.

16. Zhou, Y., Zhou, B., Pache, L., Chang, M., Khodabakhshi, A.H., Tanaseichuk, O., Benner, C., and Chanda, S.K. (2019). Metascape provides a biologist-oriented resource for the analysis of systems-level datasets. Nat. Commun. *10*, 1523. 10.1038/s41467-019-09234-6.
